# Anti-glioma Natural Products Downregulating Tumor Glycolytic Enzymes from Marine Actinomycete *Streptomyces* sp. ZZ406

**DOI:** 10.1038/s41598-017-18484-7

**Published:** 2018-01-08

**Authors:** Mengxuan Chen, Weiyun Chai, Tengfei Song, Mingzhu Ma, Xiao-Yuan Lian, Zhizhen Zhang

**Affiliations:** 1 Ocean College, Zhoushan Campus, Zhejiang University, Zhoushan, 316021 China; 2 College of Pharmaceutical Sciences, Zhejiang University, Hangzhou, 310058 China

## Abstract

Marine natural products are important resources for discovering novel anticancer drugs. In this study, an extract prepared from the culture of a sea anemone-derived actinomycete *Streptomyces* sp. ZZ406 in soluble starch and casein-related liquid medium was found to have activity in inhibiting the proliferation of glioma cells and reducing the production of lactate in glioma cells. Chemical investigation of this active crude extract resulted in the isolation of four new compounds and seven known ones. Structures of the new compounds were determined by a combination of extensive NMR analyses, HRESIMS and MS-MS data, electronic circular dichroism calculation, chemical degradation, and Marfey’s method. New compound **1** showed potent activity against the proliferation of different glioma cells with IC_50_ values of 4.7 to 8.1 μM, high selectivity index (>12.3 to 21.3), and good stability in human liver microsomes. Western blot analysis revealed that compound **1** remarkably downregulated the expressions of several important glioma glycolytic enzymes. The data from this study suggested that compound **1** might have potential as a novel anti-glioma agent to be further investigated.

## Introduction

Gliomas are the most common malignant brain tumors with a high mortality rate^1,2^. Chemotherapy plays a more important role in the treatment and prevention of gliomas as gliomas usually locate at many important brain function areas, which makes the surgical resection very difficult. However, most of the current anti-glioma drugs such as temozolomide (TMZ), carmustine, lomustine, and procarbazine are DNA cytotoxic alkylating agents with limited efficacy and serious toxicity and side-effects^3,4^. Thus, the discovery and development of new anti-glioma drugs with unique mechanism of action is the current top priority. Marine natural products are important resources for discovering novel anticancer drugs^5–7^.

Cancer metabolic reprogramming represents an attractive therapeutic target^8–11^. Enhanced glycolysis is required for the rapid and unlimited proliferation of cancer cells and has been proven as a prominent hallmark in glioma metabolism^8–10^. Several important glycolytic enzymes (regulators) including hexokinase 2 (HK2)^8,9,12^, 6-phosphofructo-2-kinase/fructose-2,6-bisphosphatase (PFKFB3)^8,13^, pyruvate kinase M2 (PKM2)^8,9,14^, and lactate dehydrogenase 5 (LDH5)^8,15^ have been revealed to be upregulated in glioma cells and are preferentially used by cancer cells^8^. Lactate is the end product of enhanced glycolysis in tumor cells and has been shown to increase tumor growth, invasion, and metastasis^15,16^. The reduction of lactate in tumors is related to the regulation of tumor glycolysis and has antitumor activity^17,18^. All the data demonstrate that the tumor glycolytic enzymes of HK2, PFKFB3, PKM2, LDH5 and the lactate are promising targets for the discovery of novel anticancer drugs.

During the course of our ongoing project to discover novel anti-glioma agents from marine resources^19–28^, hundreds of bacteria were isolated from marine resources. Each extract prepared from the cultures of the isolate marine bacteria was assayed by sulforhodamine B (SRB) method for its activities in inhibiting the proliferation of glioma cells and in reducing the production of lactate in glioma cells. It has been found that several extracts had antiproliferative activity and also reduced lactate production in glioma cells, resulting in the discovery of several anti-glioma compounds by downregulating multiple tumor glycolytic enzymes^23,27,28^. These results suggested that the *in vitro* antiproliferative active assay in combination with the determination of lactate production might be a new method to discover novel anti-glioma agents with the unique mechanism of targeting multiple tumor metabolic regulators.

**Figure 1:**
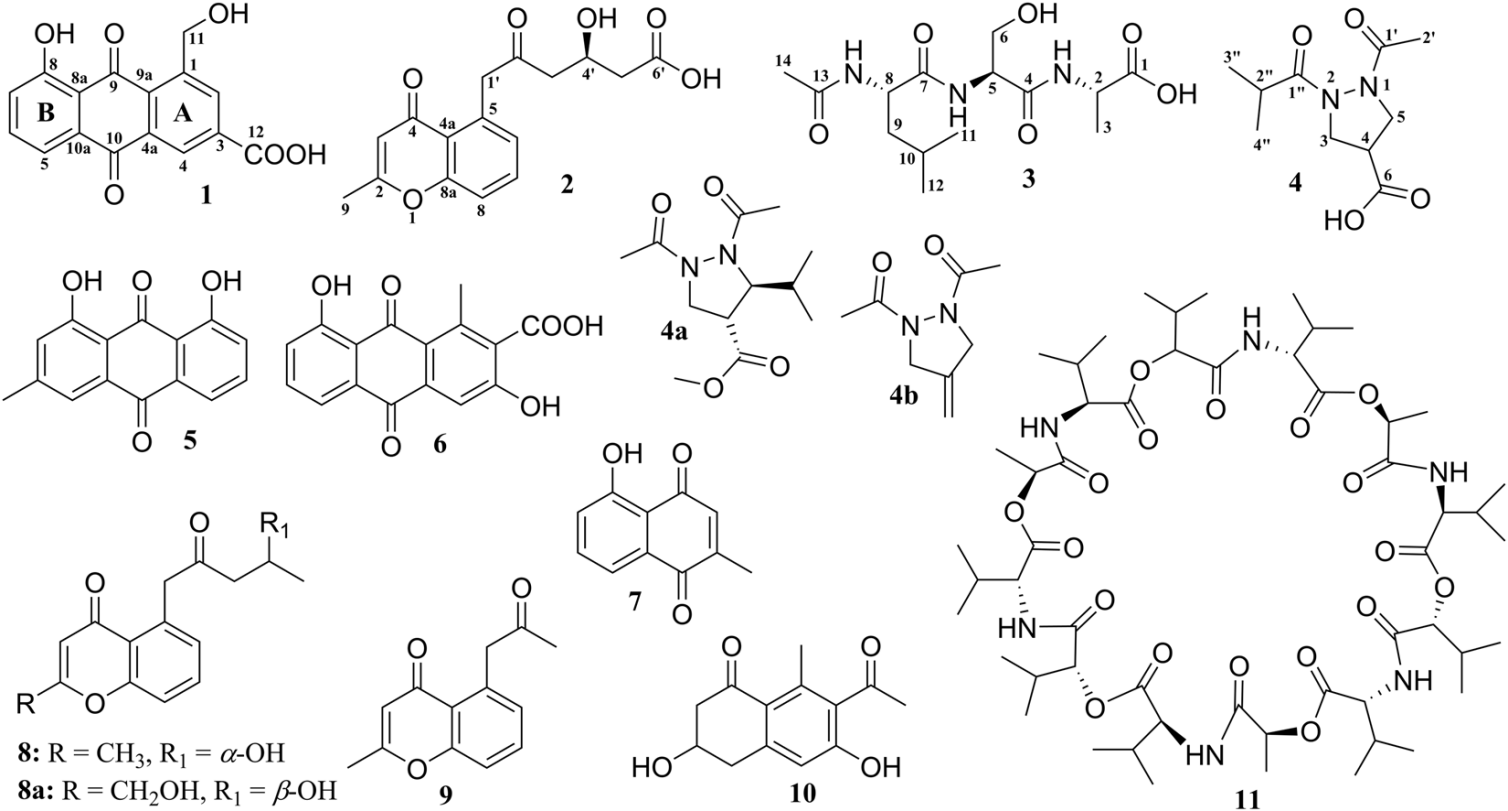
Structures of compounds **1**–**11** isolated from the culture of *Streptomyces* sp. ZZ406.

Actinomycete *Streptomyces* sp. ZZ406 was isolated from sea anemone *Haliplanella lineata*. A MeOH extract prepared from the culture of strain ZZ406 in SC (soluble starch and casein) liquid medium showed activity in suppressing the proliferation of glioma U87 MG cells with an inhibition rate of 62.1% and in reducing the production of lactate by 56.6% in glioma U87MG cells. Chemical investigation of this crude active extract led to the isolation and identification of four new compounds (**1**–**4**) and seven known ones (**5**–**11**) (Fig. 1). New compounds **1** and **2** and known valinomycin (**11**)^27^ showed activity in inhibiting the proliferation of different glioma cells and downregulating the expressions of glioma metabolic regulators. These three active compounds might be responsible for the activity of the crude extract. Herein, we report the isolation and culture of strain ZZ406, the isolation and structural elucidation of new compounds, and their activity against glioma cells.

## Results and Discussion

The strain *Streptomyces* sp. ZZ406 was a sea anemone-associated actinomycete. Its 16S rDNA gene sequence (Fig. S1, supporting information) completely matched (99% identity for a 1390 bp stretch of sequence) those of several *Streptomyces* strains including *S. fulvissimus* DSM 40593, *S. pratensis* ATCC 33331, *S. griseus* subsp. *griseus* NBRC 13350, and *S. halstedii* NRRL ISP-5068 (Table S1). A large culture (70 L) of strain ZZ406 was conducted in the SC liquid medium. The crude extract prepared from the culture was separated by column chromatography, following by HPLC purification to give compounds **1**–**11** (Fig. 1).

The known compounds **5**–**11** were identified as chrysophanol (**5**)^29,30^, 3,8-dihydroxy-1-methyl-anthraquinone-2-carboxylic acid (**6**)^31^, plumbagin (**7**)^32^, phaeochromycin H (**8**)^33^, phaeochromycin G (**9**)^33^, GTRI-02 (**10**)^34^, and valinomycin (**11**)^27^ based on their NMR spectroscopic analysis, [α]_D_ values, and the comparison of the reported data. Phaeochromycin H was initially isolated from *Streptomyces* sp. DSS-18^33^, however, its 4′-OH configuration was not assigned therein. It is known that the stereochemistry at 4′ of **8a** could be determined based on its [α]_D_ value, whereby a positive [α]_D_ value indicated an *R*-configuration, while a negative [α]_D_ value was suggestive of a *S*-configuration^35^. Both **8** from *Streptomyces* sp. ZZ406 and phaeochromycin H from *Streptomyces* sp. DSS-18 were assigned as 4′*R* because they had same positive [α]_D_ value.

**Figure 2:**
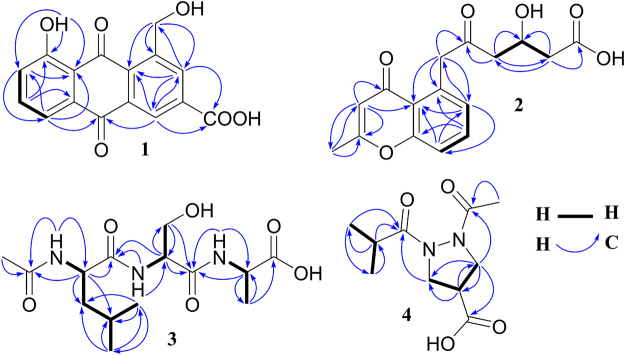
^1^H-^1^H COSY and key HMBC correlations of new compounds **1**–**4**.

Compound **1** has a molecular formula C_16_H_10_O_6_ deduced from its negative HRESIMS [M − H]^−^ and ^13^C-NMR data. Its UV absorption and NMR data suggested that **1** is an anthraquinone derivative^29,30^. The substituted pattern of **1** was suggested by a pair of *meta*-coupled aromatic protons at *δ*_H_ 7.62 (1 H, d, *J* = 2.5 Hz, H-2) and 7.43 (1 H, d, *J* = 2.5 Hz, H-4) for ring A and 3,4,5-trisubstituted aromatic protons at *δ* 7.61 (1 H, d, *J* = 7.8 Hz, H-5), 7.68 (1 H, t, *J* = 8.3, 7.8 Hz, H-6), and 7.30 (1 H, d, *J* = 8.3 Hz, H-7) for ring B. In the HSQC spectrum of **1**, a ^1^H NMR signal at *δ*_H_ 5.02 (2 H) was corrected to a ^13^C NMR signal at *δ*_C_ 62.0, suggesting the presence of an oxymethylene group. In addition, in the ^1^H NMR spectrum of **1**, a low field singlet at *δ*_H_ 12.89 confirmed the presence of a chelated hydroxy group. The ^13^C NMR spectrum of **1** displayed 16 carbon signals, of which 14 were assigned to the anthraquinone backbone, one to the oxymethylene group, and the remaining one to a carboxylic group. As depicted in Fig. 2, HMBC correlations determined the oxymethylene group at C-1 and the carboxylic group at C-3. The ^13^C and ^1^H assignments of **1** was made based on the ^1^H-^1^H COSY, HSQC, and HMBC correlations. The structure of **1** was elucidated as 1-hydroxymethyl-8-hydroxy-anthraquinone-3-carboxylic acid, a new anthraquinone.

**Figure 3:**
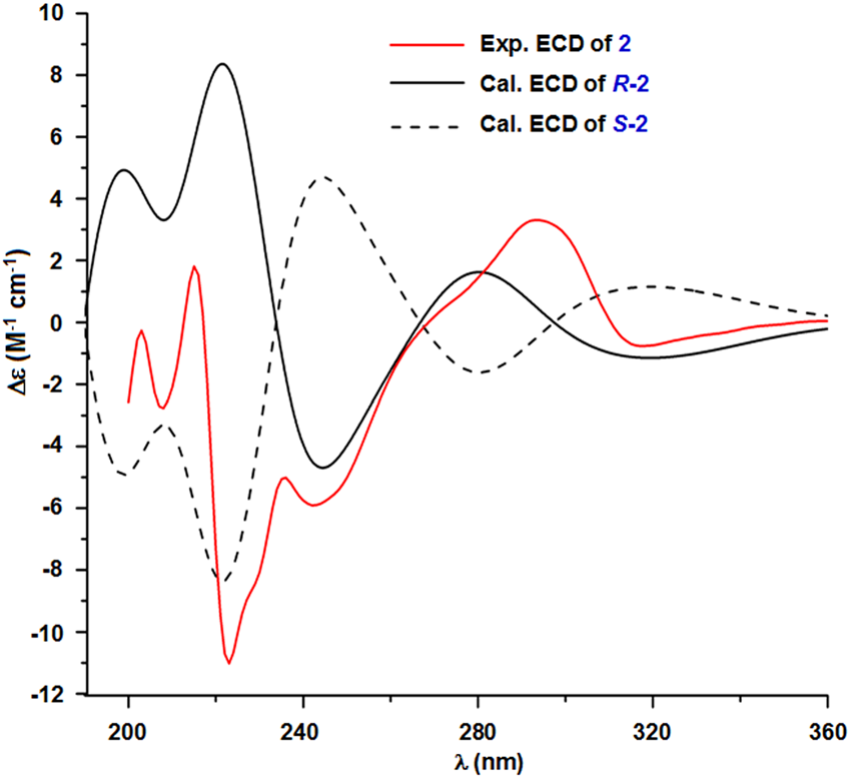
Experimental ECD spectrum (190–360 nm) of compound **2** and calculated ECD spectra of the model molecules of **2** (*R* or *S*) at the B3LYP/6-311++ G(d,p) level in gas phase.

The molecular formula C_16_H_16_O_6_ of **2** was determined according to its positive HRESIMS and ^13^C NMR data. The ^13^C NMR spectrum showed 16 signals for three carbonyls, eight aromatic carbons, one oxymethine, three methylenes, and one methyl. Further HSQC and HMBC spectroscopic analyses indicated that the structural backbone of **2** is 2-methylchromone^33^, which resonated at *δ*_C_ 165.4 (C, C-2), 110.8 (CH, C-3), 178.4 (C, C-4), 121.0 (C, C-4a), 136.6 (C, C-5), 128.9 (CH, C-6), 132.9 (CH, C-7), 117.1 (CH, C-8), 157.2 (C, C-8a), 19.6 (CH_3_, C-9) and *δ*_H_ 6.11 (1 H, s, H-3), 7.12 (1 H, d, *J* = 7.3 Hz, H-6), 7.62 (1 H, dd, *J* = 8.3, 7.3 Hz, H-7), 7.47 (1 H, dd, *J* = 8.3, 1.0 Hz, H-8), 2.34 (3 H, s, H-9). In addition, the ^13^C NMR signals at *δ*_C_ 48.8 (C-1′), 205.7 (C-2′), 50.6 (C-3′), 64.7 (C-4′), 43.3 (C-5′), 175.6 (C-6′) and the ^1^H NMR signals at *δ*_H_ 4.23 (2 H, s, H-1′), 2.60 (2 H, d, *J* = 6.5 Hz, H-3′), 4.10 (1 H, m, H-4′), 1.92 (1 H, dd, *J* = 15.2, 8.6 Hz, H-5a’), 2.13 (1 H, dd, *J* = 15.2, 3.5 Hz, H-5b’) were also observed. These NMR signals were contributed to the side chain of 2′-oxo-4′-hydroxy-hexanoic acid, which was confirmed by HSQC, ^1^H-^1^H COSY, and HMBC correlations (Fig. 2). The absolute configuration of C-4′ was established by theoretical electronic circular dichroism (ECD) calculation. Conformational analyses were carried out by random searching in the Sybyl-X 2.0 using the MMFF94S force field with an energy cutoff of 2.5 kcal/mol^36^. The results showed six lowest energy conformers (**2**-1 C to **2**-6 C, Fig. S60) for *R*-**2** whose relative energy is within 2.5 kcal/mol. Subsequently, the conformers were re-optimized using the DFT method at the B3LYP/6-31 + G(d) level in the gas phase by the GAUSSIAN 09 program (Table S6)^37^. The energies, oscillator strengths, and rotational strengths (velocity) (Tables S7–S12) of the first 60 electronic excitations were calculated using the TDDFT methodology at the B3LYP/6-311++ G(d,p) level in vacuum. The ECD spectra were simulated by the overlapping Gaussian function (half the bandwidth at 1/e peak height, σ = 0.25 eV)^38^. To obtain the final spectra, the simulated spectra of the conformers were averaged according to the Boltzmann distribution theory and their relative Gibbs free energy (ΔG). Through comparison of the calculated ECD spectra with the experimental spectrum (Fig. 3), the absolute configuration at C-4′ of **2** was assigned to be *R*. The ^13^C and ^1^H NMR signals were assigned using ^1^H-^1^H COSY, HSQC, and HMBC correlations (Fig. 2). Based on the foregoing evidence, the structure of **2** was established as 5-(2′-oxo-4′*R*-hydroxy-hexanoic acid)-2-methyl-chromone, named as phaeochromycin I. The planer structure of compound **2** with CAS registry number 1796973-58-4 was recorded in the SciFinder database. However, no NMR data, stereochemistry assignment, physical and chemical properties, or even reference was provided for this synthesized compound. Therefore, compound **2** was designated as a new compound, at least a new natural product.

Compound **3** was obtained as a colorless amorphous powder. Its molecular formula of C_14_H_25_N_3_O_6_ was deduced from HRESIMS [M + Na]^+^ and [M − H]^−^ ions and ^13^C NMR data. The NMR spectra of **3** showed three nitrogenated protons at *δ*_H_ 8.03 (1 H, d, *J* = 8.3 Hz), 7.77 (1 H, d, *J* = 7.7 Hz), 7.60 (1 H, d, *J* = 7.4 Hz) and four carboxyl carbons at *δ*_C_ 175.1, 172.4, 169.4, 169.1, suggesting that **3** was a peptide with at least three amino acids. Further interpretation of the HSQC, ^1^H-^1^H COSY, and HMBC correlations (Fig. 2) demonstrated that compound **3** composed of three amino acids of *N*-acetyl-leucine, serine, and alanine. The sequence of the three amino acids was established to be *N*-acetyl-leucine-serine-alanine based on the HMBC correlations as depicted in Fig. 2. In order to assign the absolute configuration, compound **3** was hydrolyzed by hydrochloric acid to release three free amino acids of l-leucine, l-serine, and l-alanine, which were confirmed by Marfey’s method using standard amino acids as references. The retention times were found to be 7.67 min for l-serine-FDAA, 10.87 min for l-alanine-FDAA, and 17.69 min for l-leucine-FDAA (Fig. S47). Therefore, the structure of **3** was determined as *N*-acetyl-l-leucine-l-serine-l-alanine, a new linear peptide.

**Figure 4:**
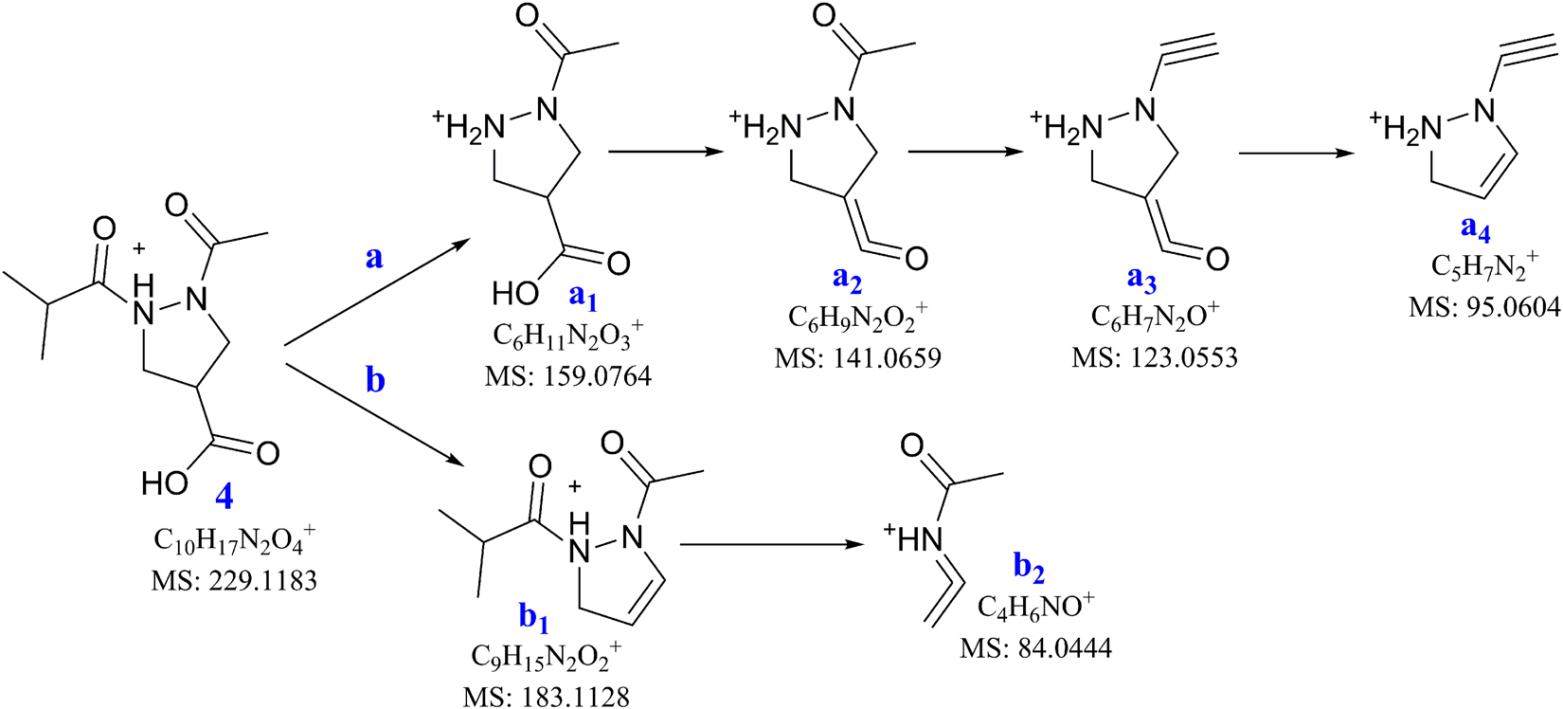
Possible structures for the fragment ions observed in MS-MS spectrum of **4**.

Compound **4** was isolated as a colorless amorphous powder and has a molecular formula of C_10_H_16_N_2_O_4_ deduced from its HRESIMS [M + H]^+^ and [M + Na]^+^ ions and ^13^C NMR data. Analyses of ^1^H, ^13^C, and HSQC NMR spectra indicated that **4** contained three carbonyls, two methines, two methylenes, and three methyls. An acetyl in **4** was **easily** recognized by a HMBC correlation of H-2′ (*δ*_H_ 2.18, 3 H, s) with C-1′ (*δ*_C_ 158.3) and located at N-1 position as H-5 (*δ*_H_ 3.73, 2 H, br s) had a HMBC correlation with C-1′. The presence of a 2-methyl-1-oxopropyl group at N-2 was indicated by its NMR signals at *δ*_C_ 175.3 (C-1″), 33.2 (C-2″), 18.6 (C-3″, C-4″) and *δ*_H_ 2.51 (1 H, m, H-2″), 1.06 (6 H, d, *J* = 7.0 Hz, H-3″, H-4″) as well as HMBC correlations as described in Fig. 2. Similarly, the carboxylic group (*δ*_C_ 168.2, C-6) at C-4 position and the partial structure of -CH_2_-CH-CH_2_- (C-3 to C-5, *δ*_C_ 62.4, 40.3, 57.3; *δ*_H_ 5.42, 3.28, 3.73) were confirmed by HMBC and ^1^H-^1^H COSY correlations as showed in the Fig. 2. MS-MS analysis was also applied to confirm the structure of **4**. A series of fragment ions was observed in the MS-MS spectrum (Fig. S59) and the possible structures for these fragment ions have been proposed (Fig. 4). There are two fragmentation pathways for **4**: (a) bond N(2)–C(1″) can break to give fragment ion at *m/z* 159.0758 (**a**_**1**_, cald. C_6_H_11_N_2_O_3_^+^, 159.0764), which loses H_2_O to afford 141.0653 (**a**_**2**_, cald. C_6_H_9_N_2_O^+^, 141.0659), and **a**_**2**_ can further fracture to fragment ions at *m/z* 123.0549 (**a**_**3**_, cald. C_6_H_7_N_2_O^+^, 123.0553) and 95.0610 (**a**_**4**_, cald. C_5_H_7_N_2_^+^, 95.0604); (b) loss of the carboxyl group at C-4 to give ion at *m/z* 183.1121 (**b**_**1**_, cald. C_9_H_15_N_2_O_2_^+^, 183.1128), which can fracture to fragment ion at *m/z* 84.0452 (**b**_**2**_, cald. C_4_H_6_NO^+^, 84.0444). In order to assign the absolute configuration of C-4, theoretical ECD calculation was conducted for **4**. The results showed that the different orientations of the two substitutes at N-1 and N-2 had significant effects on the calculated ECD spectra of **4**. In the case where the chiral center at C-4 is constant, the calculated ECD spectra of **4** were completely opposite when the two substitutes at N-1 and N-2 positioned at up and down or down and up. Therefore, the configuration of C-4 in **4** was not able to be determined in this study. Based on the above NMR spectroscopic analyses, in combination with the HRESIMS and MS-MS data, the structure of **4** was identified as a new pyrazolidine derivative, 1-acetyl-2-isobutyrylpyrazolidine-4-carboxylic acid. Although pyrazolidine derivatives such as **4a** ^39^ and **4b** ^40^ were previously reported by synthesis, this type of pyrazolidines just like **4** was found from natural resources for the first time.

New compounds **1**–**4** were tested for their activity in inhibiting the proliferation of human glioma U87MG, U251 and SHG44 cells by SRB assay. Doxorubicin (DOX, a chemotherapeutic drug) was used as a positive control. It has been found that **1** had potent activity against different glioma cells with IC_50_ values in a range of 4.7 to 8.1 μM and **2** had activity with IC_50_ values of 21.6–25.8 μM (Table 1). The control DOX had antiproliferative activity with IC_50_ values of 1.9–9.6 μM. Unfortunately, new compounds **3** and **4** were inactive. Because new compound **1** is an anthraquinone with good activity in suppressing the proliferation of glioma cells, its analogues **5** and **6** were also assayed for their activity against glioma. The results showed that both known anthraquinones **5** and **6** also had anti-glioma activity with IC_50_ values of 0.5 to 3.0 μM for **5** and 10.4 to 36.3 μM for **6**. A marine anthraquinone SZ-685C^41^ and herbal anthraquinones aloe emodin^42^ and rhein^43^ were reported to have activity against glioma, suggesting that these simple anthraquinones are sensitive to glioma cells. The cytotoxicity (CC_50_) of the two active compounds **1** and **2** towards normal human astrocytes (HA) was also assayed. The results (Table 1) indicated that **1** had much higher selectivity index (CC_50_/IC_50_, >12.3 to 21.3) than **2** (>3.8 to 4.6) and DOX (0.9 to 4.6).

**Table 1:** Antiproliferative activity of compounds **1** and **2** (mean ± s.d., n = 3).

Compounds	Glioma cells (GC, IC_50_: μM)			Human astrocytes (HA, CC_50_: μM)
	U251	U87MG	SHG44	
**1** Selectivity index (CC_50_/IC_50_)	5.7 ± 0.3 >17.5	4.7 ± 0.2 >21.3	8.1 ± 0.4 >12.3	>100
**2** Selectivity index (CC_50_/IC_50_)	21.6 ± 1.7 >4.6	25.7 ± 2.4 >3.8	25.8 ± 3.1 >3.8	>100
**5**	1.2 ± 0.1	0.5 ± 0.1	3.0 ± 0.2	NT
**6**	13.0 ± 0.6	10.4 ± 0.5	36.3 ± 0.2	NT
DOX Selectivity index (CC_50_/IC_50_)	9.6 ± 1.3 0.9	1.9 ± 0.4 4.6	2.5 ± 1.1 3.5	8.7 ± 1.2

NT: No testing.

**Figure 5:**
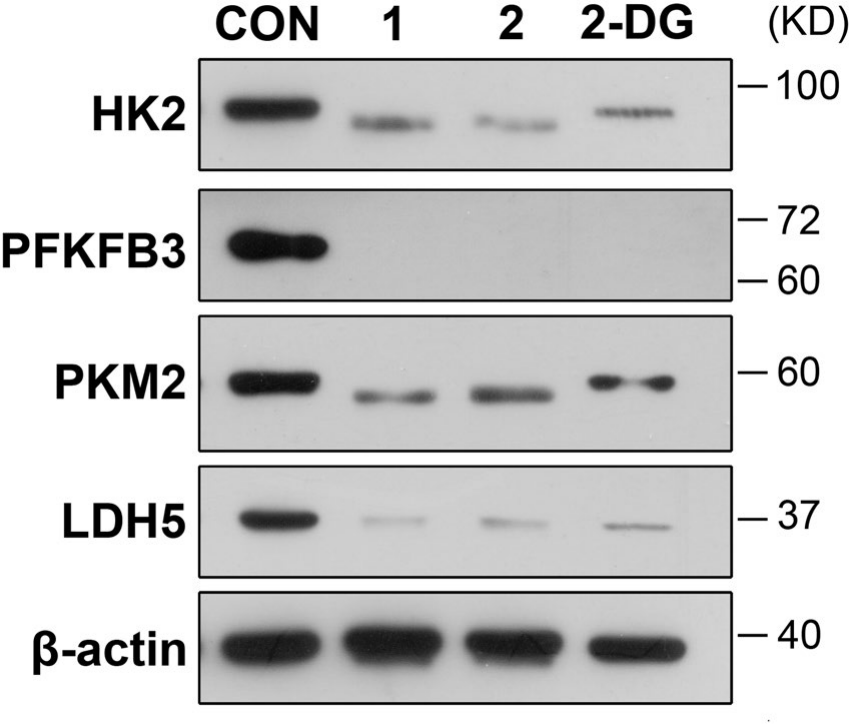
The expression levels of HK2, PFKFB3, PKM2, and LDH5 in glioma U87MG cells. U87-MG cells were treated with compounds **1** (30 μM), **2** (60 μM), or 2-DG (6.0 mM) for 48 h. Protein extracted from the compound-treated cells was subjected to western blot analysis. Compounds **1** and **2** remarkably reduced the expression levels of HK2, PFKFB3, PKM2, and LDH5 in U87MG cells, when compared to the blank control (CON) (HK2: hexokinase 2; PFKFB3: 6-phosphofructo-2-kinase/2,6-bisphosphatase 3; PKM2: pyruvate kinase M2; LDH5: lactate dehydrogenase 5; β-actin: internal control).

The active compounds **1** and **2** were further assayed for their effects on the expression levels of important tumor glycolytic enzymes (regulators) of HK2, PFKFB3, PKM2, and LDH5. Previous study demonstrated that these four tumor metabolic regulators were highly expressed in U87MG cells^27^. Therefore, the effects of **1** and **2** on the expressions of these regulators in U87MG cells were evaluated. 2-Deoxyglucose (2-DG, a hexokinase inhibitor)^8^, was used as a positive control. The U87MG cells were treated by **1** (30.0 μM), **2** (60.0 μM), or 2-DG (6.0 mM) for 48 h. Protein prepared from the compound-treated U87MG cells was subjected to western blot analysis. As shown in Fig. 5, both **1** and **2** clearly reduced the expression levels of HK2, PFKFB3, PKM2, and LDH5. The full-length blots of Fig. 5 were provided as supplementary information (Fig. S61).

**Figure 6:**
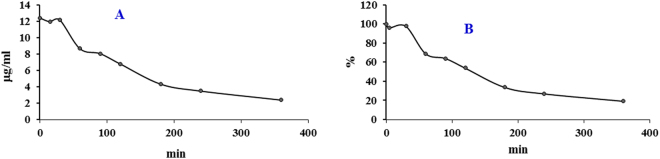
Stability of compound **1** in human liver microsomes incubated at 37 °C. (**A**) The concentration (μg/mL) of **1** in human liver microsomes at the designed time points. (**B**) the percentage (%) of **1** in the designed time points, compared to the zero time point.

Because compound **1** showed potent activity against glioma cells with very high selectivity index and unique anti-glioma mechanism, suggesting **1** might have potential as an anti-glioma agent. Therefore, it is interesting to understand the stability of compound **1** in human liver microsomes. Human liver microsomes were incubated with compound **1** at 37 °C in a water bath and the concentration of **1** at designed time points of 0, 15, 30, 60, 90, 120, 180, 240, and 360 min was determined by HPLC analysis. The results (Table 2 and Fig. 6) indicated the concentration of **1** had no change after the incubation of 30 min. The percentage of **1** was 98.4% (30 min), 70.2% (60 min), 65.3% (90 min), 54.8% (120 min), 35.5% (180 min), 28.2 (240 min), and 19.4% (360 min) when compared to 100.0% (0 min). The data indicated **1** was very stable in human liver microsomes in the first 30 min incubation with concentration gradually decreasing after the incubation of 30 min.

**Table 2:** Stability of compound **1** in human liver microsomes incubated at 37 °C.

Time (min)	0	15	30	60	90	120	180	240	360
Concentration	12.4	12.0	12.2	8.7	8.1	6.8	4.4	3.5	2.4
Percentage (%)	100.0	96.8	98.4	70.2	65.3	54.8	35.5	28.2	19.4

It is well known that many microbial secondary metabolic biosynthetic gene cluster are silent under common cultural conditions and different methods are used to activate the cryptic gene cluster to obtain novel secondary metabolites^44,45^. The easiest and most common method is OSMAC (One Strain, Many Compounds), which uses different cultural media to induce different silent gene cluster to express and then produce different novel bioactive natural products^45^. In this study, ten different media were applied to culture strain ZZ406. A crude extract prepared from the culture of strain ZZ406 in the SC liquid medium showed more secondary metabolites by HPLC analysis and had the strongest activity in suppressing the proliferation of human glioma cells and in reducing the production of lactate. Chemical investigation of this crude active extract led to the isolation and structural elucidation of four new compounds (**1**–**4**) with different structural classes. The results from this study further supported the OSMAC strategy for the discovery of novel microbial natural products.

New compounds **1** showed potent activity in inhibiting the proliferation of human U251, U87MG, and SHG44 glioma cells with less cytotoxicity towards normal HA and good stability in human liver microsomes. Compound **1** also remarkably downregulated the expressions of several important tumor glycolytic enzymes. The data suggested that targeting multiple glioma metabolic enzymes might be one of the anti-glioma mechanisms of **1**. Full understanding of this mechanism of action and the anti-glioma effect of **1** in animal models need to be further explored.

Our previous studies^23,27,28^ proposed that the *in vitro* SRB screening assay combined with the detection of lactate production might be a new way to discover novel anti-glioma compounds from marine actinomycetes with unique mechanism by regulating multiple tumor metabolic enzymes. The data from this study further support this proposal.

## Methods

### General experimental procedures

UV spectra were recorded on a METASH UV-8000 (Shanghai METASH Instruments Co. Ltd., China). Optical rotation and ECD spectra were measured on a JASCO DIP-370 digital polarimeter and a JASCO J715 spectropolarimeter (JASCO, Japan), respectively. NMR spectra were acquired on a Bruker 500 spectrometer using standard pulse programs and acquisition parameters and chemical shifts were expressed in *δ* (ppm). HRESIMS data were obtained from an Agilent 6230 TOF LC/MS spectrometer. Diaion HP-20 (Mitsubishi Chemical, Japan), silica gel (100–200 mesh, Qingdao Marine Chemical Co. Ltd., China), and octadecyl-functionalized silica gel (ODS, Cosmosil 75C_18_ Prep, Nacalai Tesque Inc., Japan) were used for column chromatography. HPLC separation was performed on a CXTH LC-3000 prepared HPLC system (Beijing Chuangxintongheng Science & Technology Co. Ltd., China) using a Fuji-C_18_ CT-30 column (280 × 30 mm, 10 μm) or an Agilent 1260 HPLC system using an Agilent Zorbax SB-C_18_ column (250 × 9.4 mm, 5 μm). All solvents used in this study were purchased from the Sinopharm Chemical Reagent Co. Ltd. (Shanghai, China). Human glioma U251 (XB-0439), U87MG (JDS-2568), and SHG44 (RX-J150) cells were purchased from the Cell Bank of the Chinese Academy of Sciences. Normal human astrocytes (HA, Cat. No. 1800) were obtained from the ScienCell. Lactate assay kit was obtained from the Nanjing Jiancheng Bioengineering Institute (Nanjing, China). Human liver microsomes (LM-R-01M-SUBK) were ordered from the RILD Research Institute for Liver Diseases (Shanghai) Co. Ltd. (China). Doxorubicin (DOX, 98.0%), N*α*-(2,4-dinitro-5-fluorophenyl)-l-alaninamide (FDAA, 99.0%), d-leucine (99%), l-leucine (98%), d-serine (98.5%), l-serine (99%), d-alanine (99%), and l-alanine (98%) were purchased from Sigma-Aldrich. Gause’s-agar were ordered from the Guangdong Huankai Microbial Science and Technology Co. Ltd. (Guangzhou, China). SC liquid medium (soluble starch 10 g, casein 0.3 g, KNO_3_ 2 g, MgSO_4_∙7H_2_O 0.5 g, K_2_HPO_4_ 2 g, CaCO_3_ 0.02 g, FeSO_4_∙7H_2_O 0.01 g, sea water 1.0 L) was made in the authors’ laboratory.

### Marine sea anemone (*Haliplanella lineata*) material

Fresh sea anemone (*H. lineata*) living in the seaside rocks was collected from the Putuo Mountain close to Zhoushan City (Zhejiang, China) in March 2016. A voucher sample (PT-AS201603) was authenticated by one of the authors (M. X. C.) and deposited in the Laboratory of Institute of Marine Biology, Ocean College, Zhoushan campus, Zhejiang University, Zhoushan, China.

### Isolation of strain ZZ406

Fresh sea anemone *H. lineata* (5.0 g) was washed with sterile sea water three times and ground into homogenate as 10^−1^ g/mL suspension in sterile sea water. The 10^−1^ g/mL suspension was diluted to be 10^−2^, 10^−3^, 10^−4^ g/mL stepwisely. Each diluted suspension (200 μL) was covered on the surface of Gauze’s solid medium in petri dish and incubated at 28 °C for five days. The single colony was picked with sterile needles and transferred to a Gauze’s agar plate. After another five days of growth at 28 °C, the single colony (strain ZZ406, Fig. S62) from 10^−2^ g/mL suspension that grew well was transferred onto Gauze’s agar slants, which were stored at 4 °C until use.

### Taxonomic identity of *Streptomyces* sp. ZZ406

The 16S rDNA analysis of strain ZZ406 was performed by Majorbio (Shanghai, China) and its DNA sequence using BLAST (nucleotide sequence comparison) was compared to the GenBank. The 16S rDNA sequence of strain ZZ406 has been deposited in GenBank (accession number: MF563478). The voucher strain of *Streptomyces sp*. ZZ406 was preserved at the Laboratory of Institute of Marine Biology, Ocean College, Zhoushan campus, Zhejiang University, Zhoushan, China.

### Preparation of crude extract for bioactive assay

The procedure of the preparation of crude extract for bioactive assay was described in the previous publication^26^. The crude extract was made to a concentration of 1.0 mg/mL. Sulforhodamine B (SRB) assay was used to evaluate the activity of the crude extract against the proliferation of glioma U87MG cells.

### Lactate measurement

The lactate level in the extracted-treated U87MG cells was measured using a commercial lactate assay kit according to the manufacturer’s protocols. Briefly, 100 μL U87MG cells (3000/well) were treated with 100 μL extract solution (1 mg/mL) for 72 h. The extract-treated U87-MG cells (2.5 μL) in each well of the 96-well plate were taken into the corresponding well of a new 96-well plate. The enzyme working solution (50 μL) and chromogenic agent (10 μL) from the lactate assay kit were added into each well. The reaction mixture was incubated at 37 °C for 10 min, added 100 μL stop buffer to terminate the reaction, and then measured at 550 nm on a microplate reader. The lactate content was calculated based on the regression equation (standard curve). The alternation (100%) of lactate in the extract-treated U87MG cells was compared to that in the U87MG cells by the treatment of DMSO (CON), which was 100%.

### Large culture of strain ZZ406

Strain ZZ406 from the Gauze’s agar slant was refreshed on the plates of Gauze’s agar at 28 °C for six days. The pure colonies of ZZ406 were inoculated in eight Erlenmeyer flasks (500 mL) each containing 250 mL Gauze’s liquid medium. The flask cultures were incubated at 28 °C on a rotary shaker at 180 rpm for five days to produce seed broth. The spore seed broth (5 mL) was inoculated into a Erlenmeyer flask (500 mL), containing 250 mL of SC liquid medium (soluble starch 10 g, casein 0.3 g, KNO_3_ 2 g, MgSO_4_∙7H_2_O 0.5 g, K_2_HPO_4_ 2 g, CaCO_3_ 0.02 g, FeSO_4_∙7H_2_O 0.01 g, sea water 1.0 L, pH 7.2). All flasks were incubated at 28 °C for 13 days on a rotary shaker at 180 rpm. A total of 70 L culture was prepared for this study.

### Extraction and isolation of compounds 1**–**11

The 70 L culture of strain ZZ406 was filtered into filtrate and mycelia. The filtrate was applied to a HP-20 column eluting with water and then 100% MeOH to obtain MeOH fraction. The mycelia were extracted with MeOH three times to give MeOH extract. The mixture of MeOH fraction and MeOH extract was concentrated under the reduced pressure to afford part A. The part A was successively partitioned with EtOAc and n-BuOH to give part B and part C after removal of the organic solvent.

Part B was fractioned by a silica gel column with gradient elution of cyclohexane and EtOAc to give fractions B_1_–B_9_ based on the results of TLC analysis. Fraction B_2_ was separated by preparative HPLC using a CT-30 column (Fuji-C_18_, 280 × 30 mm, 10 μm, mobile phase: MeOH/H_2_O, 82/18; flow rate: 10 mL/min) to give **5** (50 mg, t_R_ 50 min) and **11** (267 mg, t_R_ 90 min); while **1** (9.3 mg, t_R_ 31 min) was obtained from fraction B_4_ by preparative HPLC purification using the same CT-30 column (mobile phase: MeOH/H_2_O, 75/25; flow rate: 10 mL/min). Similarly, by preparative HPLC purification using the same CT-30 column and the same flow rate, compound **8** (2.6 mg, t_R_ 25 min, MeOH/H_2_O: 60/40) was obtained from fraction B_6_, **10** (8.8 mg, t_R_, 40 min, MeOH/H_2_O: 42/58) and **9** (2.7 mg, t_R_ 41 min, MeOH/H_2_O, 42/58) from fraction B_7_. Fraction B_8_ was fractionated by an ODS column with gradient elution (MeOH and H_2_O) to furnish fractions B_8A_–B_8c_. Fraction B_8A_ was further purified by preparative HPLC with the CT-30 column and the same flow rate to give **2** (8.0 mg, t_R_ 35 min, MeOH/H_2_O: 21/79); while **6** (14.8 mg, t_R_ 30 min) and **7** (62.4 mg, t_R_ 56 min) were obtained from fraction B_8b_ by preparative HPLC purification (column: CT-30, mobile phase: MeOH/H_2_O, 43/57; flow rate: 10 mL/min).

Part C was fractioned on a Sephadex LH-20 column eluting with MeOH to give fractions C_1_–C_6_. By HPLC purification using an Agilent Zorbax column (SB-C_18_, 250 × 9.2 mm, 5 μm) and a flow rate of 0.8 mL/min, **3** (11.3 mg, t_R_ 4.6 min, MeOH/H_2_O: 20/80) was obtained from fraction C_2_ and **4** (14.7 mg, t_R_ 5.7 min, MeOH/H_2_O, 25/75) from C_3_.

#### 1-Hydroxymethyl-8-hydroxy-anthraquinone-3-carboxylic acid (**1**)

Yellowish amorphous powder; molecular formula C_16_H_10_O_6_; UV (MeOH) λ_max_ (log ε) 217 (4.32), 279 (4.22), 411 (3.66) nm; ^1^H NMR data (500 MHz, in DMSO-*d*_6_) *δ* 7.62 (1 H, d, *J* = 2.5 Hz, H-2), 7.43 (1 H, d, *J* = 2.5 Hz, H-4), 7.61 (1 H, d, *J* = 7.8 Hz, H-5), 7.68 (1 H, t, *J* = 8.3, 7.8 Hz, H-6), 7.30 (1 H, d, *J* = 8.3 Hz, H-7), 5.01 (2 H, s, H-11), 12.89 (1 H, s, OH-8); ^13^C NMR data (125 MHz, in DMSO-*d*_6_) *δ* 151.4 (C, C-1), 118.6 (CH, C-2), 128.7 (C, C-3), 112.0 (CH, C-4), 136.9 (C, C-4a), 118.4 (CH, C-5), 136.0 (CH, C-6), 124.3 (CH, C-7), 161.4 (C, C-8), 116.4 (C, C-8a), 189.0 (C, C-9), 120.5 (C, C-9a), 182.4 (C, C-10), 132.5 (C, C-10a), 62.0 (CH_2_, C-11), 163.6 (C, C-12); HRESIMS *m/z* [M − H]^−^ 297.0407 (calcd for C_16_H_9_O_6_, 297.0399).

#### Phaeochromycin I (**2**)

Yellowish amorphous powder; molecular formula C_16_H_16_O_6_; [*α*]_D_^26^ −5.6° (*c* 0.1, MeOH); UV (MeOH) λ_max_ (log ε) 224 (4.09), 302 (3.63) nm; ECD (10 μg/mL, MeOH) λ_max_ (Δε) 214 (+1.7), 222 (−11.2), 243 (−5.6), 294 (+3.97) nm; ^1^H NMR data (500 MHz, in DMSO-*d*_6_) *δ* 6.11 (1 H, s, H-3), 7.12 (1 H, d, *J* = 7.3 Hz, H-6), 7.62 (1 H, dd, *J* = 8.3, 7.3 Hz, H-7), 7.47 (1 H, dd, *J* = 8.3, 1.0 Hz, H-8), 2.34 (3 H, s, H-9), 4.23 (2 H, s, H-1′), 2.60 (2 H, d, *J* = 6.5 Hz, H-3′), 4.10 (1 H, m, H-4′), 1.92 (1 H, dd, *J* = 15.2, 8.6 Hz, H-5′a), 2.13 (1 H, dd, *J* = 15.2, 3.5 Hz, H-5′b); ^13^C NMR data (125 MHz, in DMSO-*d*_6_) *δ* 165.4 (C, C-2), 110.8 (CH, C-3), 178.4 (C, C-4), 121.0 (C, C-4a), 136.6 (C, C-5), 128.9 (CH, C-6), 132.9 (CH, C-7), 117.1 (CH, C-8), 157.2 (C, C-8a), 19.6 (CH_3_, C-9), 48.8 (CH_2_, C-1′), 205.7 (C, C-2′), 50.6 (CH_2_, C-3′), 64.7 (CH, C-4′), 43.3 (CH_2_, C-5′), 175.6 (C, C-6′); HRESIMS *m/z* [M + H]^+^ 305.0998 (calcd for C_16_H_17_O_6_, 305.1025), [M + Na]^+^ 327.0823 (calcd for C_16_H_16_NaO_6_, 327.0845), [M − H_2_O + H]^+^ 287.0893 (calcd for C_16_H_15_O_5_, 287.0919).

#### N-Acetyl-l-leucine-l-serine-l-alanine (**3**)

Colorless amorphous powder; molecular formula C_14_H_25_N_3_O_6_; [*α*]_D_^25^ −26.11° (*c* 0.25, MeOH); UV (MeOH) λ_max_ (log ε) 208 (4.37) nm; ^1^H NMR data (500 MHz, in DMSO-*d*_6_) *δ* 3.73 (1 H, m, H-2), 7.60 (1 H, d, *J* = 7.4 Hz, NH-2), 1.14 (3 H, d, *J* = 7.1 Hz, H-3), 4.15 (1 H, m, H-5), 7.77 (1 H, d, *J* = 7.7 Hz, NH-5), 3.38 (1 H, dd, *J* = 10.9, 6.8 Hz, H-6a), 3.54 (1 H, dd, *J* = 10.9, 5.3 Hz, H-6b), 4.29 (1 H, m, H-8), 8.03 (1 H, d, *J* = 8.3 Hz, NH-8), 1.45 (2 H, m, H-9), 1.58 (1 H, m, H-10), 0.83^*a*^ (3 H, d, *J* = 6.6 Hz, H-11), 0.86^*a*^ (3 H, d, *J* = 6.6 Hz, H-12), 1.83 (3 H, s, H-14); ^13^C NMR data (125 MHz, in DMSO-*d*_6_) *δ* 175.1 (C, C-1), 50.2 (CH, C-2), 18.5 (CH_3_, C-3), 169.1 (C, C-4), 55.1 (CH, C-5), 62.1 (CH_2_, C-6), 172.4 (C, C-7), 51.2 (CH, C-8), 40.8 (CH_2_, C-9), 24.3 (CH, C-10), 21.4^*b*^ (CH_3_, C-11), 23.1^*b*^ (CH_3_, C-12), 169.4 (C, C-13), 22.5 (CH_3_, C-14); HRESIMS *m/z* [M + Na]^+^ 354.1634 (C_14_H_25_N_3_NaO_6_, 354.1641), [M + K]^+^ 370.1375 (calcd for C_14_H_25_N_3_KO_6_, 370.1380), [M − H]^−^ 330.1661 (C_14_H_24_N_3_O_6_, 330.1665). Note, ^*a,b*^the NMR data with the same labels may be interchanged.

#### 1-Acetyl-2-isobutyrylpyrazolidine-4-carboxylic acid (**4**)

Colorless amorphous powder; molecular formula C_10_H_16_N_2_O_4_; [*α*]_D_^25^ + 23.81° (*c* 0.25, MeOH); UV (MeOH) λ_max_ (log ε) 213 (4.05) nm; ECD (10 μg/mL, MeOH) λ_max_ (Δε) 215 (+136.1) nm; ^1^H NMR data (500 MHz, in DMSO-*d*_6_) *δ* 5.42 (2 H, br q, H-3), 3.28 (1 H, m, H-4), 3.73 (2 H, br s, H-5), 2.18 (3 H, s, H-2′), 2.51 (1 H, m, H-2″), 1.06 (6 H, d, *J* = 7.0 Hz, H-3″, H-4″); ^13^C NMR data (125 MHz, in DMSO-*d*_6_) *δ* 62.4 (CH_2_, C-3), 40.3 (CH, C-4), 57.3 (CH_2_, C-5), 168.2 (C, C-6), 158.3 (C, C-1′), 17.7 (CH_3_, C-2′), 175.3 (C, C-1″), 33.2 (CH, C-2″), 18.6 (CH_3_, C-3″, C-4″); HRESIMS *m/z* [M + H]^+^ 229.1183 (calcd for C_10_H_17_N_2_O_4_, 229.1188), [M + Na]^+^ 251.1005 (calcd for C_10_H_16_N_2_NaO_4_, 251.1008).

### Acid hydrolysis of compound 3

Compound **3** (3.0 mg) was dissolved in 1.2 mL 6 N HCl and heated at 110 °C in a 25 mL round flask for 24 h. The hydrolysate was concentrated *in vacuo* and then used for Marfey’s analysis.

### Marfey’s analysis

The hydrolysate was dissolved in 120 μL water and then NaHCO_3_ (1 M, 20 μL) and 1% FDAA in acetone (400 μL) were added to the solution of the hydrolysate. The mixture was stirred at 43 °C for 2 h and the reaction was terminated by an addition of 20 μL of 1 N HCl. The reaction mixture was diluted with 500 μL acetone to give amino acid-FDAA derivatives for HPLC analysis. Standard amino acids (each 1 mg) of l-serine, d-serine, l-leucine, d-leucine, l-alanine, and d-alanine were also converted to corresponding FDAA derivatives. Each of the amino acid-FDAA derivatives (0.8 μL) was analyzed by HPLC (Agilent SB-C_18_ column: 250 × 4.6 mm, 5 μm; flow rate: 1.0 mL/min; detection wavelength: 340 nm). Water containing 0.1% HOAc was employed as mobile phases A and 100% acetonitrile as phase B. The binary gradient program was 0.00–30.00 min with 20–70% B, 30.01–36.00 min with 100% B, and 36.01–43.00 min with 20% B. The amino acid-FDAA derivatives prepared from the hydrolysate of **3** were found to be l-serine-FDAA (t_R_ 7.71 min), l-alanine-FDAA (t_R_ 10.84 min), and l-leucine-FDAA (t_R_ 17.79 min) by comparison with the retention times of authentic l-serine-FDAA (t_R_ 7.67 min), d-serine-FDAA (t_R_ 8.28 min), l-alanine-FDAA (t_R_ 10.87 min), d-alanine-FDAA (t_R_ 12.98 min), l-leucine-FDAA (t_R_ 17.69 min), and d-leucine-FDAA (t_R_ 20.57 min).

### Antiproliferative activity assay

Sulforhodamine B (SRB) assay as described in previous studies^19,20^ was used to determine the activity of tested compounds inhibiting the proliferation of glioma U87MG, U251, SHG44 cells, and normal human astrocytes (HA). Doxorubicin (DOX) was used as a positive control (CON).

### Western blot analysis

Western blot analysis was used to detect the expression levels of tumor glycolytic regulators HK2, PFKFB3, PKM2, and LDH5. The detailed procedure, including protein sample preparation, determination of protein concentration, and western blot analysis, was described in the previous publication^21^.

### Determination of the stability of compound 1 in human liver microsomes

Compound **1** was dissolved in 100 mM NaPO_4_ solution (pH 7.4) to make a sample solution with a final concentration of 125 μg/mL. Human liver microsomes were diluted by 100 mM NaPO_4_ solution (pH 7.4) to make liver microsomal solution with a concentration of 1.25 mg/mL. A mixture of the sample solution (90 μL) and the liver microsomal solution (720 μL) was mixed well and then pre-incubated at 37 °C for 7 min. The pre-incubated mixture was initiated by adding 90 μL 10 mM NADPH solution, mixed well, and then incubated at 37 °C in a water bath. At each designed time point of 0, 15, 30, 60, 90, 120, 180, 240 and 360 min, aliquot 80 μL of the incubated mixture was pipette by micropipet and then quenched by the addition of 320 μL methanol. The reaction mixture was centrifuged at 12000 rpm for 10 min at 5 °C to give the supernatant, which was submitted to HPLC analysis.

## Electronic supplementary material

Supplementary Information
