# Supplementary material for: Anti-glioma Natural Products Downregulating Tumor Glycolytic Enzymes from Marine Actinomycete Streptomyces sp. ZZ406
Source: Sci Rep. 2018 Jan 8;8:72. doi: 10.1038/s41598-017-18484-7 (PMC5758648; doi:10.1038/s41598-017-18484-7)

***Supplementary Information for***

**Anti-glioma Natural Products Downregulating Tumor Glycolytic Enzymes from Marine Actinomycete *Streptomyces* sp. ZZ406**

Mengxuan Chen^1^, Weiyun Chai^1^, Tengfei Song^1^, Mingzhu Ma^1^, Xiao-Yuan Lian^2^,*, Zhizhen Zhang^1^,*

^1^ Ocean College, Zhoushan Campus, Zhejiang University, Zhoushan 316021, China,

^2^ College of Pharmaceutical Sciences, Zhejiang University, Hangzhou 310058, China

*****Correspondence and requests for materials should be addressed to X.-Y. L (email: [xylian@zju.edu.cn](mailto:xylian@zju.edu.cn)) or Z. Z. (email: zzhang88@zju.edu.cn)

**Content**

Figure S_1_. 16S rDNA sequence of *Streptomyces* sp. ZZ406…………………………....….….page 4

Table S_1_. Sequences producing significant alignments……………….….………….….……..page 4

Table S_2_. ^13^C and ^1^H NMR data for compound **1**……………………………...………….…..page 5

Figures S_2_-S_3_. ^1^H-NMR spectra of compound **1**………...………………………..……….…..page 5

Figures S_4_-S_6_. ^13^C-NMR spectra of compound **1**………………...……………..……………..page 6

Figure S_7_. ^1^H-^1^H COSY spectrum of compound **1**………………………………..…………...page 8

Figures S_8_-S_11_. HMBC spectra of compound **1**………………………………..…………..…..page 8

Figure S_12_. HRESIMS of compound **1**……………….………………...……………...……..page 10

Table S_3_. ^13^C and ^1^H NMR data for compound **2**…………………..………...………….…..page 11

Figures S_13_-S_15_. ^1^H-NMR spectra of compound **2**……………………….....…………….…..page 11

Figures S_16_-S_19_. ^13^C-NMR spectra of compound **2**…………………...…………………..…..page 13

Figures S_20_-S_21_. ^1^H-^1^H COSY spectra of compound **2**…………………………………...…..page 15

Figures S_22_-S_24_. HSQC spectra of compound **2**…………………………………….….……..page 16

Figures S_25_-S_27_. HMBC spectra of compound **2**…………………..………………..………..page 17

Figure S_28_. HRESIMS of compound **2**……………..………….……………………………..page 19

Table S_4_. ^13^C and ^1^H NMR data for compound **3**……………………..…………..…...……..page 19

Figures S_29_-S_32_. ^1^H-NMR spectra of compound **3**………………….………………….……..page 20

Figures S_33_-S_36_. ^13^C-NMR spectra of compound **3**………………..…………………...……..page 22

Figures S_37_-S_38_. HSQC spectra of compound **3**…………………………...………………....page 24

Figures S_39_-S_41_. ^1^H-^1^H COSY spectra of compound **3**………………..………………….…..page 25

Figures S_42_-S_45_. HMBC spectra of compound **3**…………..………..……………....………..page 26

Figure S_46_. HRESIMS of compound **3**……………..……………………….………………..page 28

Figure S_47_. HPLC profile of amino acid-FDAAs of hydrolysates of compound **3**.……….…page 29

Figure S_48_. HPLC profile of amino acid-FDAAs of standard amino acids……….……….…page 29

Table S_5_. ^13^C and ^1^H NMR data for compound **4**………………………………….….....…..page 30

Figure S_49_. ^1^H-NMR spectrum of compound **4**…………………………..…………………..page 30

Figures S_50_-S_51_. ^13^C-NMR spectra of compound **4**……………………..………………..…...page 31

Figure S_52_. HSQC spectrum of compound **4**………………………….…..………….……....page 32

Figures S_53_-S_54_. ^1^H-^1^H COSY spectra of compound **4**………………….…...……….……....page 32

Figures S_55_-S_57_. HMBC spectra of compound **4**…………………...…..………………...…..page 33

Figure S_58_. HRESIMS of compound **4**……………... …………………..……….…………..page 35

Figure S_59_. MS-MS spectrum of compound **4**……….………………..………….…………..page 35

Figure S_60_. The optimized conformers and equilibrium populations of compound **2**…….….page 36

Table S_6_. Important thermodynamic parameters (a.u.) of the optimized **2** at the B3LYP/6-

31G(d) level in the gas phase……………………………………………………....page 36

Table S_7_. Excitation energies, oscillator strengths and rotatory strengths in the ECD spectra

of **2**-1C………....…………………………………………………………………..page 37

Table S_8_ Excitation energies, oscillator strengths and rotatory strengths in the ECD spectra

of **2**-2C…………………………………………………………………..…..……..page 38

Table S_9_. Excitation energies, oscillator strengths and rotatory strengths in the ECD spectra

of **2**-3C……………………………………………………………………...……..page 39

Table S_10_. Excitation energies, oscillator strengths and rotatory strengths in the ECD spectra

of **2**-4C……………………………………………………..…………………..…..page 40

Table S_11_. Excitation energies, oscillator strengths and rotatory strengths in the ECD spectra

of **2**-5C……………………………………………………..……………………....page 41

Table S_12_. Excitation energies, oscillator strengths and rotatory strengths in the ECD spectra

of **2**-6C……………………………………………………..…………………..…..page 42

Figure S_61_. Full-length blots of Figure 5………………………………………..……………page 43

Figure S_62_. The colony of marine actinomycete *Streptomyces* sp. ZZ406….................…..…page 43

Figure S_1_. 16S rDNA sequence of *Streptomyces* sp. ZZ406

tgcagtcgacgatgaagccgcttcggtggtggattagtggcgaacgggtgagtaacacgtgggcaatctgcccttcactctgggacaagccctggaaacggggtctaataccggataatactcctgcctgcatgggtgggggttgaaagctccggcggtgaaggatgagcccgcggcctatcagcttgttggtggggtaatggcctaccaaggcgacgacgggtagccggcctgagagggcgaccggccacactgggactgagacacggcccagactcctacgggaggcagcagtggggaatattgcacaatgggcgaaagcctgatgcagcgacgccgcgtgagggatgacggccttcgggttgtaaacctctttcagcagggaagaagcgcaagtgacggtacctgcagaagaagcgccggctaactacgtgccagcagccgcggtaatacgtagggcgcaagcgttgtccggaattattgggcgtaaagagctcgtaggcggcttgtcacgtcggatgtgaaagcccggggcttaaccccgggtctgcattcgatacgggctagctagagtgtggtaggggagatcggaaattcctggtgtagcggtgaaatgcgcagatatcaggaggaacaccggtggcgaaggcggatctctgggccattactgacgctgaggagcgaaagcgtggggagcgaacaggattagataccctggtagtccacgccgtaaacgttgggaactaggtgttggcgacattccacgtcgtcggtgccgcagctaacgcattaagttccccgcctggggagtacggccgcaaggctaaaactcaaaggaattgacgggggcccgcacaagcagcggagcatgtggcttaattcgacgcaacgcgaagaaccttaccaaggcttgacatataccggaaagcatcagagatggtgccccccttgtggtcggtatacaggtggtgcatggctgtcgtcagctcgtgtcgtgagatgttgggttaagtcccgcaacgagcgcaacccttgttctgtgttgccagcatgcctttcggggtgatggggactcacaggagactgccggggtcaactcggaggaaggtggggacgacgtcaagtcatcatgccccttatgtcttgggctgcacacgtgctacaatggccggtacaatgagctgcgatgccgtgaggcggagcgaatctcaaaaagccggtctcagttcggattggggtctgcaactcgaccccatgaagtcggagttgctagtaatcgcagatcagcattgctgcggtgaatacgttcccgggccttgtacacaccgcccgtcacgtcacgaaagtcggtaacacccgaagccggtggcccaaccccttgtgggaggga

Table S_1_. Sequences producing significant alignments

| Accession | Description | Max score | Total score | Query coverage | Evalue | Ident |
| --- | --- | --- | --- | --- | --- | --- |
| NC 021177.1 | *Streptomyces fulvissimus* DSM 40593, complete genome | 2483 | 14898 | 100% | 0.0 | 99% |
| NC 016114.1 | *Streptomyces pratensis* ATCC 33331, complete genome | 2483 | 14864 | 100% | 0.0 | 99% |
| NC 010572.1 | *Streptomyces griseus* subsp. *griseus* NBRC 13350 DNA, complete genome | 2471 | 14831 | 100% | 0.0 | 99% |
| NZ JOAZ  01000047.1 | *Streptomyces halstedii* strain NRRL ISP-5068 contiq47.1, whole genome shotgun sequence | 2460 | 2460 | 100% | 0.0 | 100% |

**Table S_2_. ^13^C and ^1^H NMR data for compound 1**

| No. | C | H (*J* = Hz) | No. | C | H (*J* = Hz) |
| --- | --- | --- | --- | --- | --- |
| 1 | 151.4, C | − | 8-OH | − | 12.89, H, s |
| 2 | 118.6, CH | 7.62, 1H, d (2.5) | 8a | 116.4, C | − |
| 3 | 128.7, C | − | 9 | 189.0, C | − |
| 4 | 112.0, CH | 7.43, 1H, d (2.5) | 9a | 120.5, C | − |
| 4a | 136.9, C | − | 10 | 182.4, C | − |
| 5 | 118.4, CH | 7.61, 1H, d (7.8) | 10a | 132.5, C | − |
| 6 | 136.0, CH | 7.68, H, t (8.3, 7.8) | 11 | 62.0, CH_2_ | 5.01, 2H |
| 7 | 124.3, CH | 7.30, 1H, d (8.3) | 12 | 163.6, C | − |
| 8 | 161.4 | − |  |  |  |

Figure S_2_. ^1^H-NMR spectrum of compound **1**

**
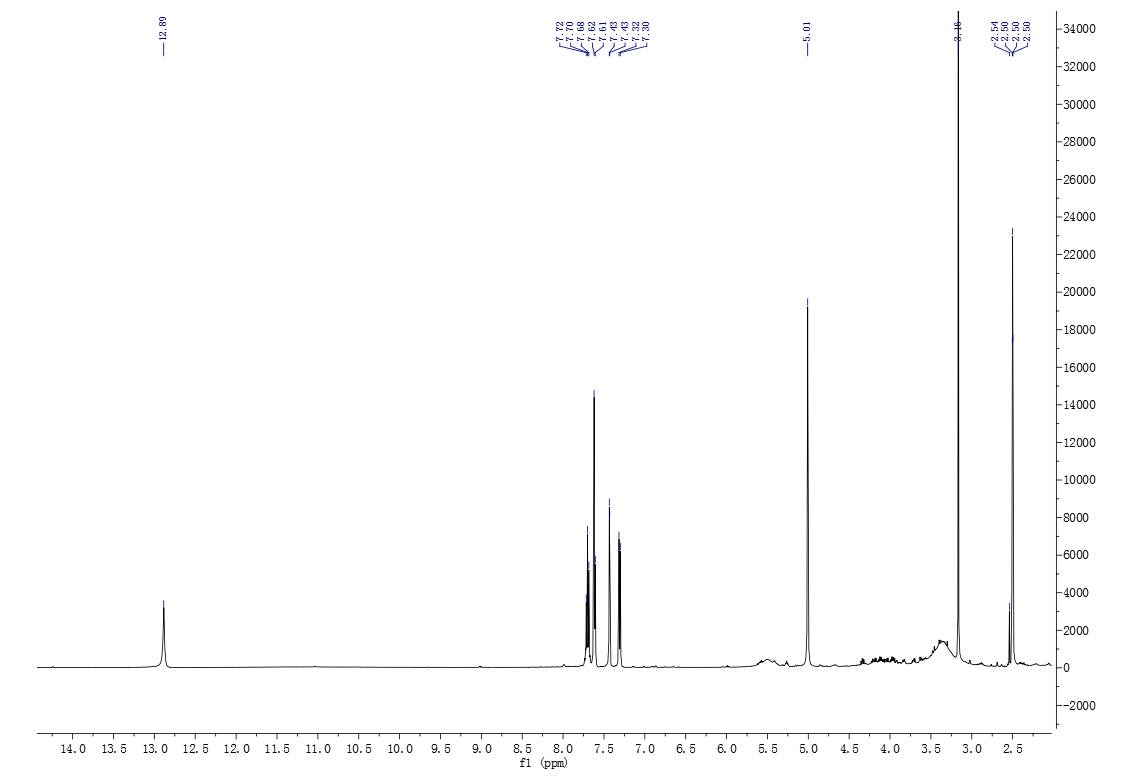
**

Figure S_3_. ^1^H-NMR spectrum of compound **1**

**
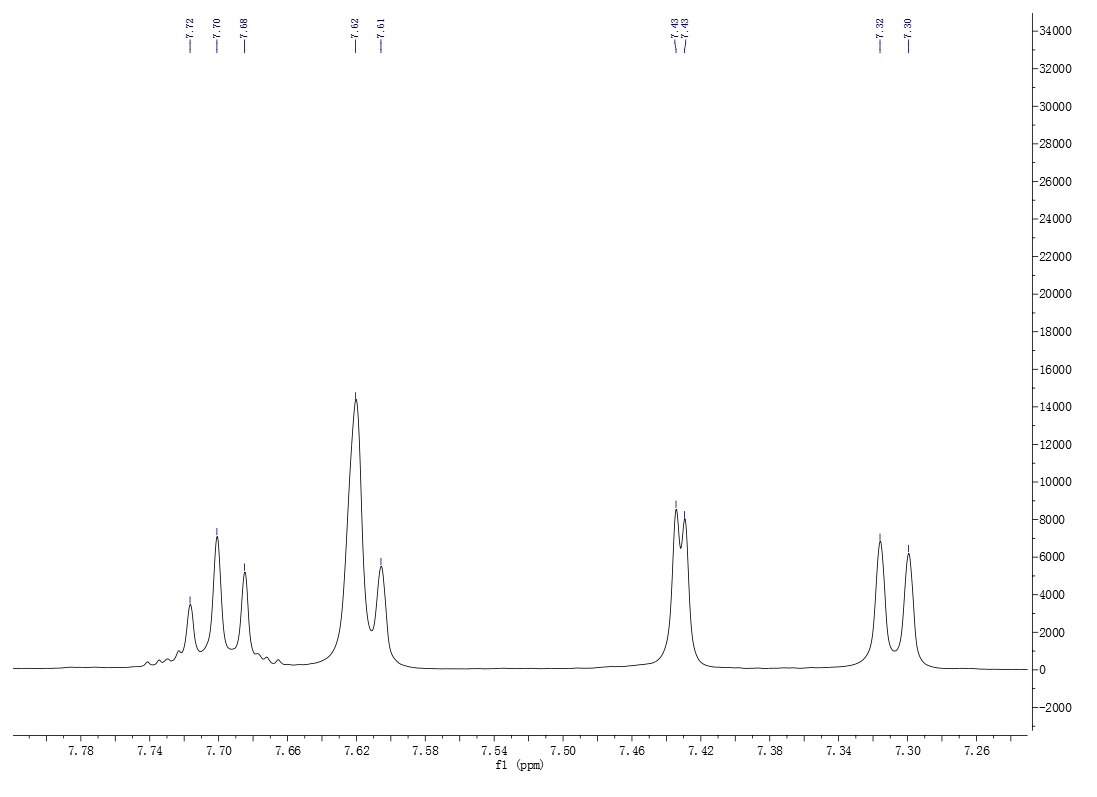
**

Figure S_4_. ^13^C-NMR spectrum of compound **1**

**
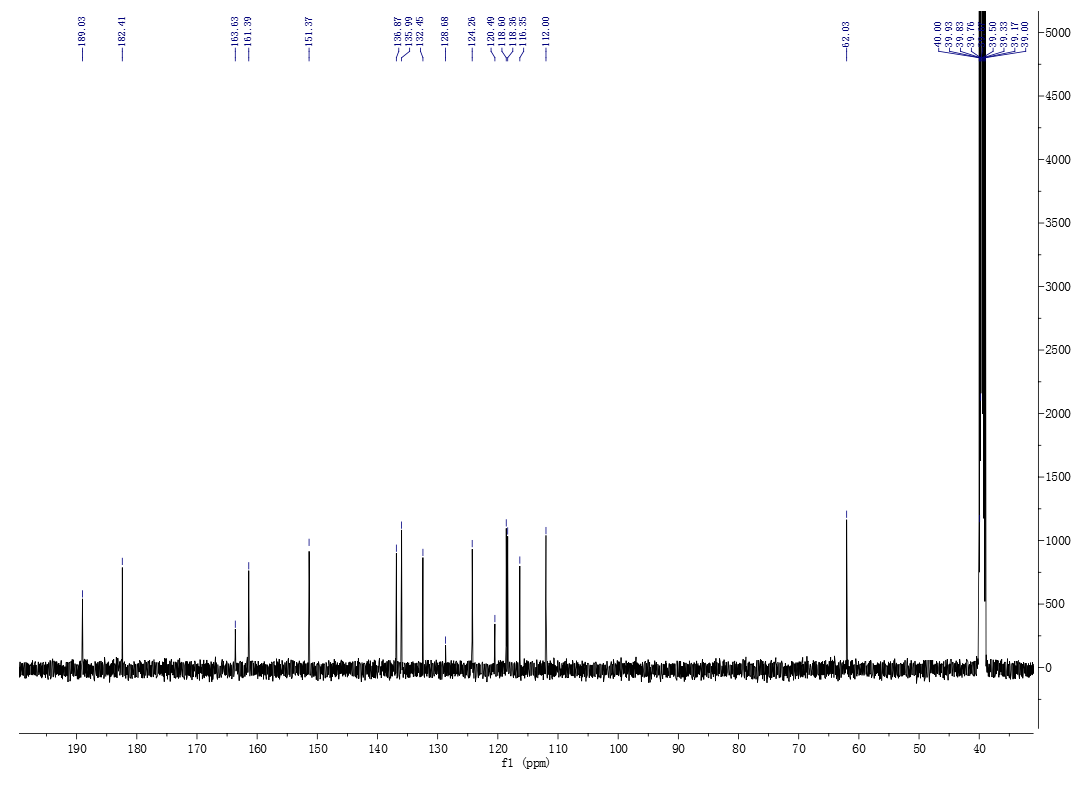
**

Figure S_5_. ^13^C-NMR spectrum of compound **1**

**
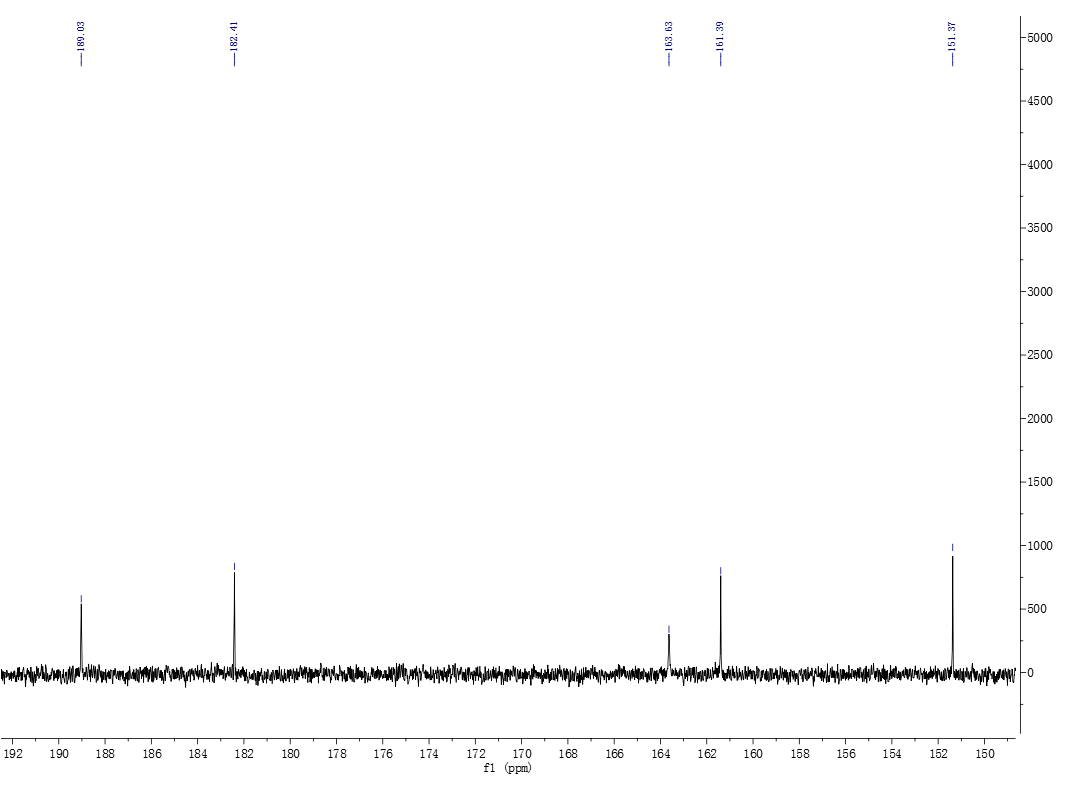
**

Figure S_6_. ^13^C-NMR spectrum of compound **1**

**
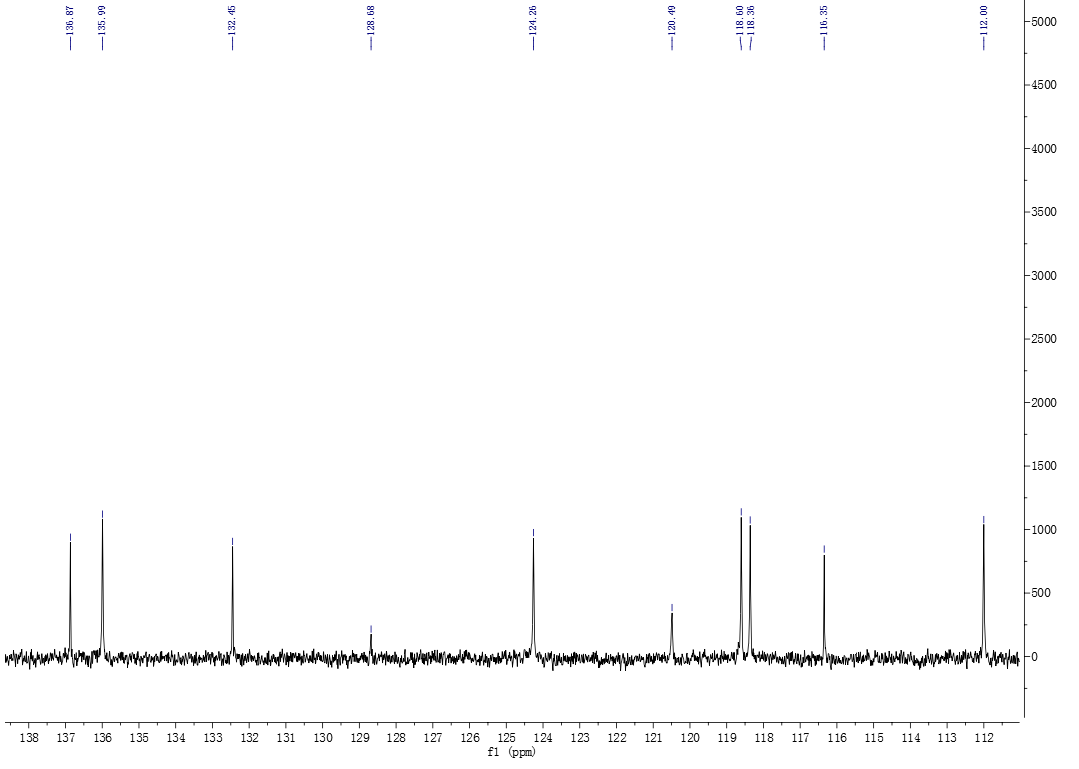
**

Figure S_7_. ^1^H-^1^H COSY spectrum of compound **1**

**
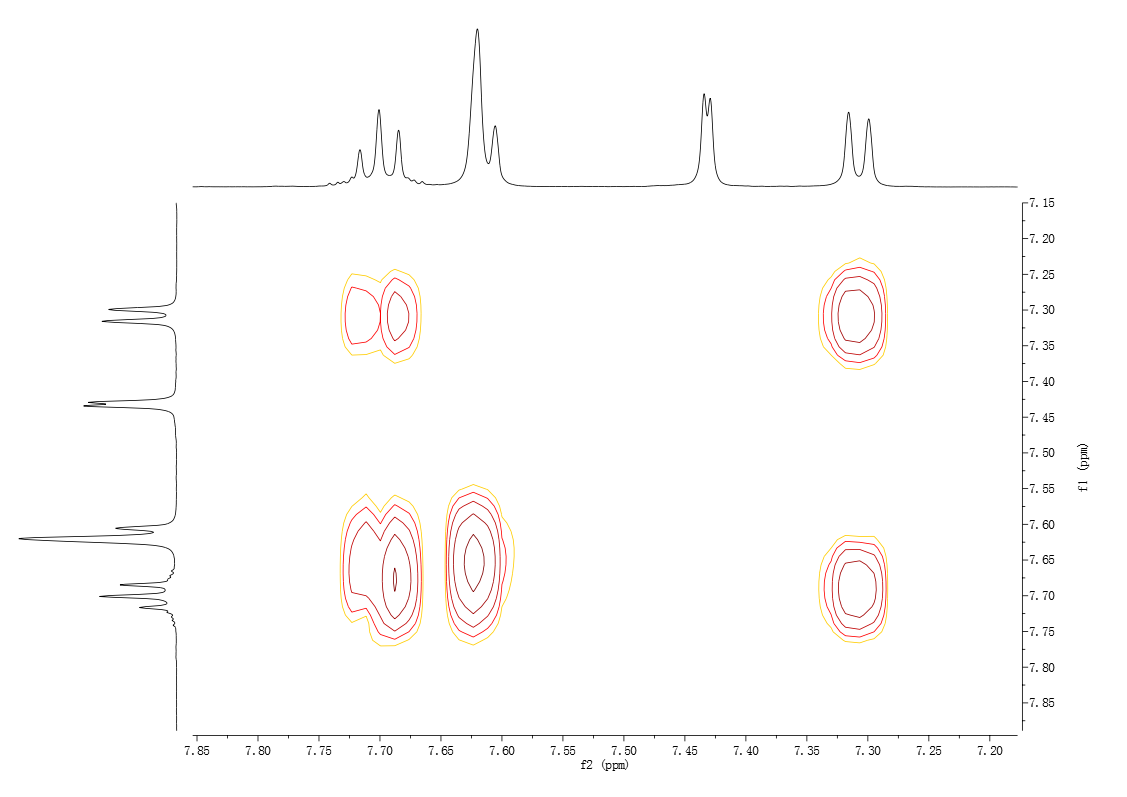
**

Figure S_8_. HMBC spectrum of compound **1**

**
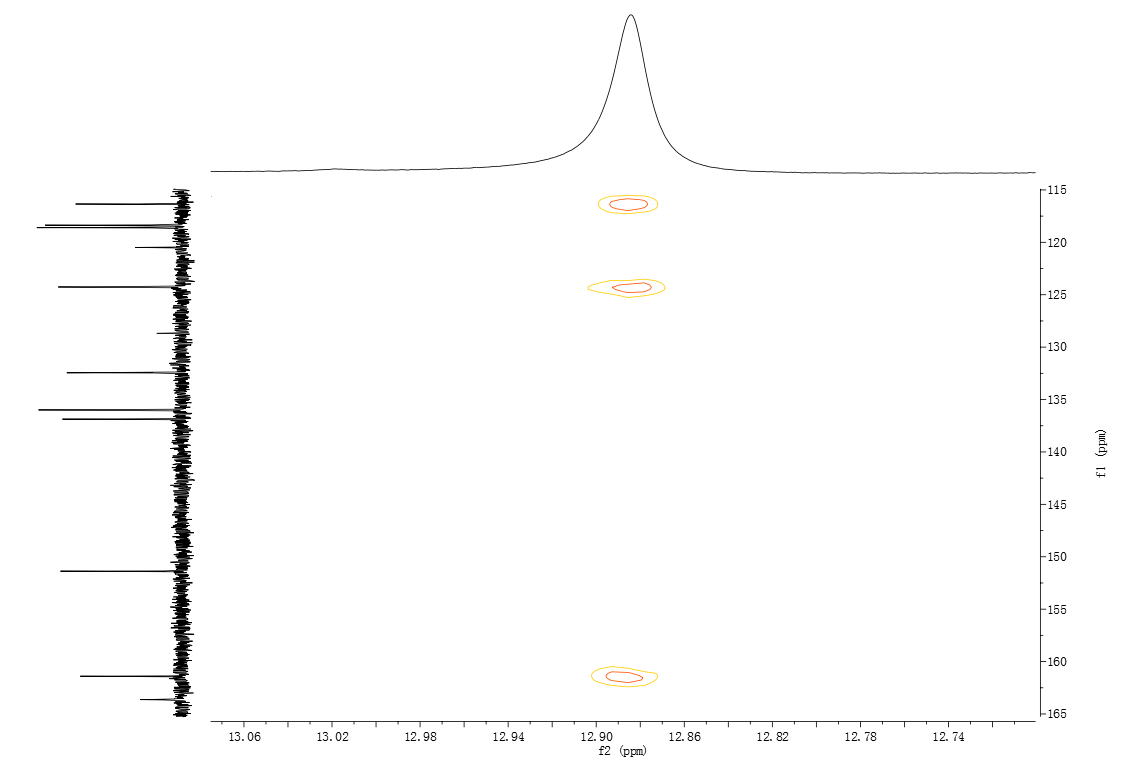
**

Figure S_9_. HMBC spectrum of compound **1**

**
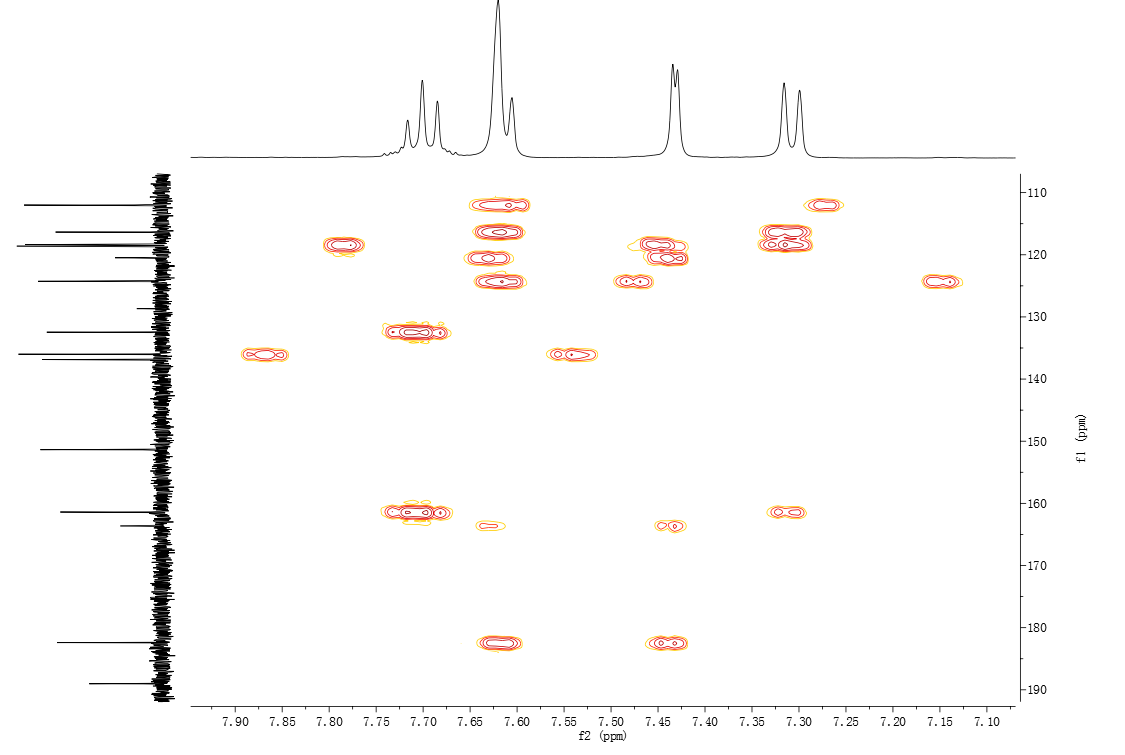
**

Figure S_10_. HMBC spectrum of compound **1**

**
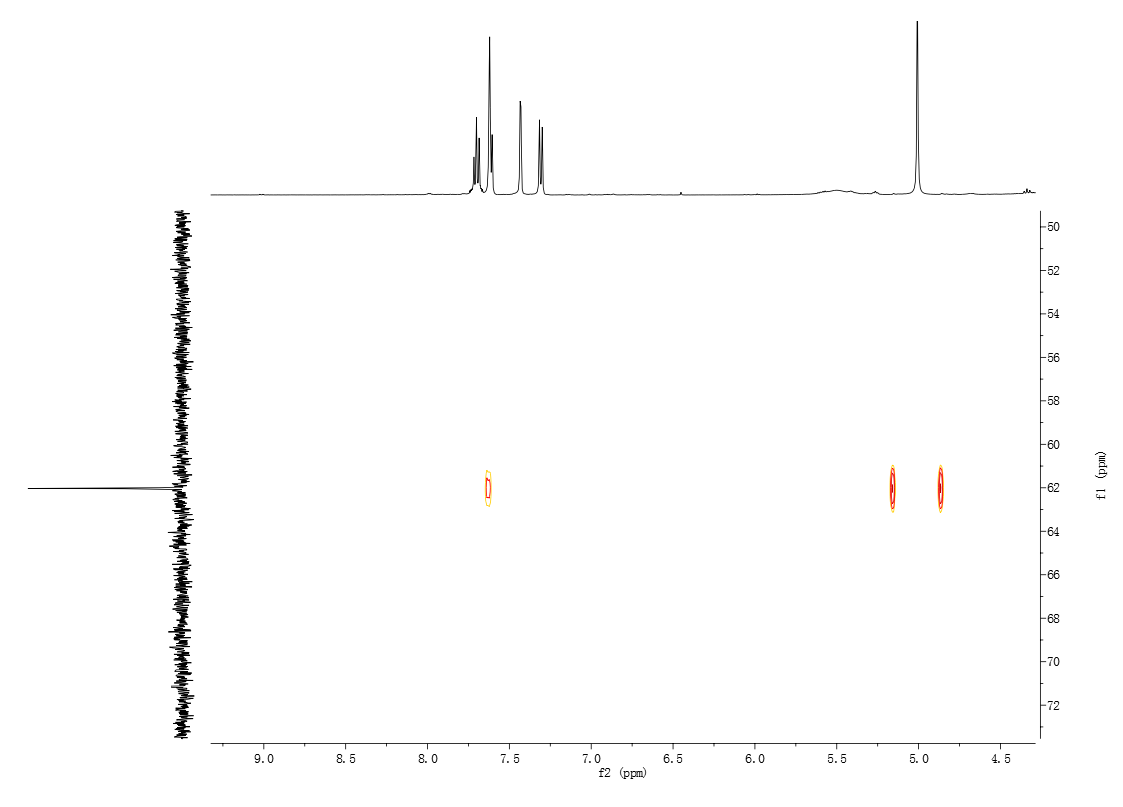
**

Figure S_11_. HMBC spectrum of compound **1**

**
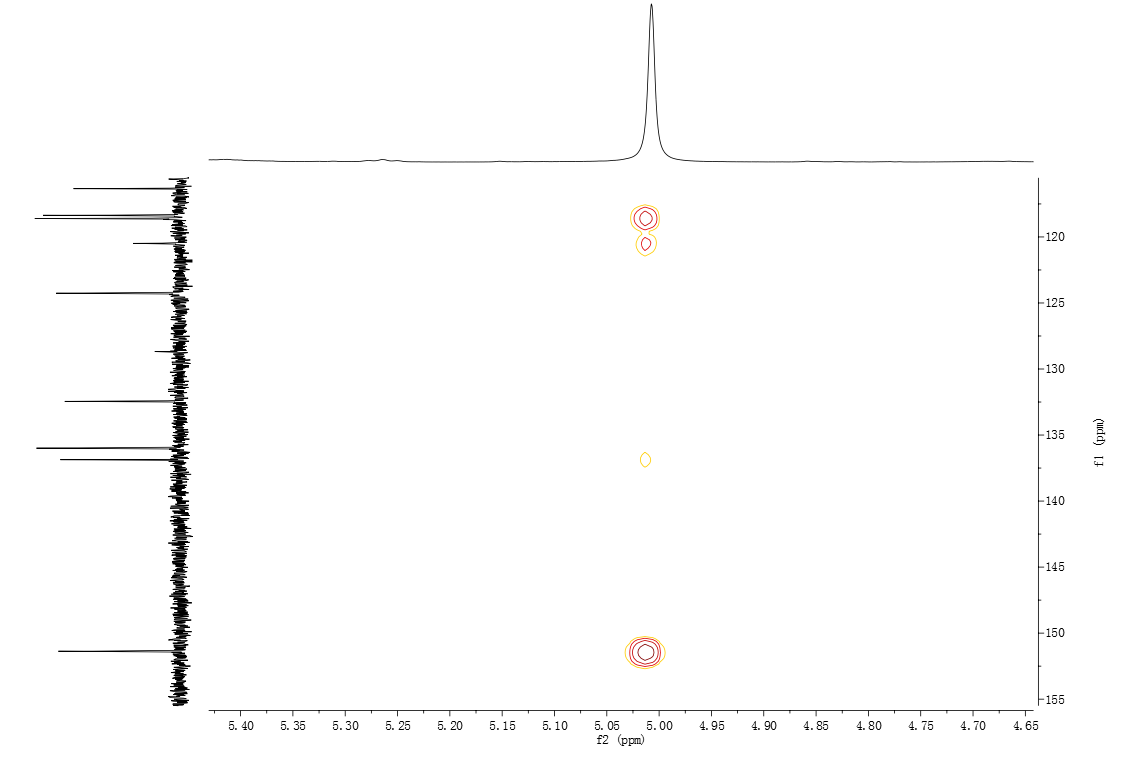
**

Figure S_12_. HRESIMS of compound **1**

**
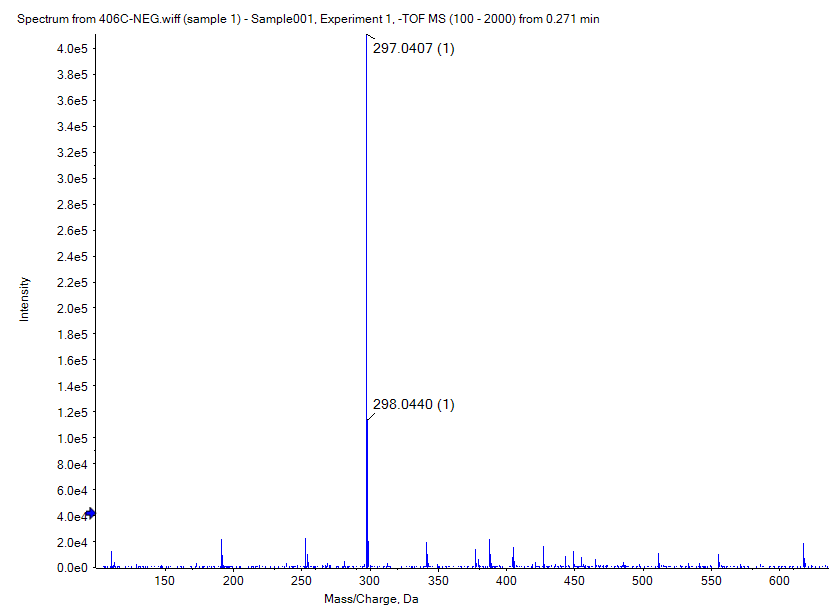
**

**Table S_3_. ^13^C and ^1^H NMR data for compound** 2

| No. | C | H (*J* = Hz) | No. | C | H (*J* = Hz) |
| --- | --- | --- | --- | --- | --- |
| 2 | 165.4, C | − | 8a | 157.2, C | − |
| 3 | 110.8, CH | 6.11, 1H, s | 9 | 19.6, CH_3_ | 2.34, 3H, s |
| 4 | 178.4, C | − | 1′ | 48.8, CH_2_ | 4.23, 2H, s |
| 4a | 121.0, C | − | 2′ | 205.7, C | − |
| 5 | 136.6, C | − | 3′ | 50.6, CH_2_ | 2.60, 2H, d (6.5) |
| 6 | 128.9, CH | 7.12, 1H, d (7.3) | 4′ | 64.7, CH | 4.10, 1H, m |
| 7 | 132.9, CH | 7.62, 1H, dd (8.3, 7.3) | 5′ | 43.3, CH_2_ | 1.92, 1H, dd (15.2, 8.6);  2.13, 1H, dd (15.2, 3.5); |
| 8 | 117.1, CH | 7.47, 1H, dd (8.3, 1.0) | 6′ | 175.6, C | − |

Figure S_13_. ^1^H-NMR spectrum of compound **2**

**
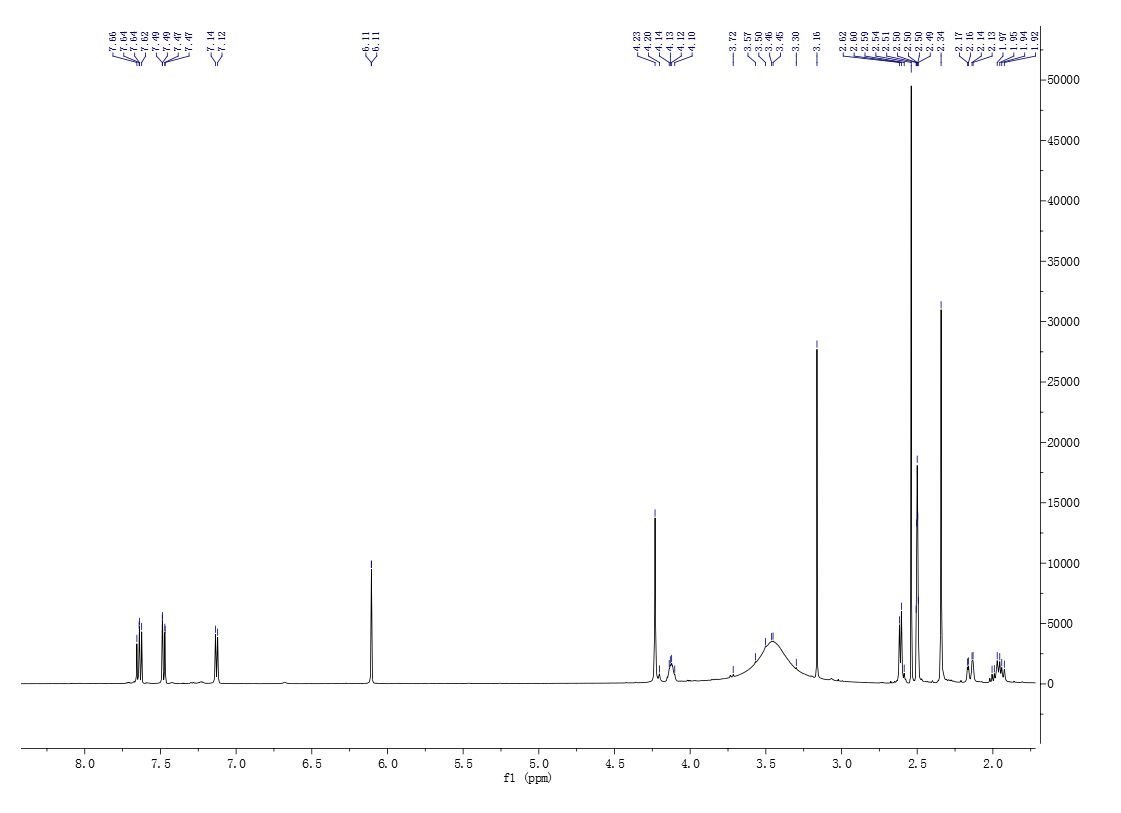
**

Figure S_14_. ^1^H-NMR spectrum of compound **2**

**
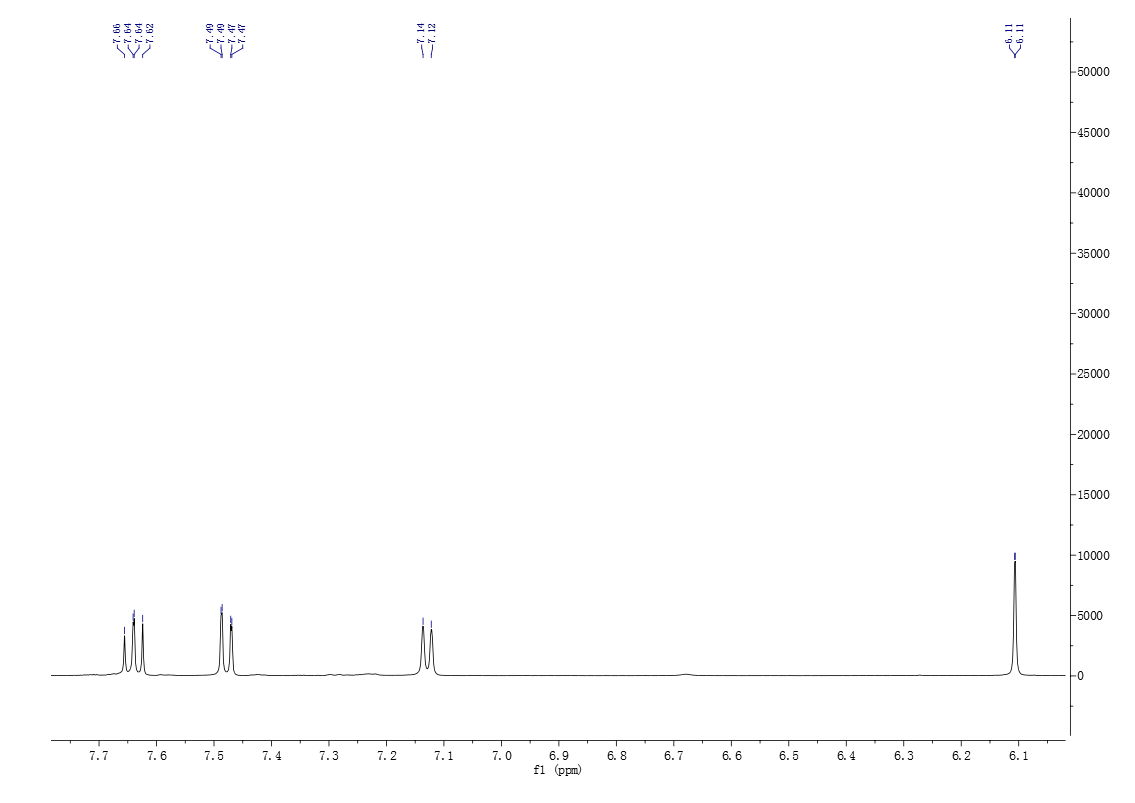
**

Figure S_15_. ^1^H-NMR spectrum of compound **2**

**
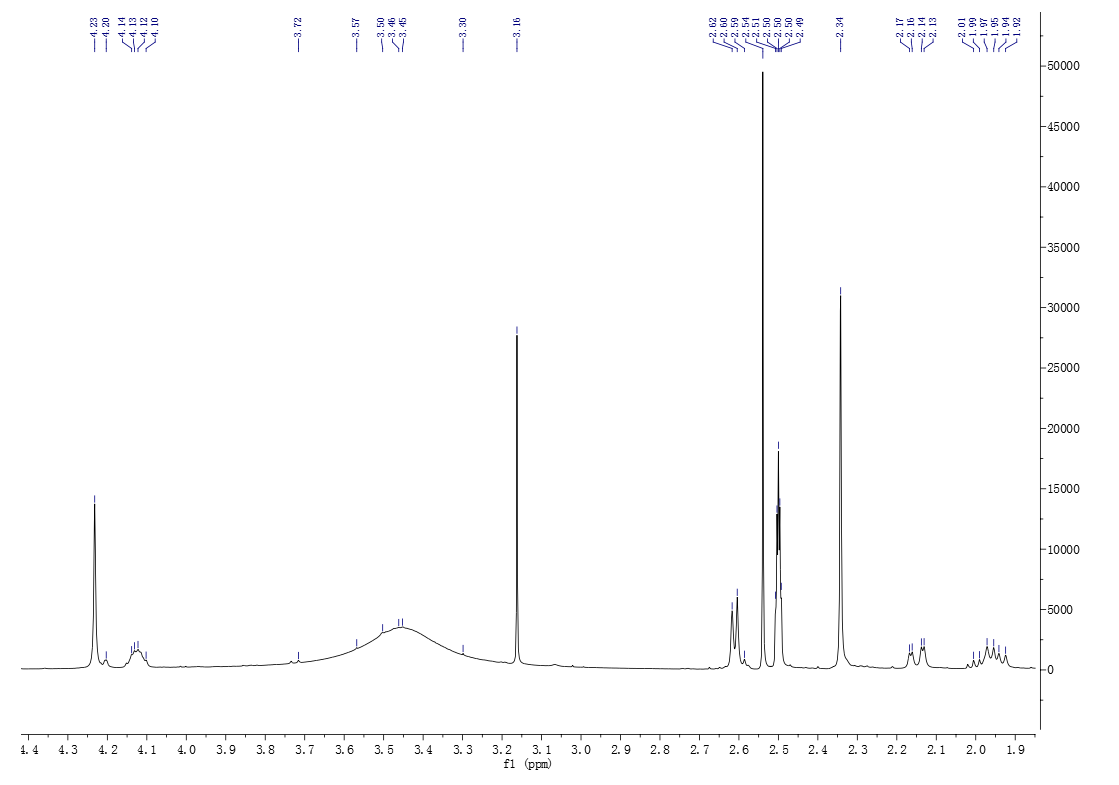
**

Figure S_16_. ^13^C-NMR spectrum of compound **2**

**
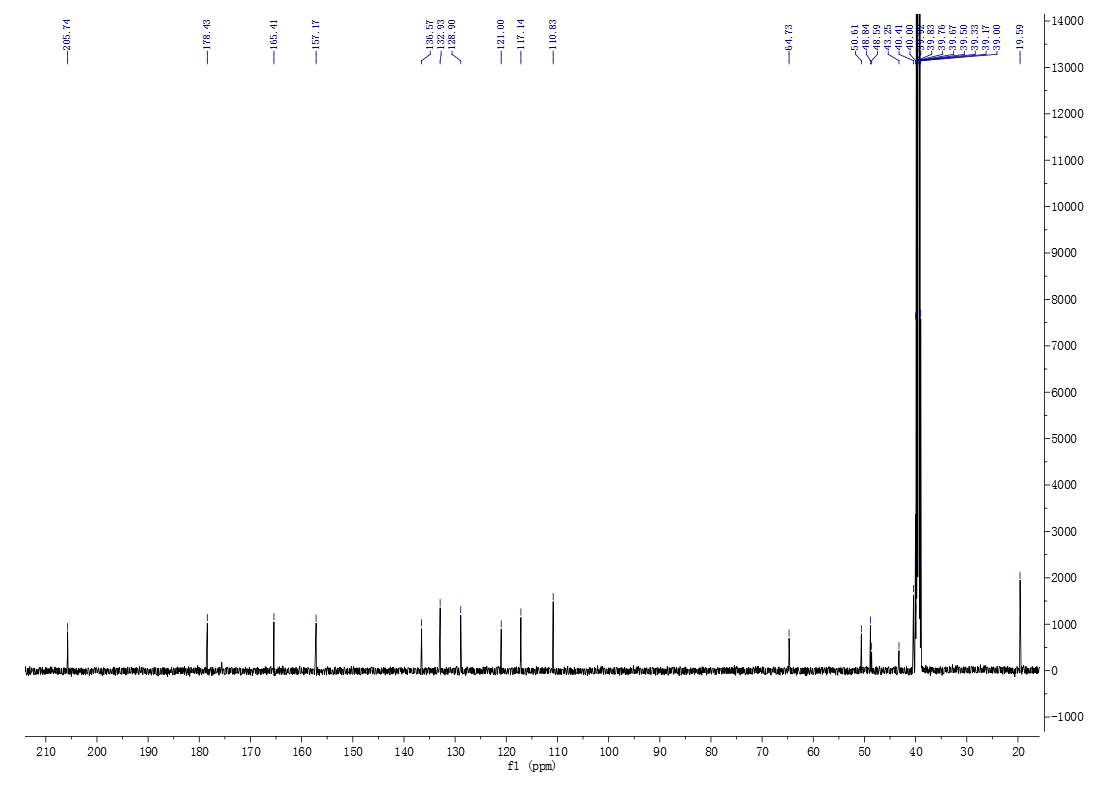
**

Figure S_17_. ^13^C-NMR spectrum of compound **2**

**
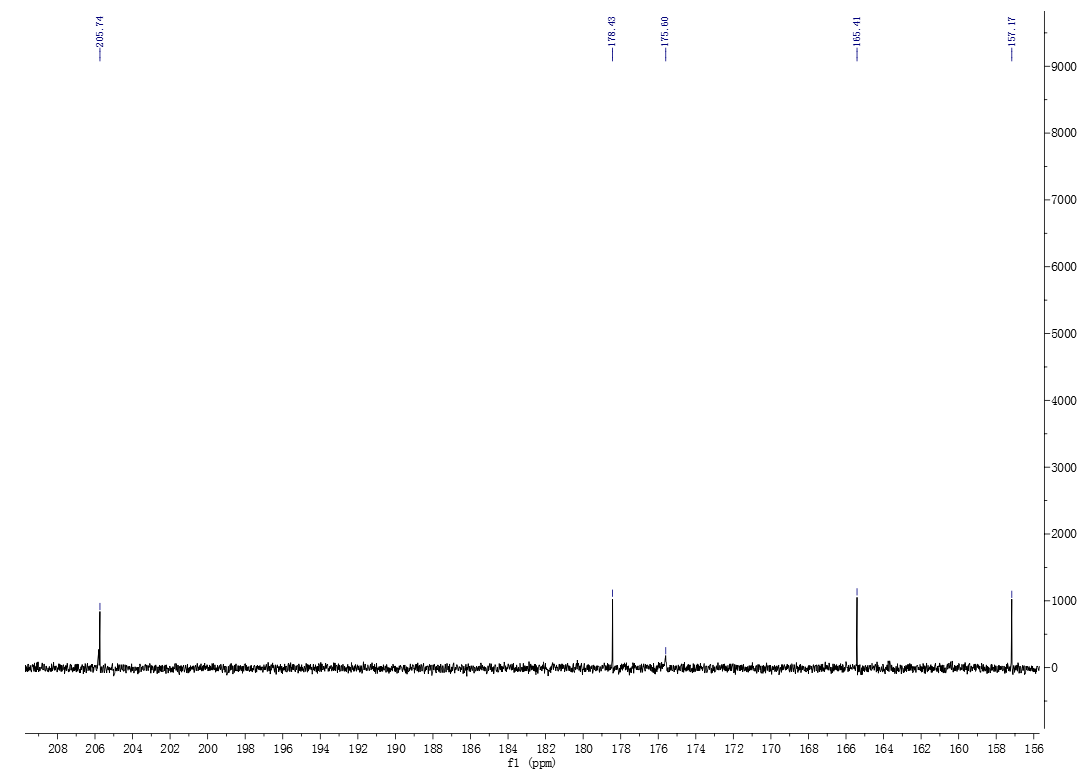
**

Figure S_18_. ^13^C-NMR spectrum of compound **2**

**
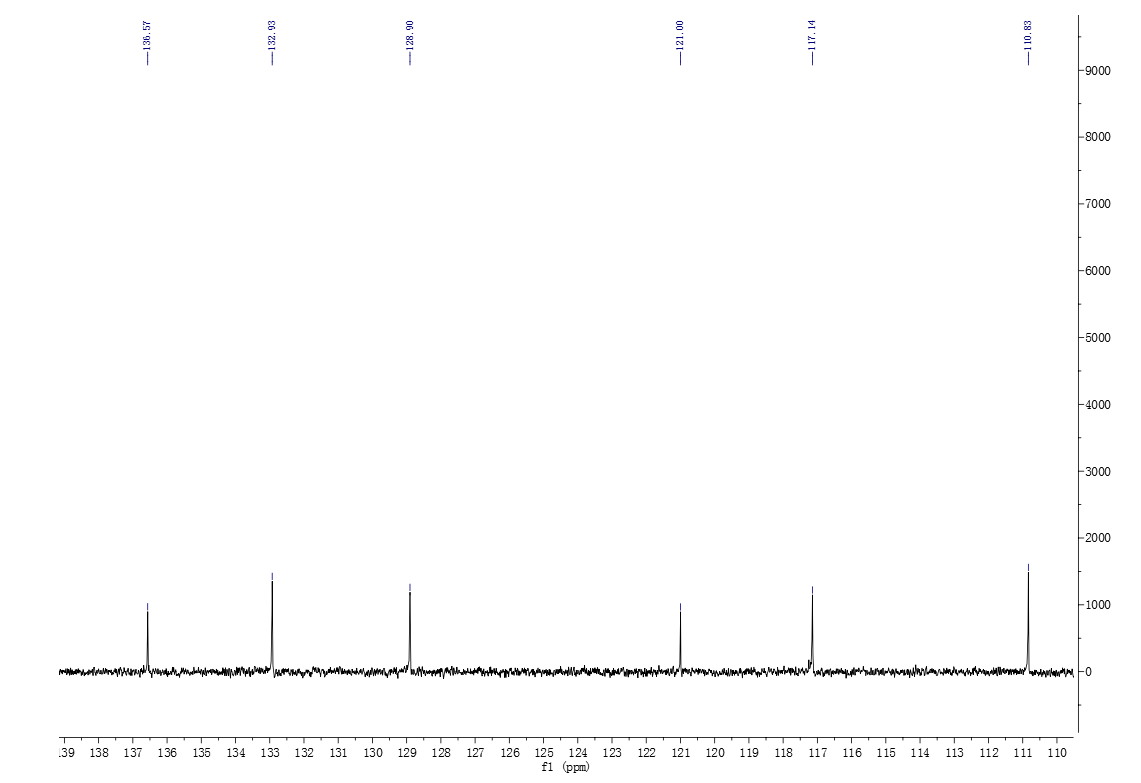
**

Figure S_19_. ^13^C-NMR spectrum of compound **2**

**
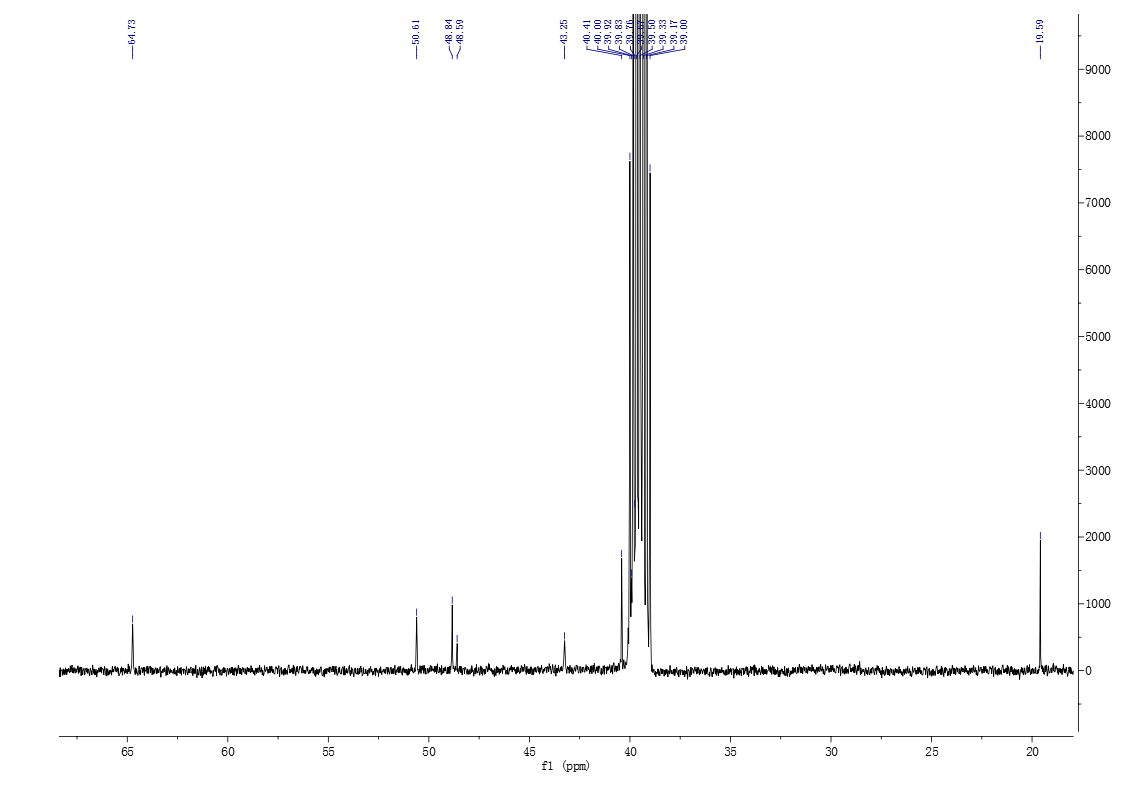
**

Figure S_20_. ^1^H-^1^H COSY spectrum of compound **2**

**
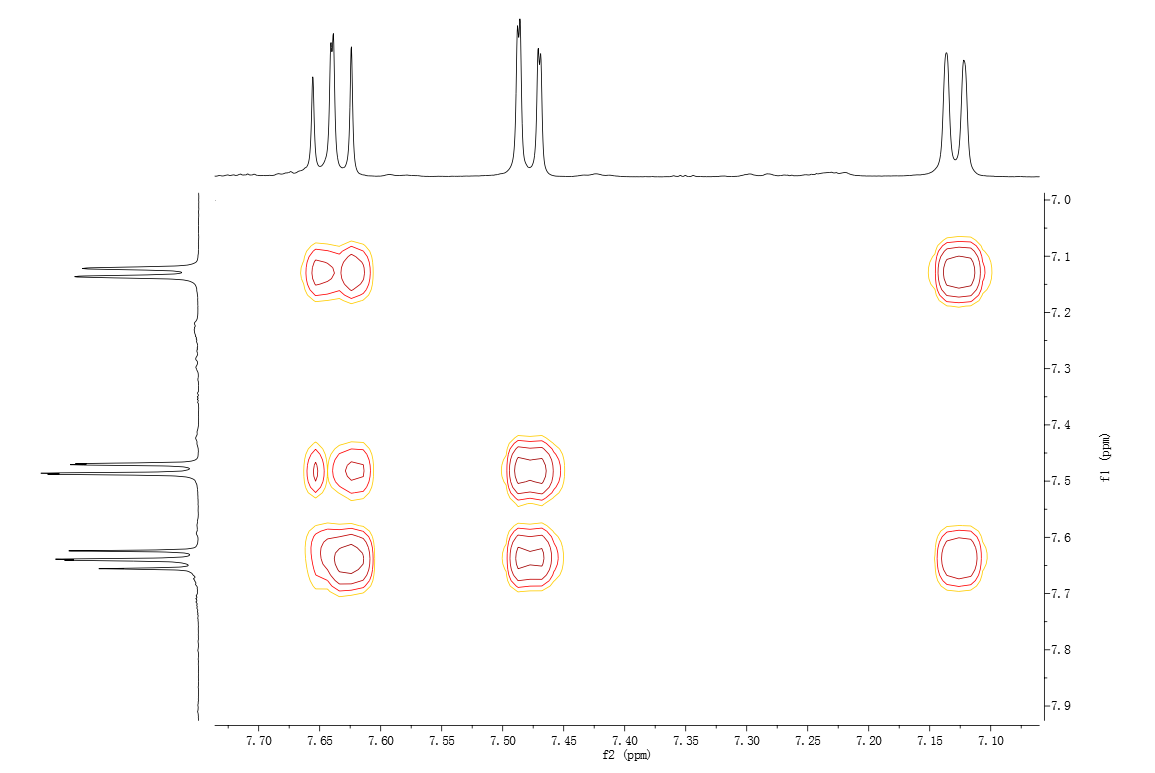
**

Figure S_21_. ^1^H-^1^H COSY spectrum of compound **2**

**
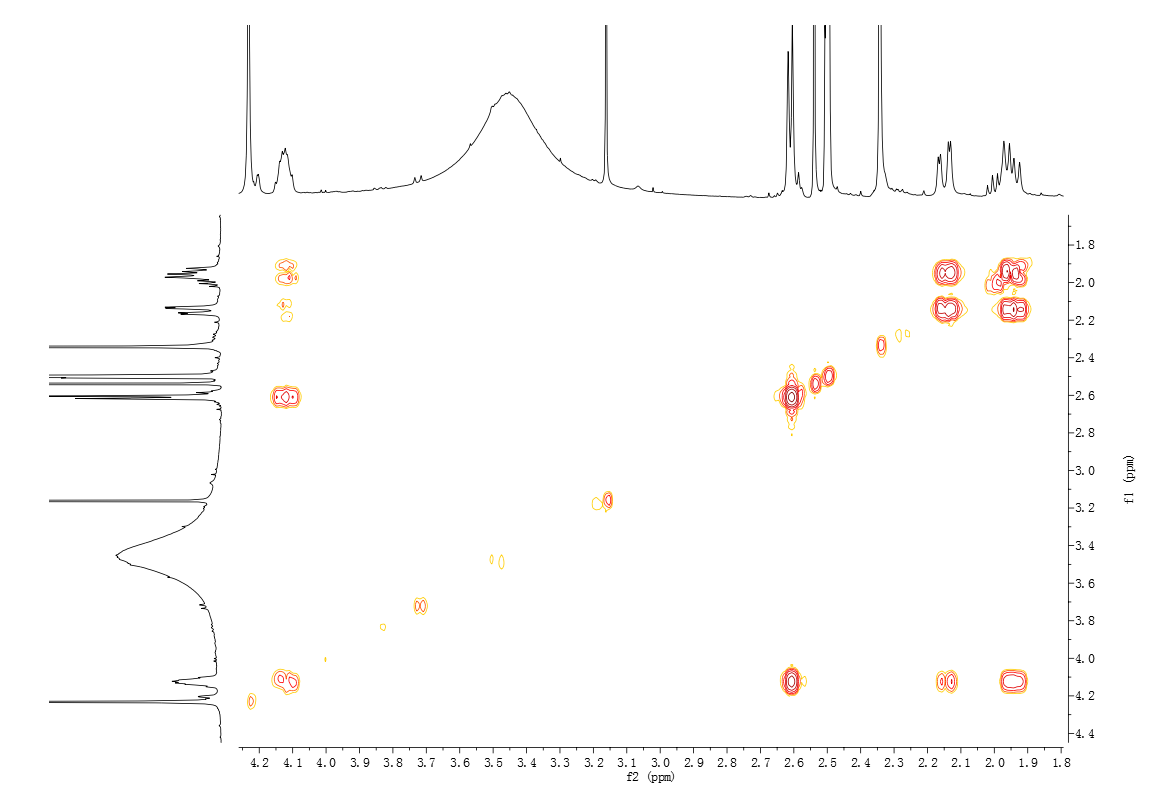
**

Figure S_22_. HSQC spectrum of compound **2**

**
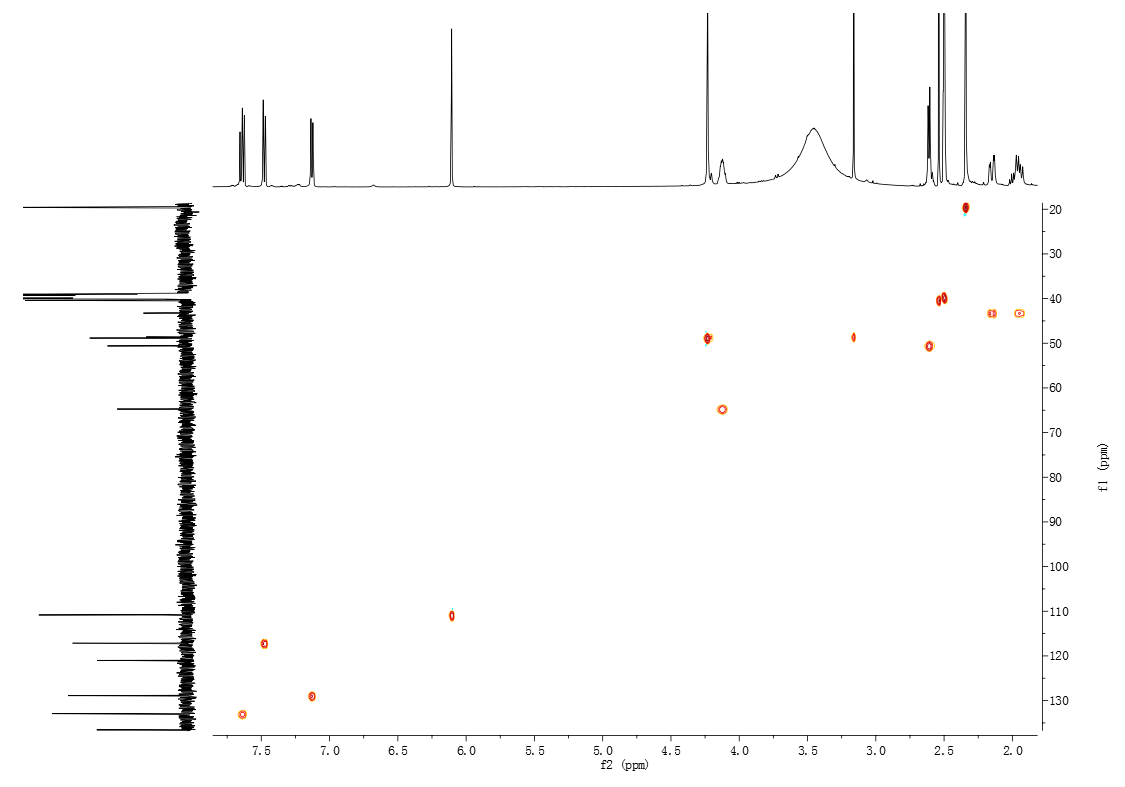
**

Figure S_23_. HSQC spectrum of compound **2**

**
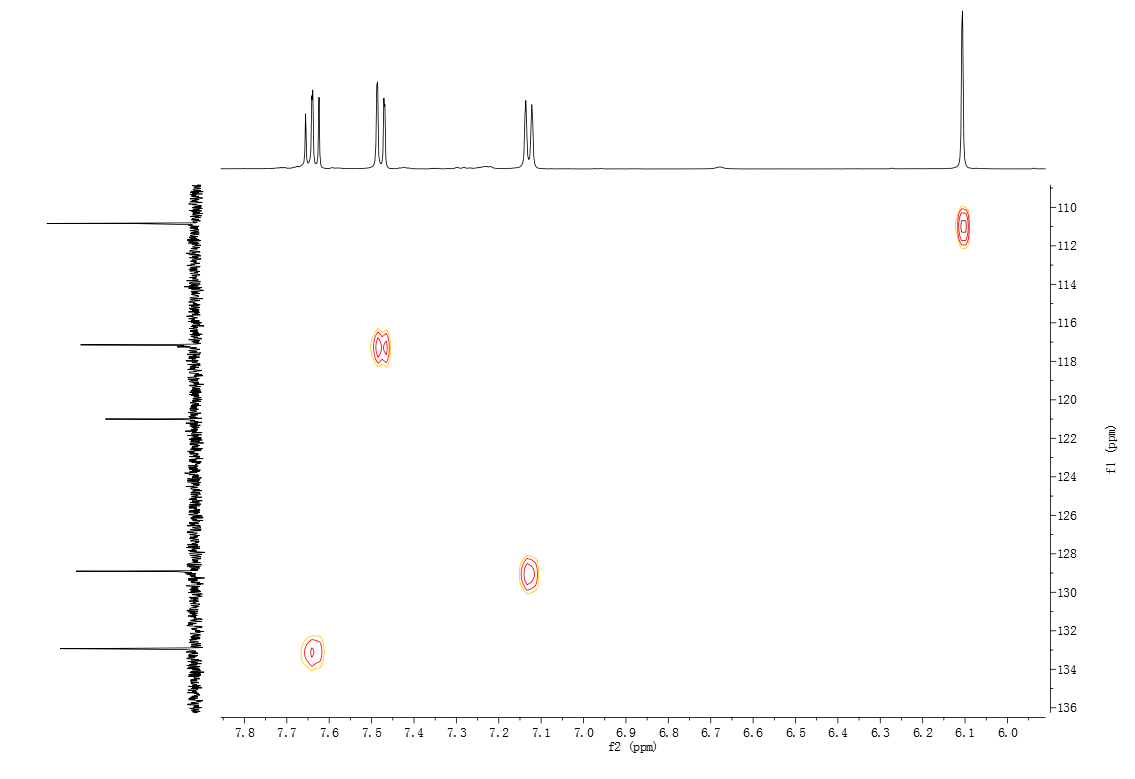
**

Figure S_24_. HSQC spectrum of compound **2**

**
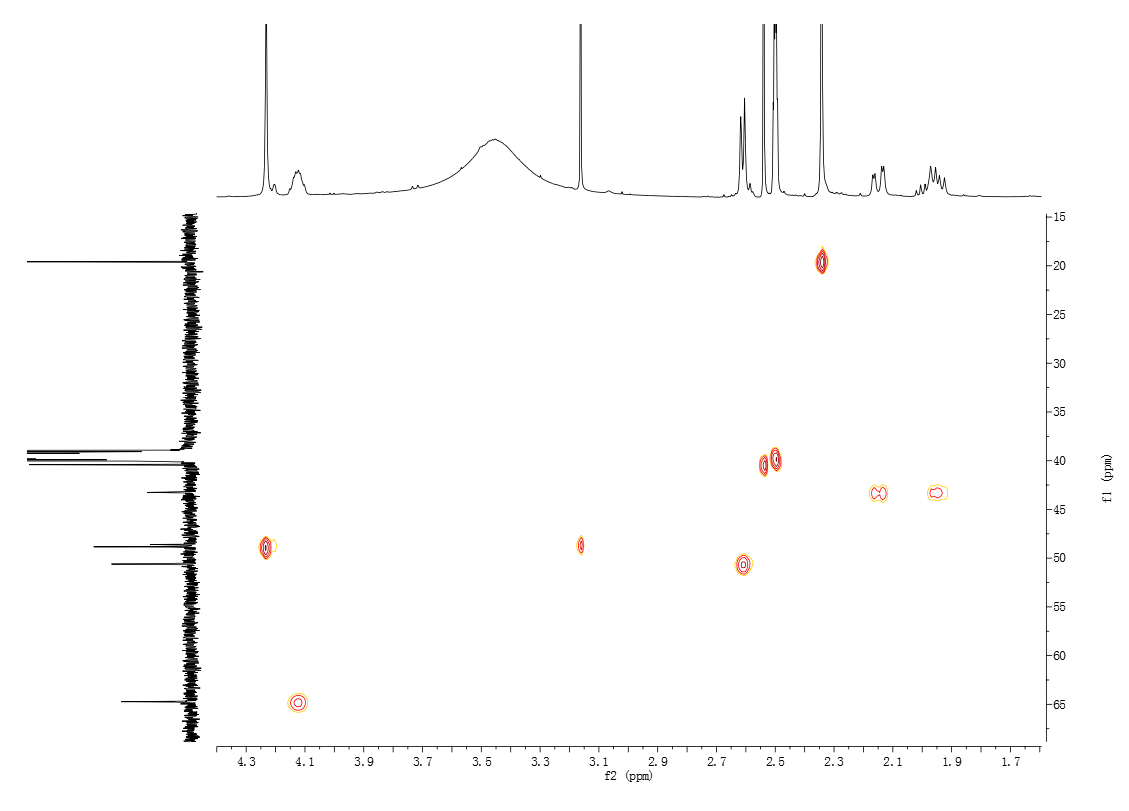
**

Figure S_25_. HMBC spectrum of compound **2**

**
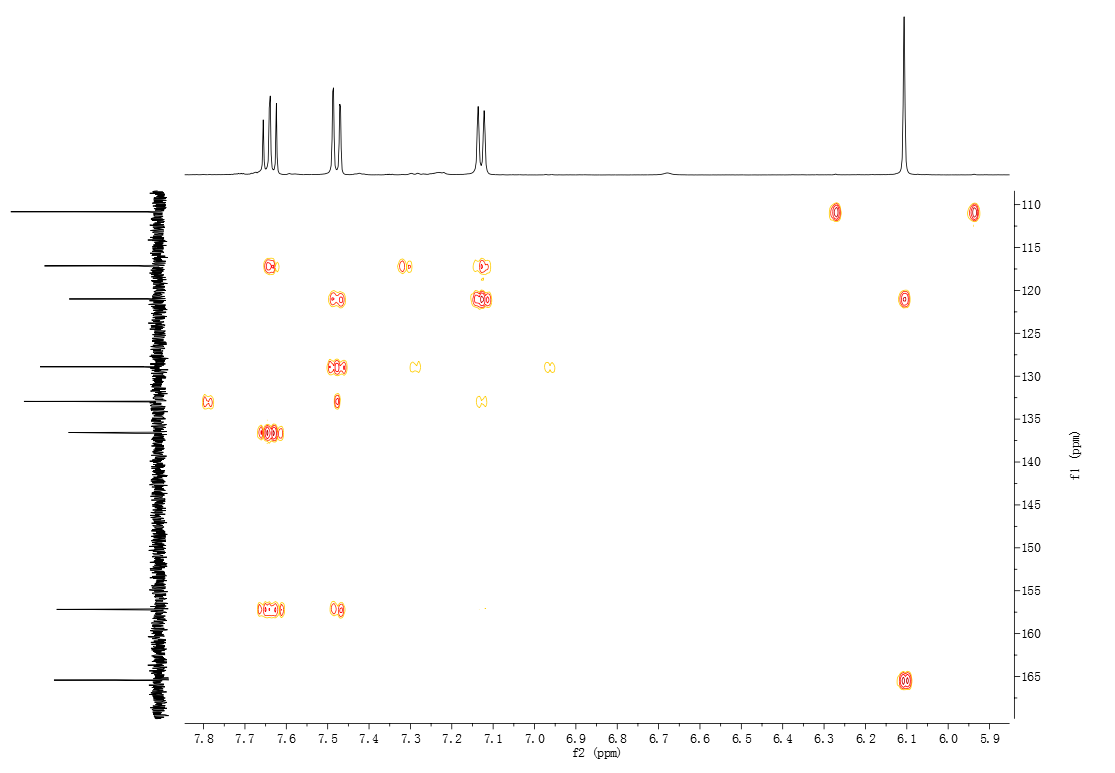
**

Figure S_26_. HMBC spectrum of compound **2**

**
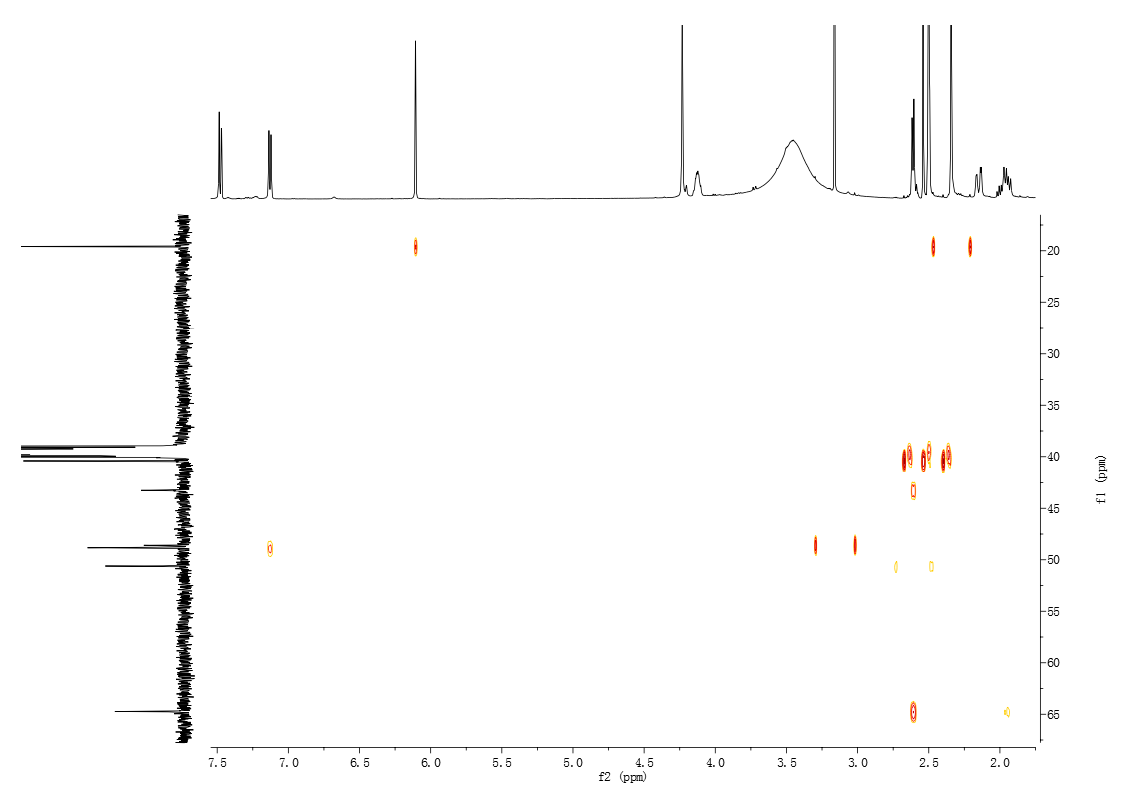
**

Figure S_27_. HMBC spectrum of compound **2**

**
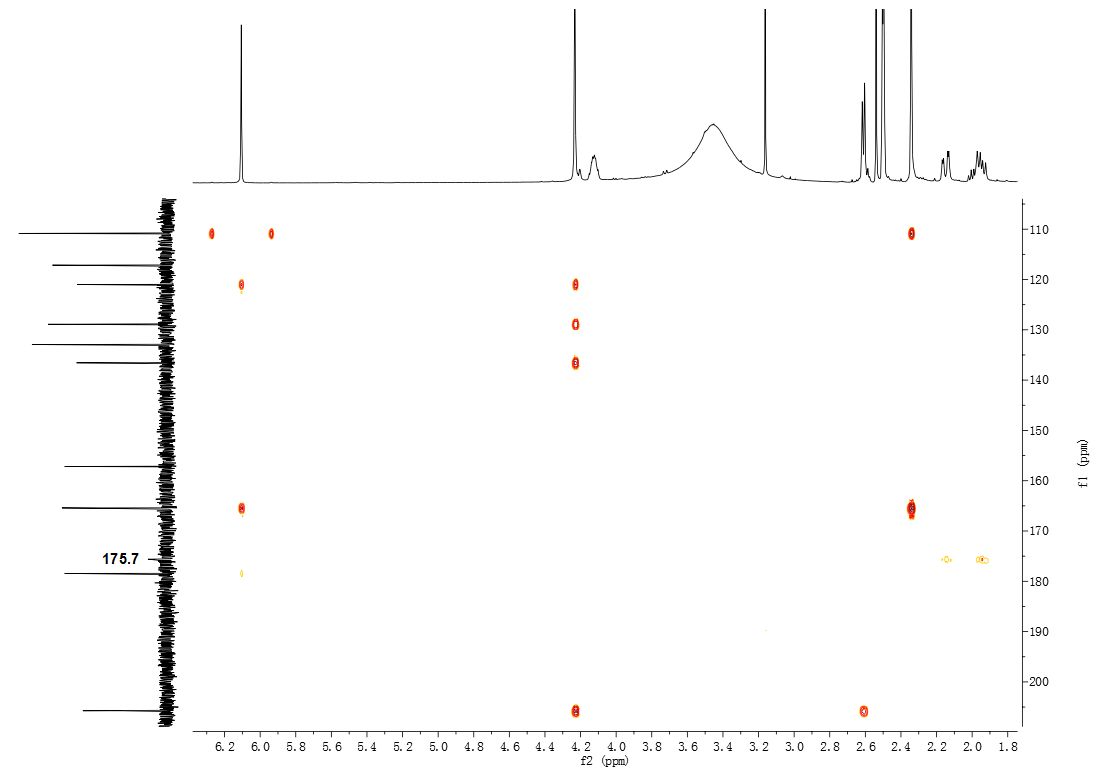
**

Figure S_28_. HRESIMS of compound **2**

**
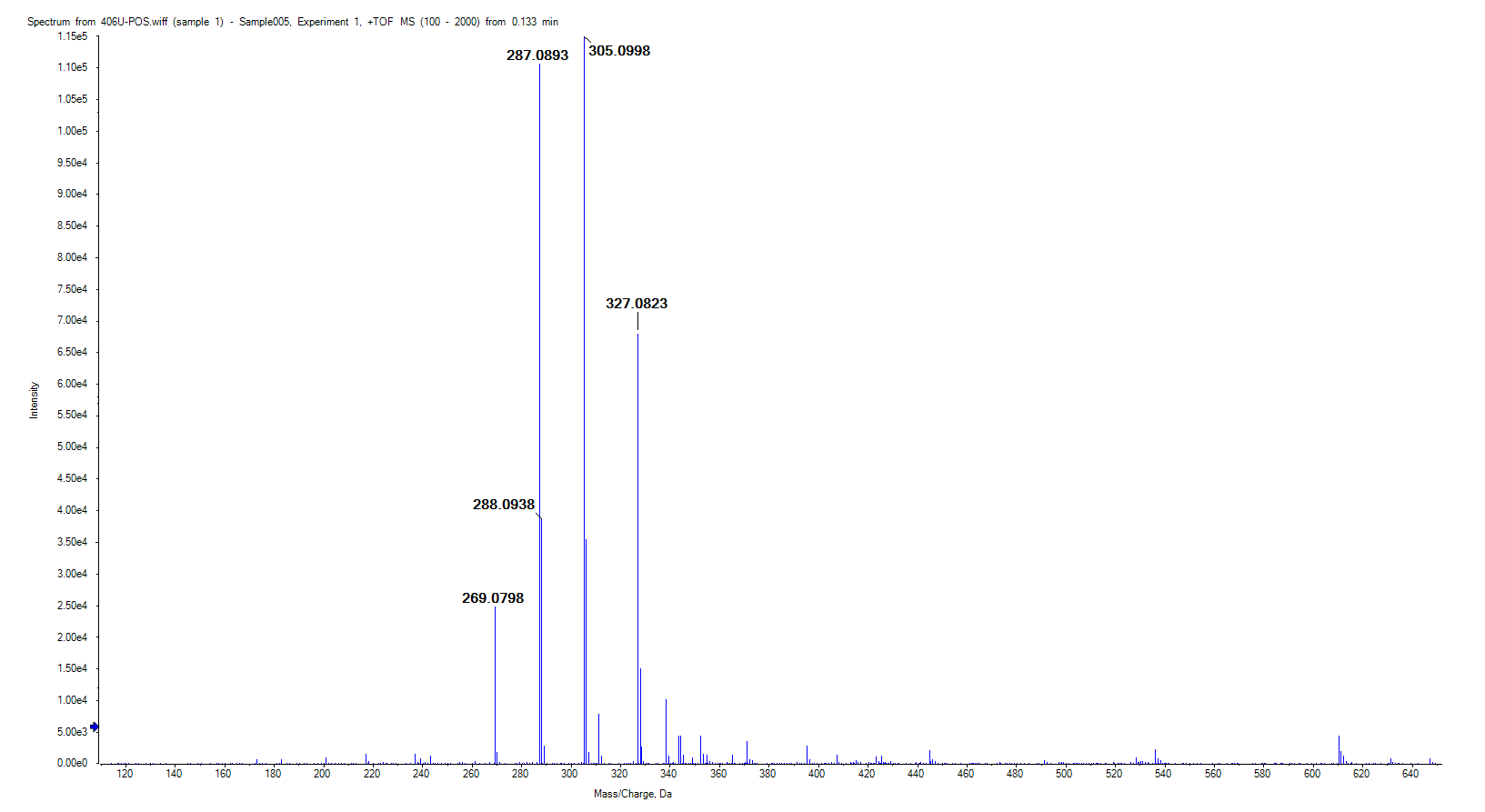
**

**Table S_4_. ^13^C and ^1^H NMR data for compound** 3

| No. | C | H (*J* = Hz) | No. | C | H (*J* = Hz) |
| --- | --- | --- | --- | --- | --- |
| 1 | 175.1, C | − | 8 | 51.2, CH | 4.29, 1H, m |
| 2 | 50.2, CH | 3.73, 1H, m | 8-NH | − | 8.03, 1H, d (8.3) |
| 2-NH | − | 7.60, 1H, d (7.4) | 9 | 40.8, CH_2_ | 1.45, 2H, m |
| 3 | 18.5, CH_3_ | 1.14, 3H, d (7.1) | 10 | 24.3, CH | 1.58, 1H, m |
| 4 | 169.1, C | − | 11 | 21.4*^a^*, CH_3_ | 0.83*^a^*, 3H, d (6.6) |
| 5 | 55.1, CH | 4.15, 1H, m | 12 | 23.1*^a^*, CH_3_ | 0.86*^a^*, 3H, d (6.6) |
| 5-NH | − | 7.77, 1H, d (7.7) | 13 | 169.4, C | − |
| 6 | 62.1, CH_2_ | 3.38, 1H, dd (10.9, 6.8);  3.54, 1H, dd (10.9, 5.3) | 14 | 22.5, CH_3_ | 1.83, 3H, s |
| 7 | 172.4, C | − |  |  |  |

*^a^* The data with the same label in each column may be interchanged.

Figure S_29_. **^1^**H-NMR spectrum of compound **3**


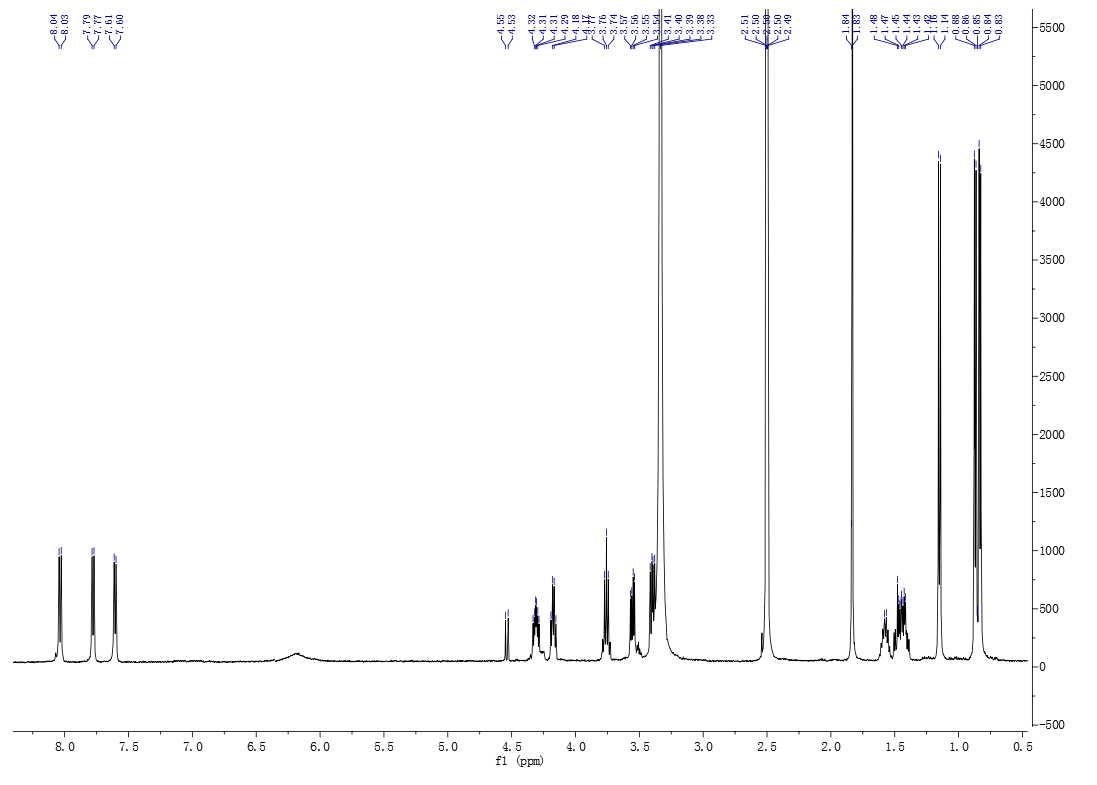


Figure S_30_. **^1^**H-NMR spectrum of compound **3**


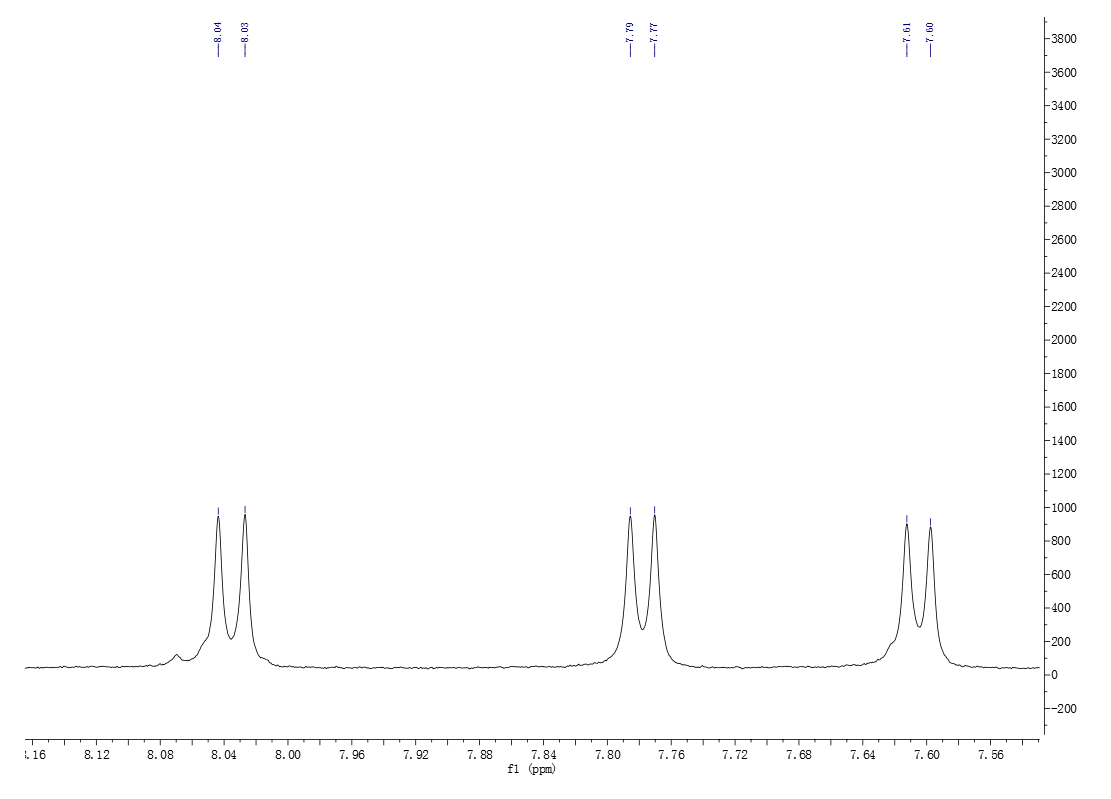


Figure S_31_. **^1^**H-NMR spectrum of compound **3**


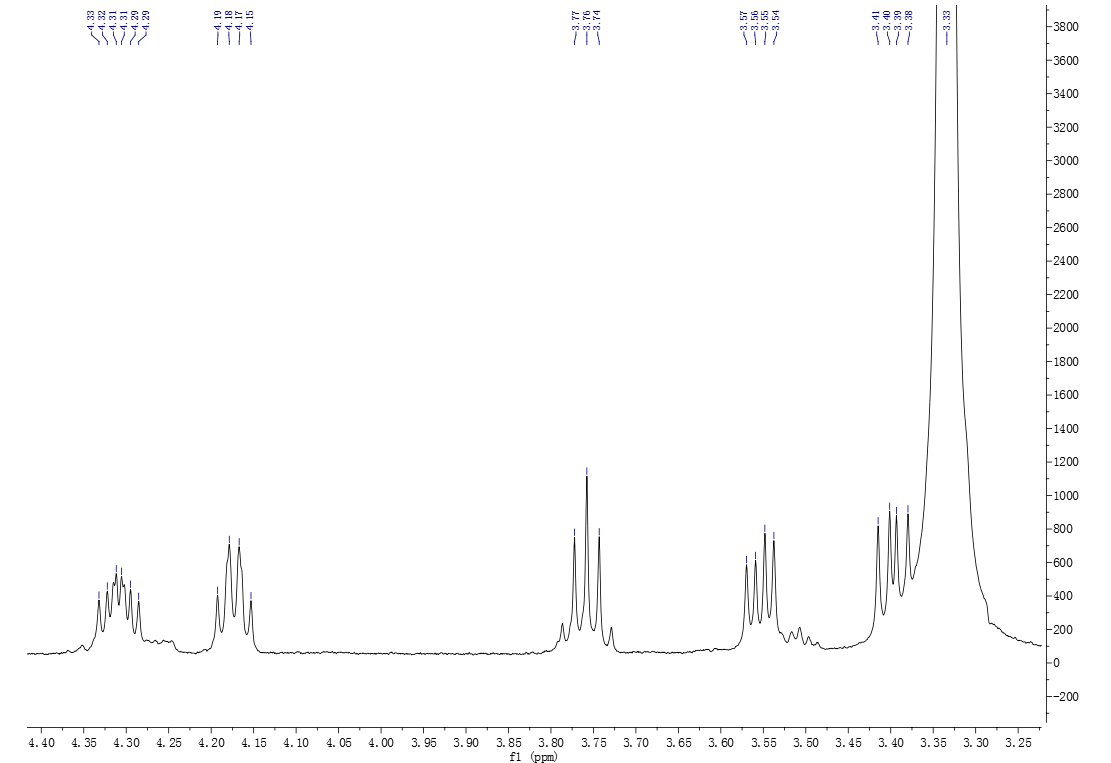


Figure S_32_. ^1^H-NMR spectrum of compound **3**

**
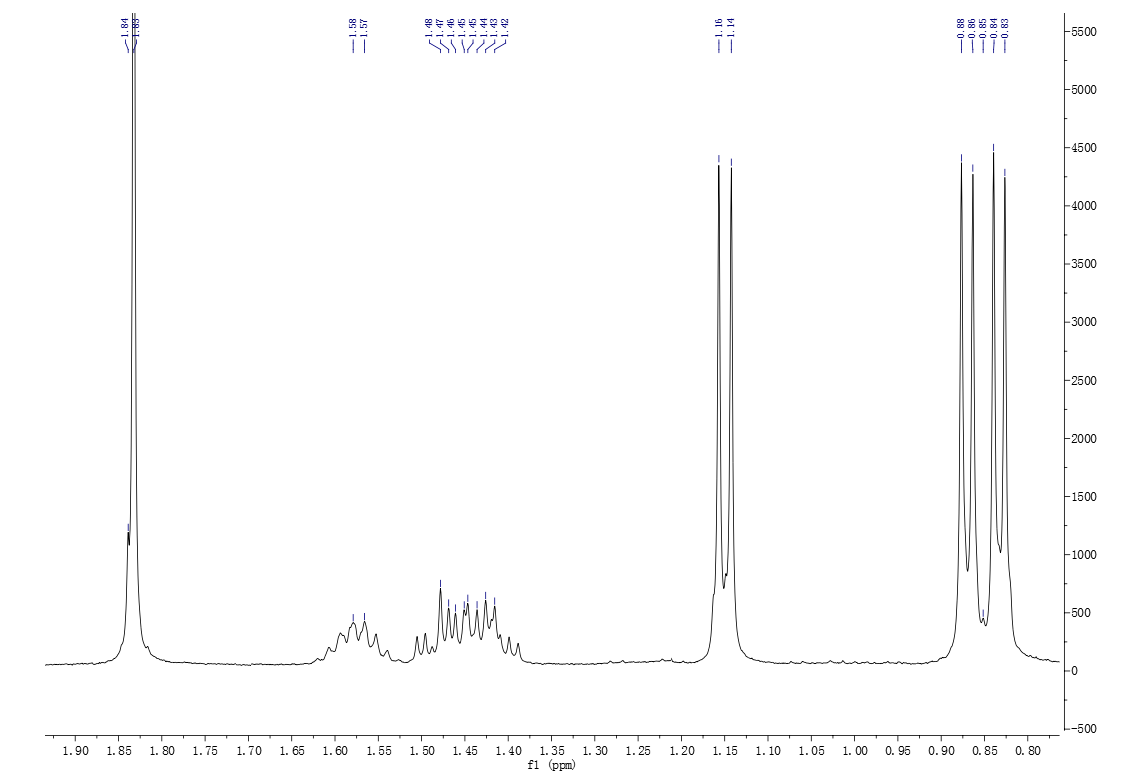
**

Figure S_33_. ^13^C-NMR spectrum of compound **3**


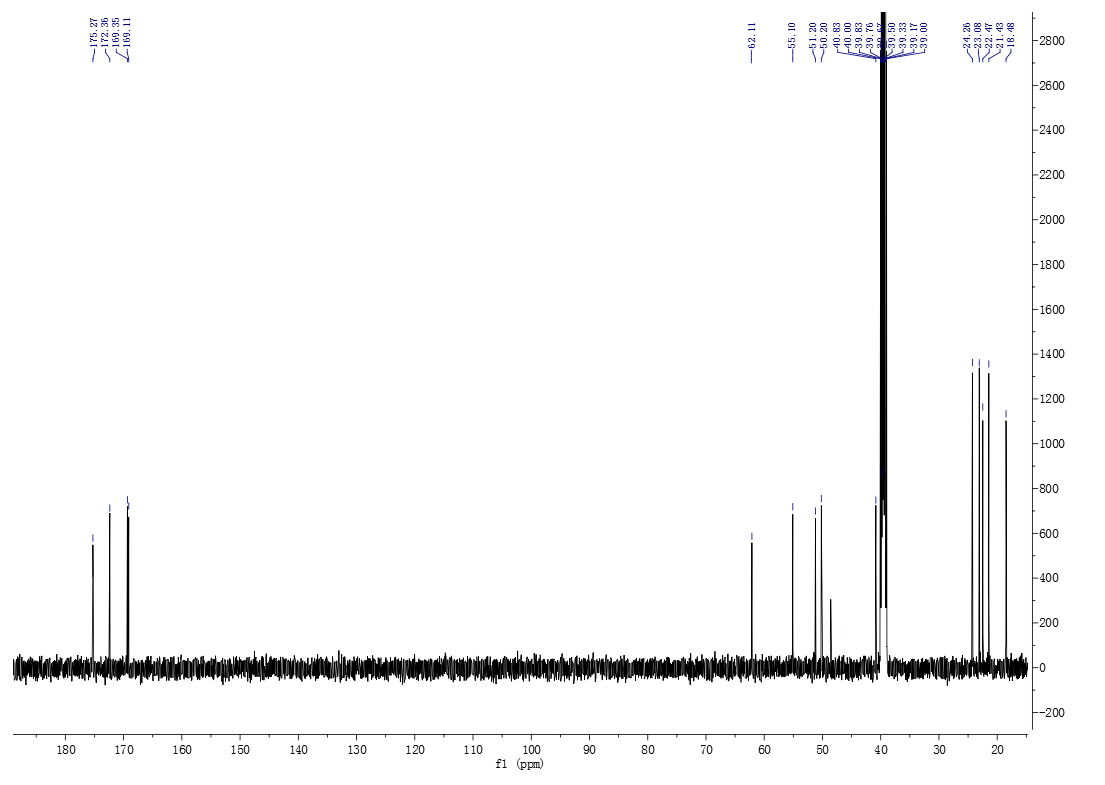


Figure S_34_. ^13^C-NMR spectrum of compound **3**


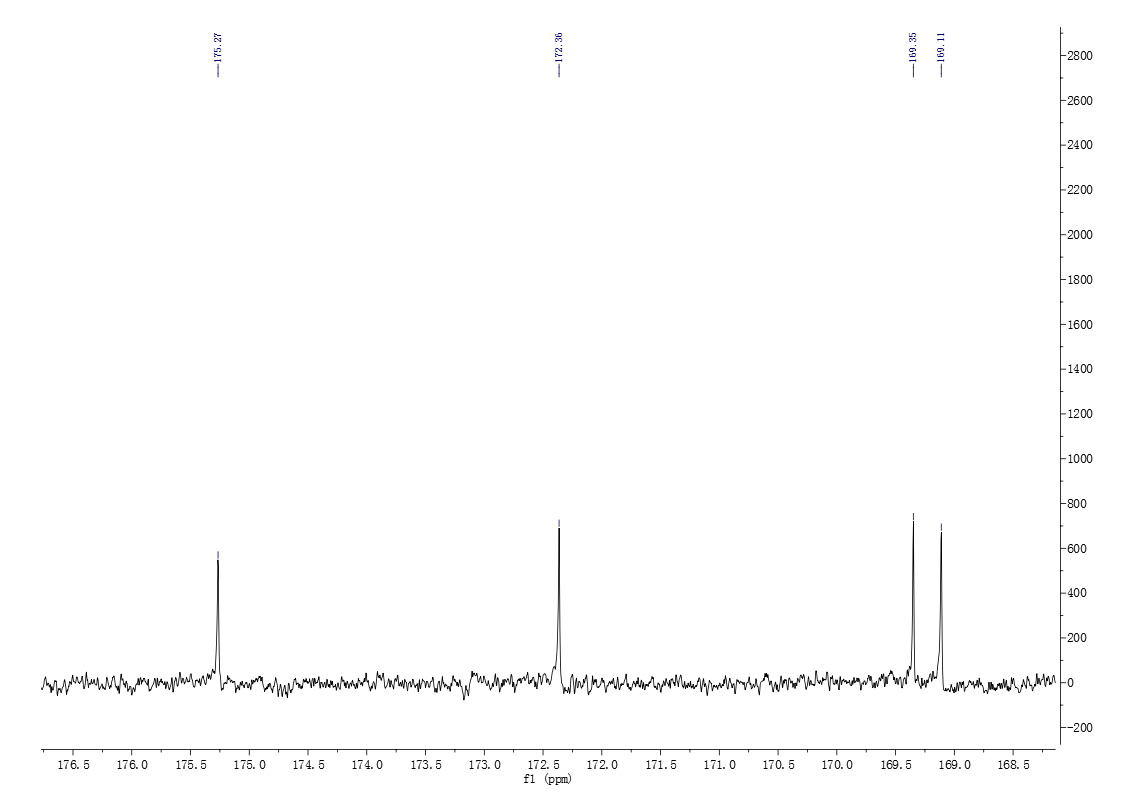


Figure S_35_. ^13^C-NMR spectrum of compound **3**


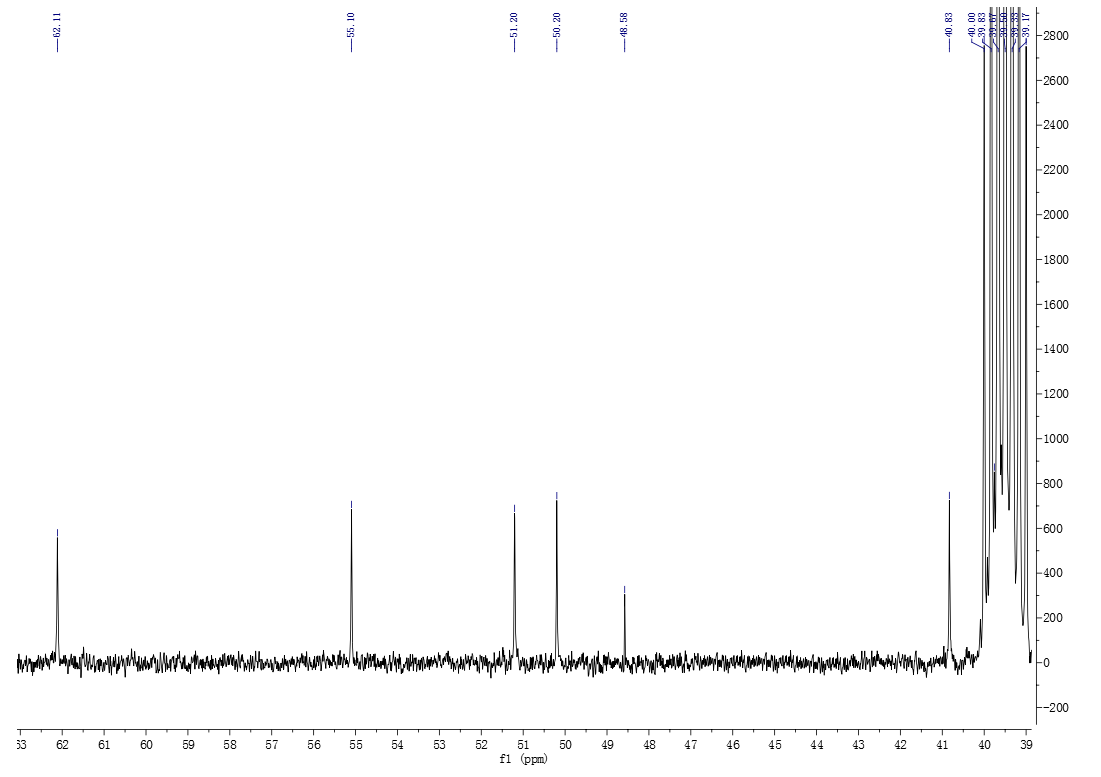


Figure S_36_. ^13^C-NMR spectrum of compound **3**


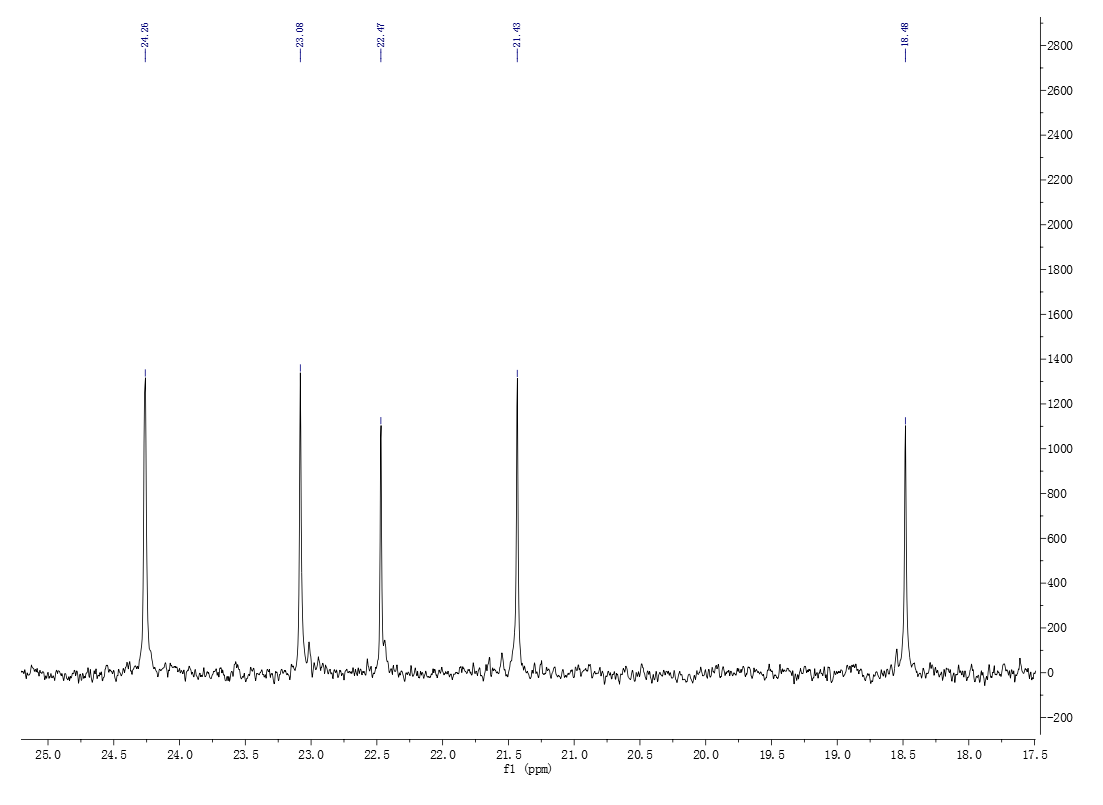


Figure S_37_. HSQC spectrum of compound **3**

**
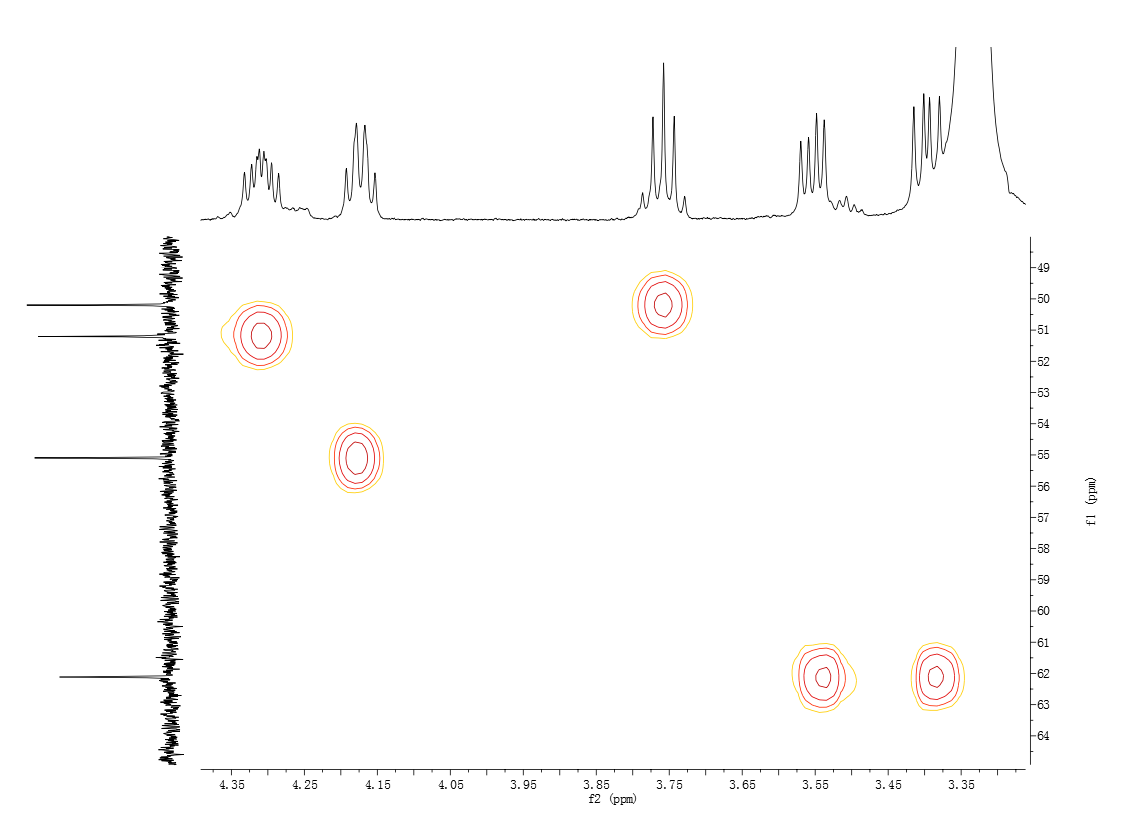
**

Figure S_38_. HSQC spectrum of compound **3**

**
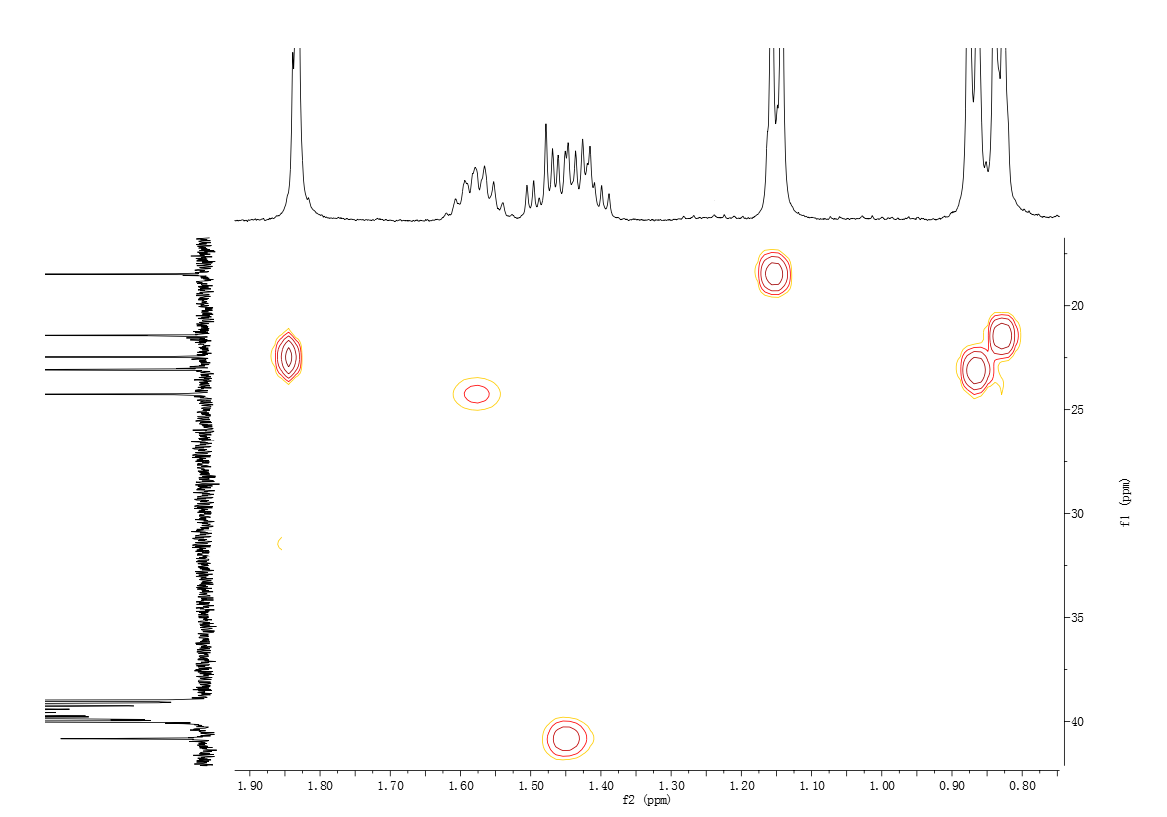
**

Figure S_39_. ^1^H-^1^H COSY spectrum of compound **3**


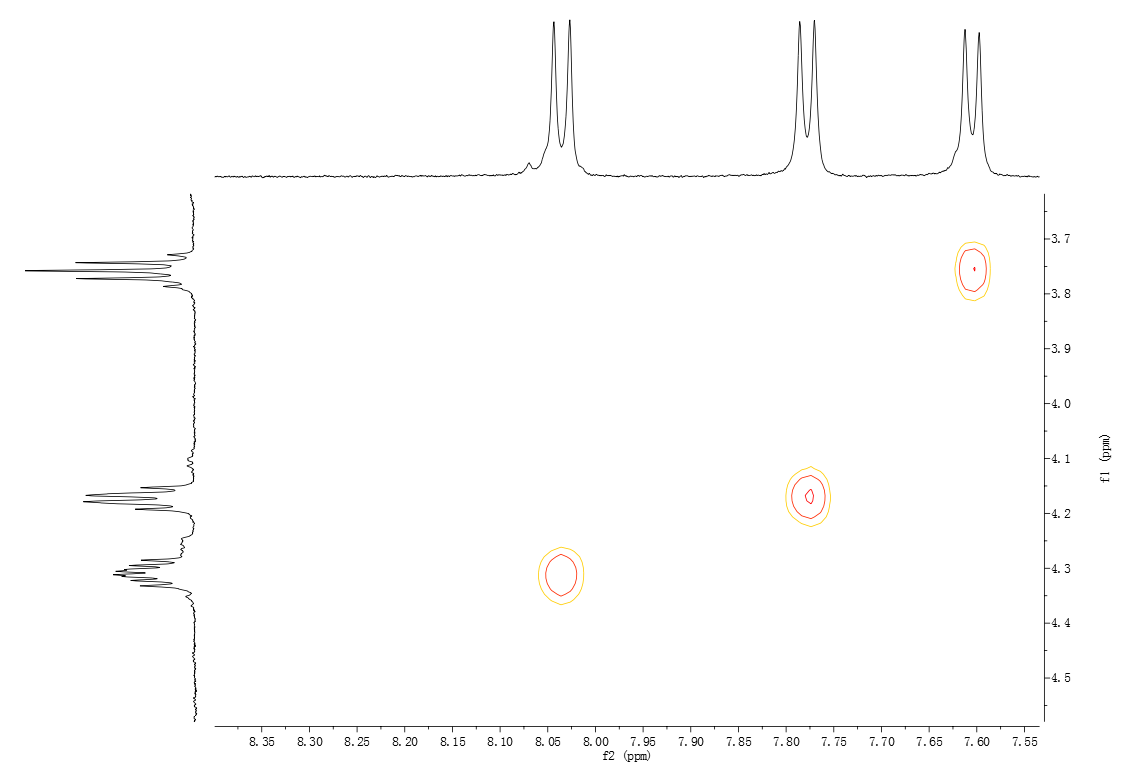


Figure S_40_. ^1^H-^1^H COSY spectrum of compound **3**


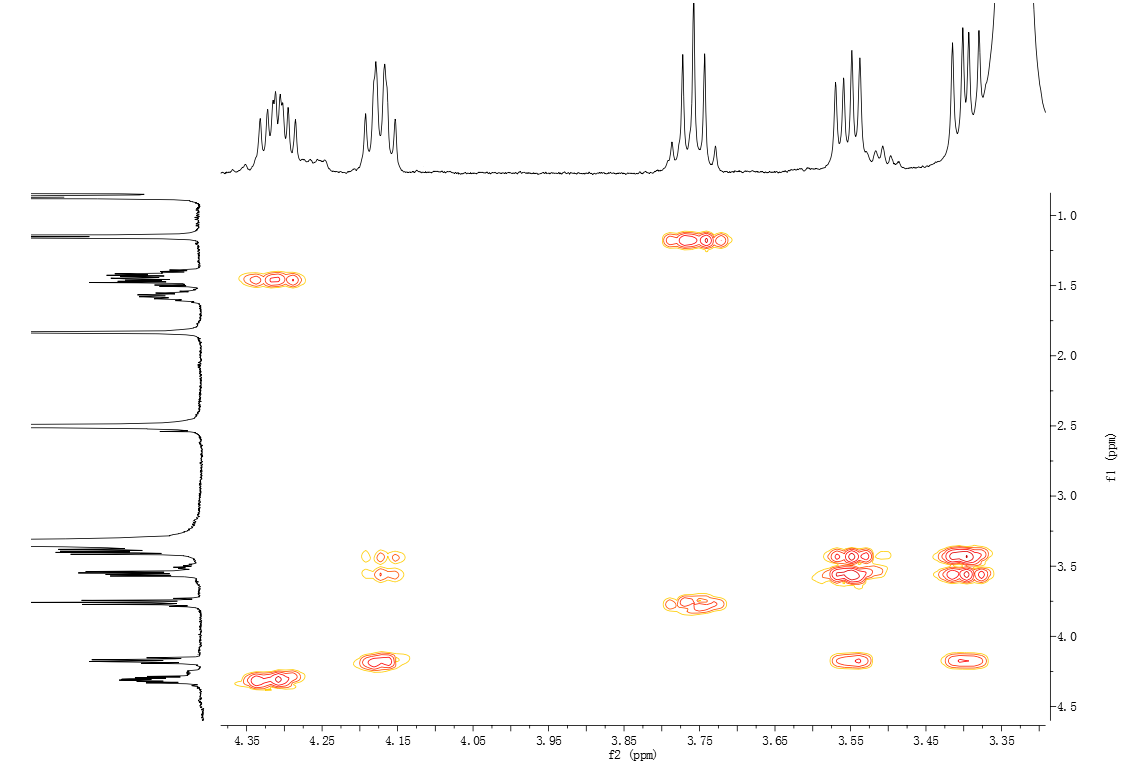


Figure S_41_. ^1^H-^1^H COSY spectrum of compound **3**


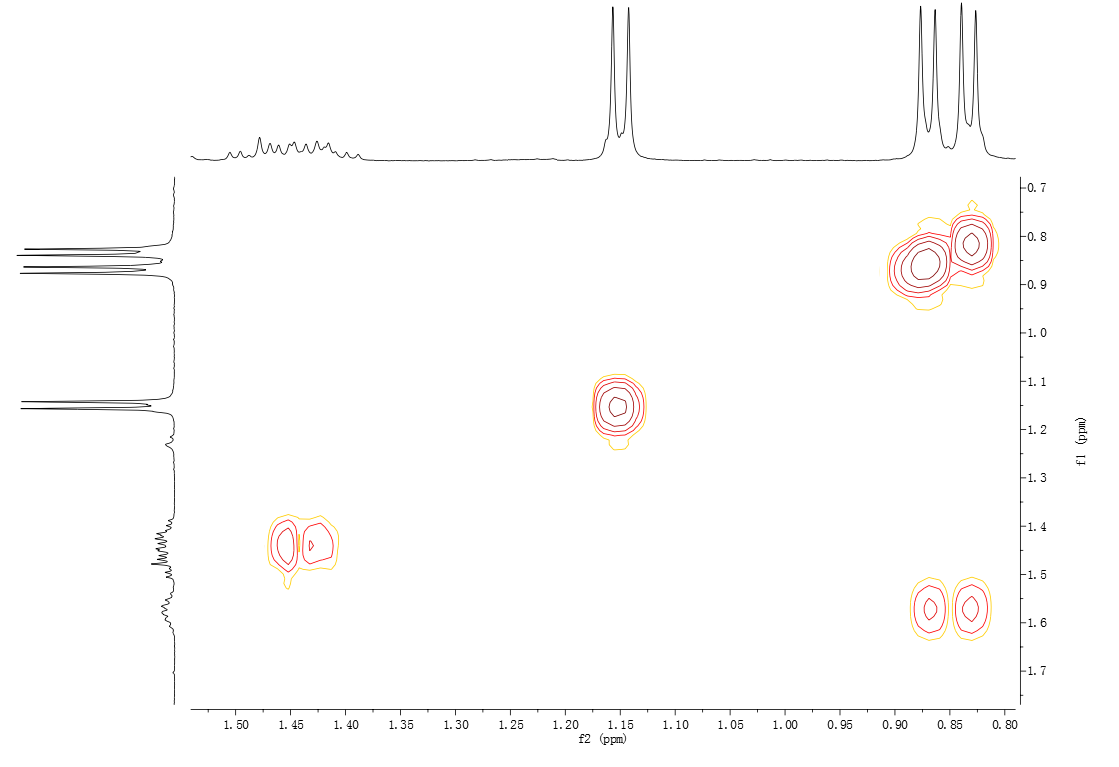


Figure S_42_. HMBC spectrum of compound **3**

**
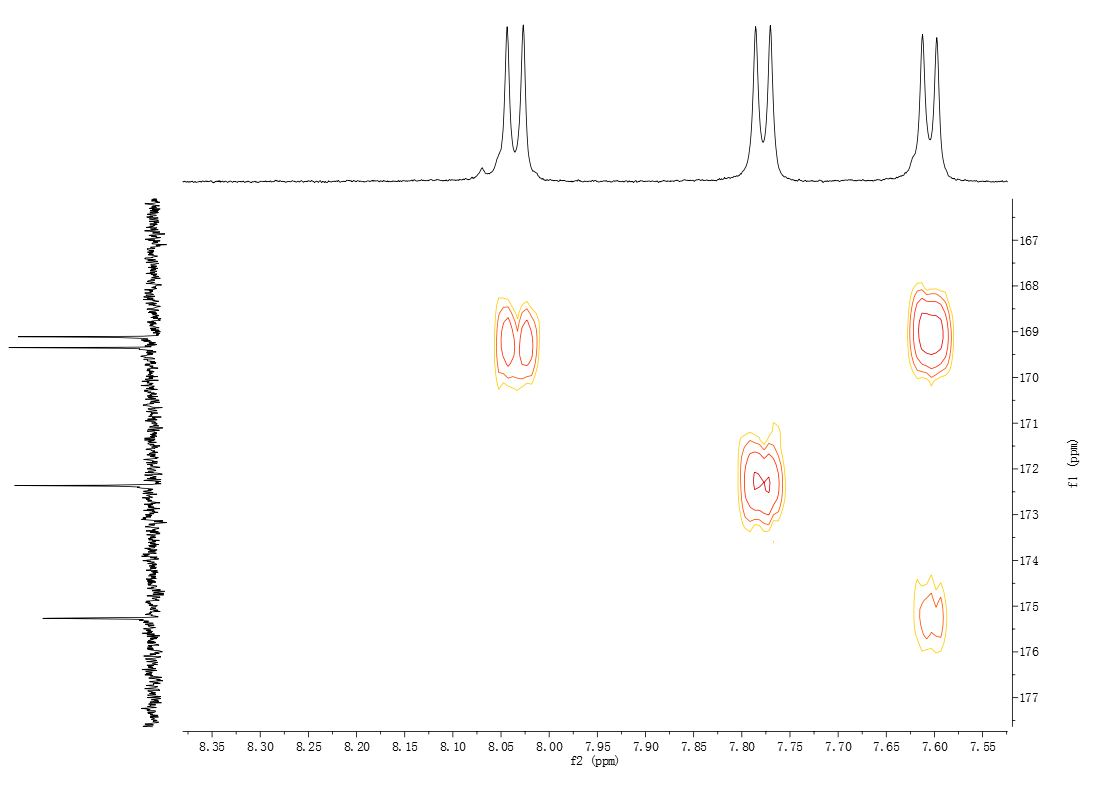
**

Figure S_43_. HMBC spectrum of compound **3**

**
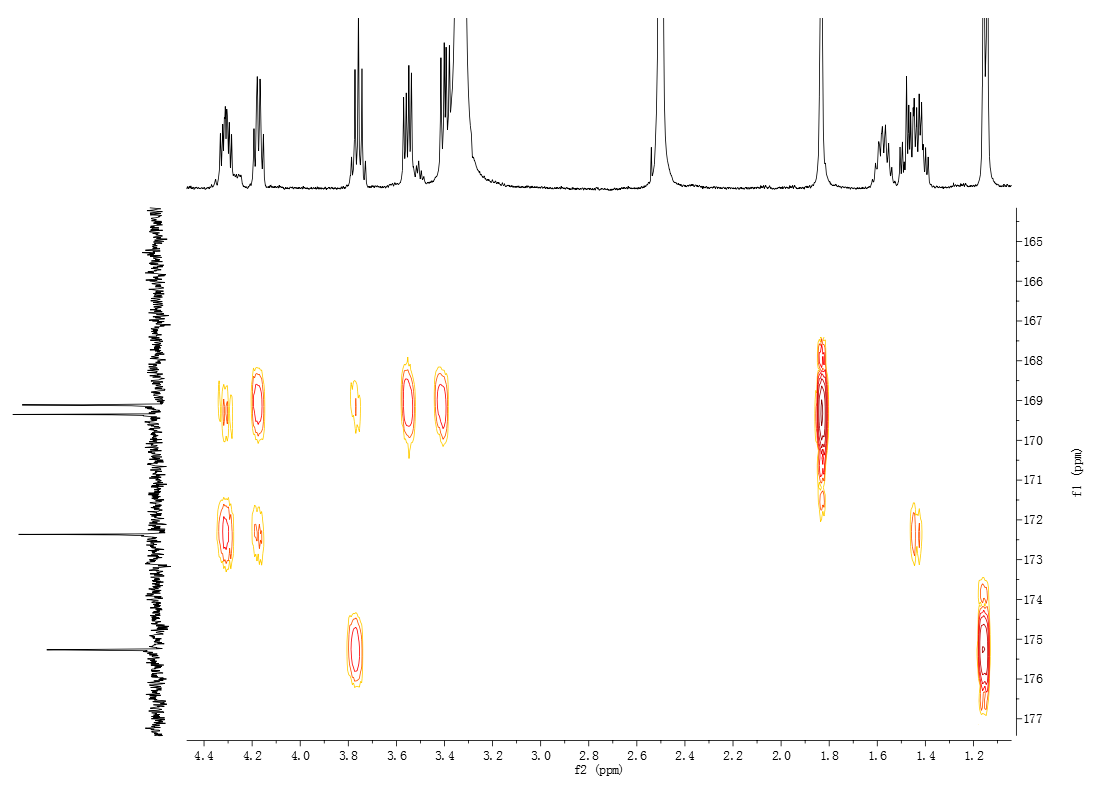
**

Figure S_44_. HMBC spectrum of compound **3**

**
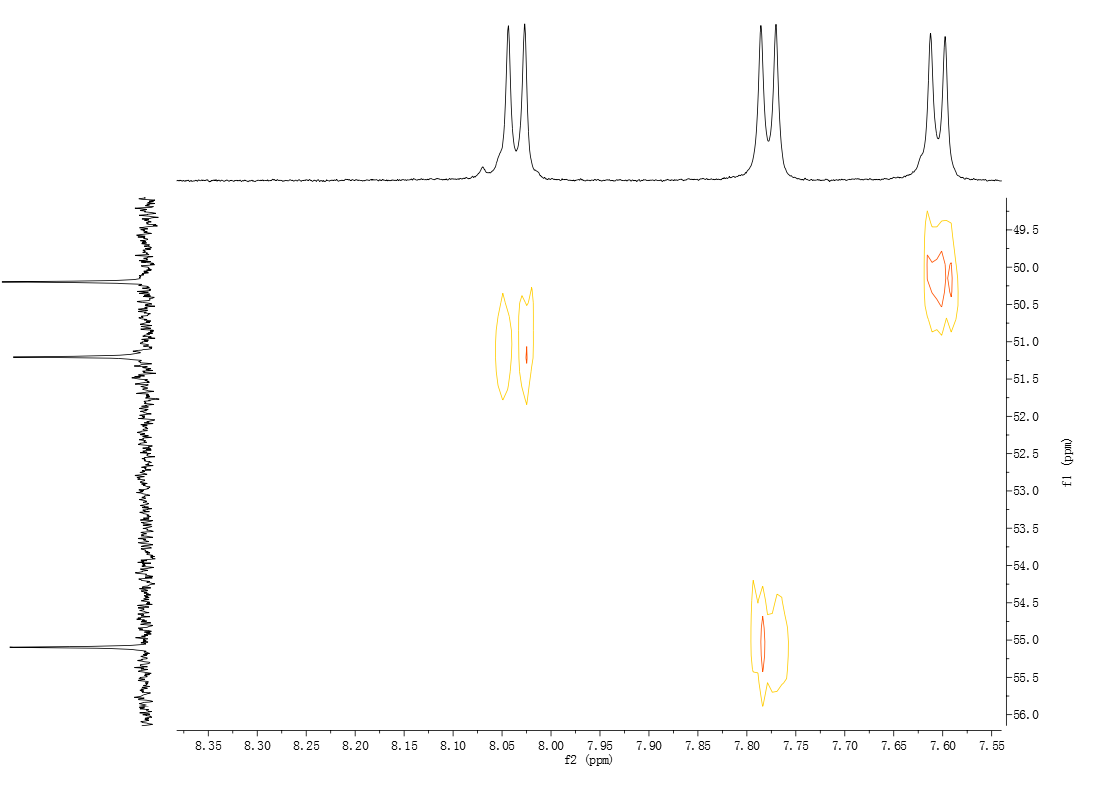
**

Figure S_45_. HMBC spectrum of compound **3**

**
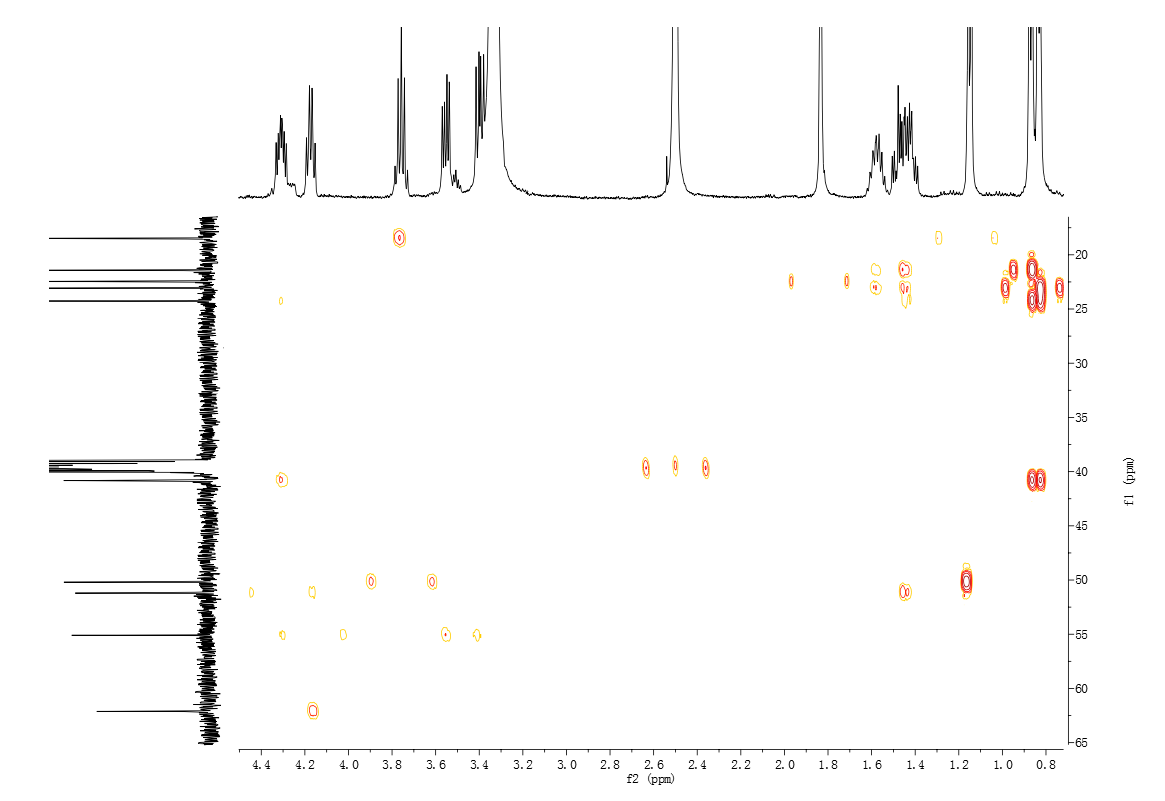
**

Figure S_46_. HRESIMS of compound **3**


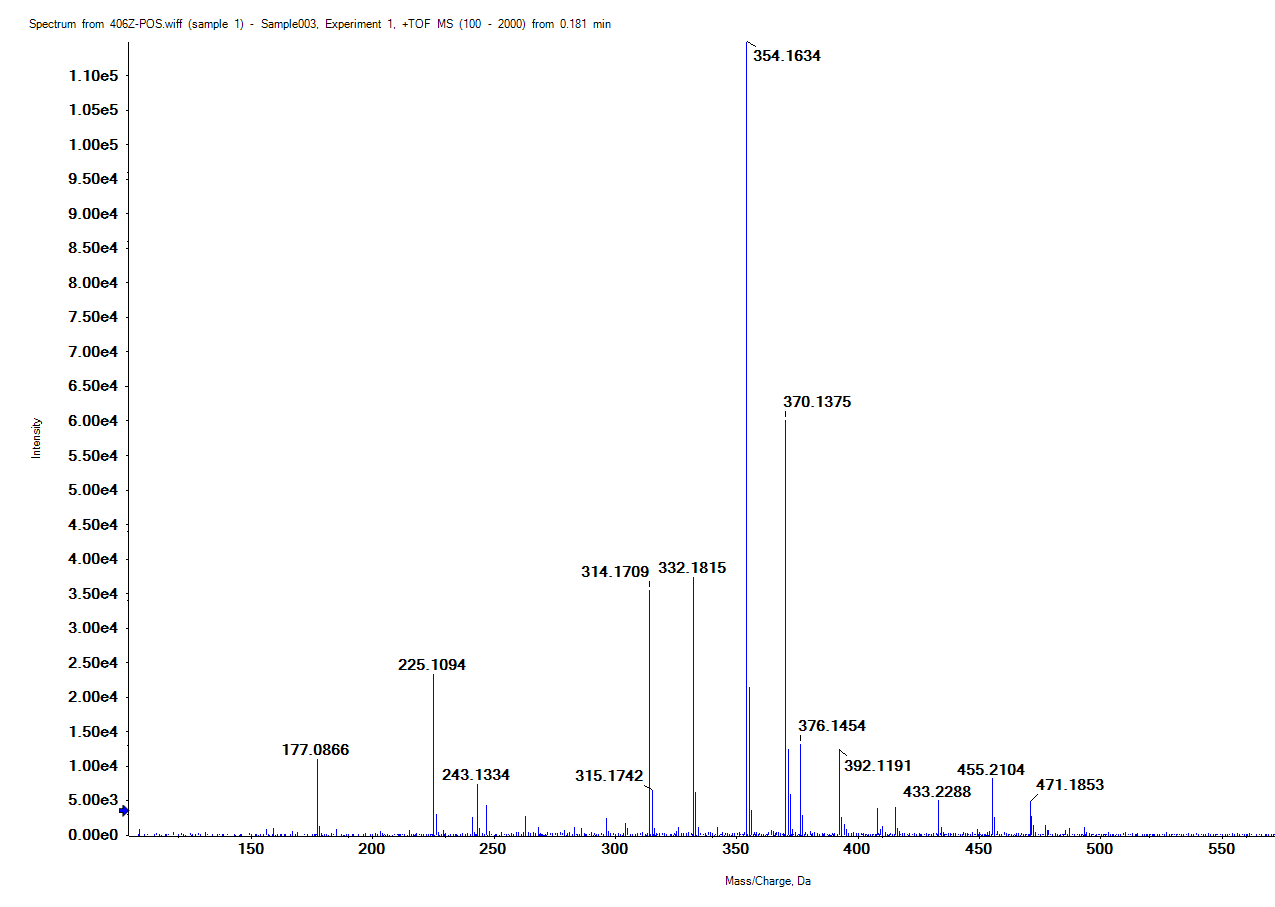


Figure S_47_. HPLC profile of amino acid-FDAAs of hydrolysates of compound **3**

**
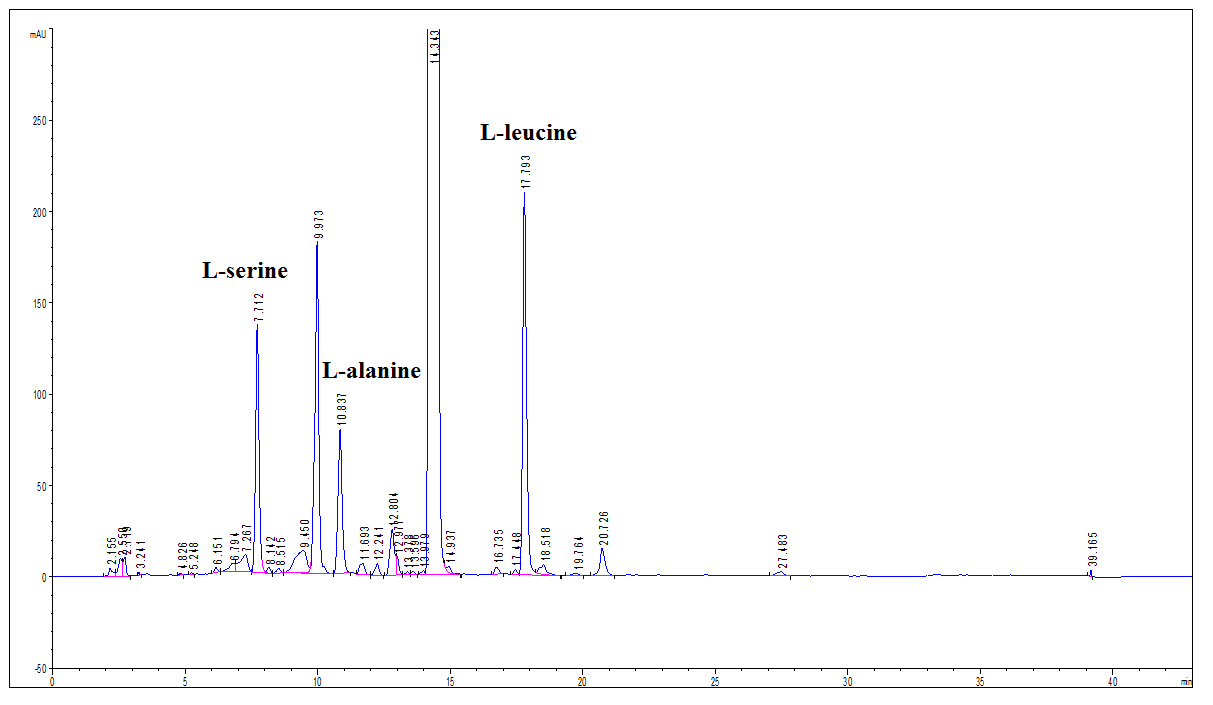
**

Figure S_48_. HPLC profile of amino acid-FDAAs of standard amino acids

**
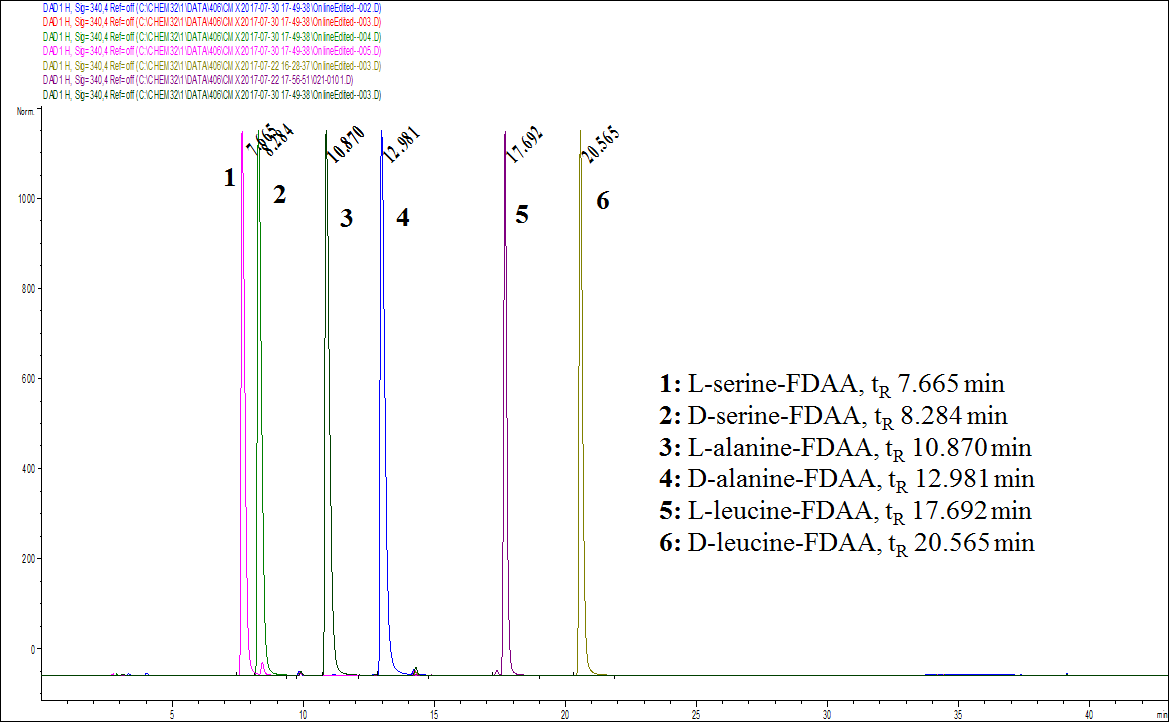
**

**Table S_5_. ^13^C and ^1^H NMR data for compound** 4

| No. | C | H (*J* = Hz) | No. | C | H (*J* = Hz) |
| --- | --- | --- | --- | --- | --- |
| 3 | 62.4, CH_2_ | 5.42, 2H, br q | 2′ | 17.7, CH_3_ | 2.18, 3H, s |
| 4 | 40.3, CH | 3.28, 1H, m | 1 | 175.3, C | − |
| 5 | 57.3, CH_2_ | 3.73, 2H, br s | 2 | 33.2, CH | 2.51, 1H, m |
| 6 | 168.2, C | − | 3 | 18.6, CH_3_ | 1.06, 6H, s |
| 1′ | 158.3, C | − | 4 | 18.6, CH_3_ | 1.06, 6H, s |

Figure S_49_. ^1^H-NMR spectrum of compound **4**

**
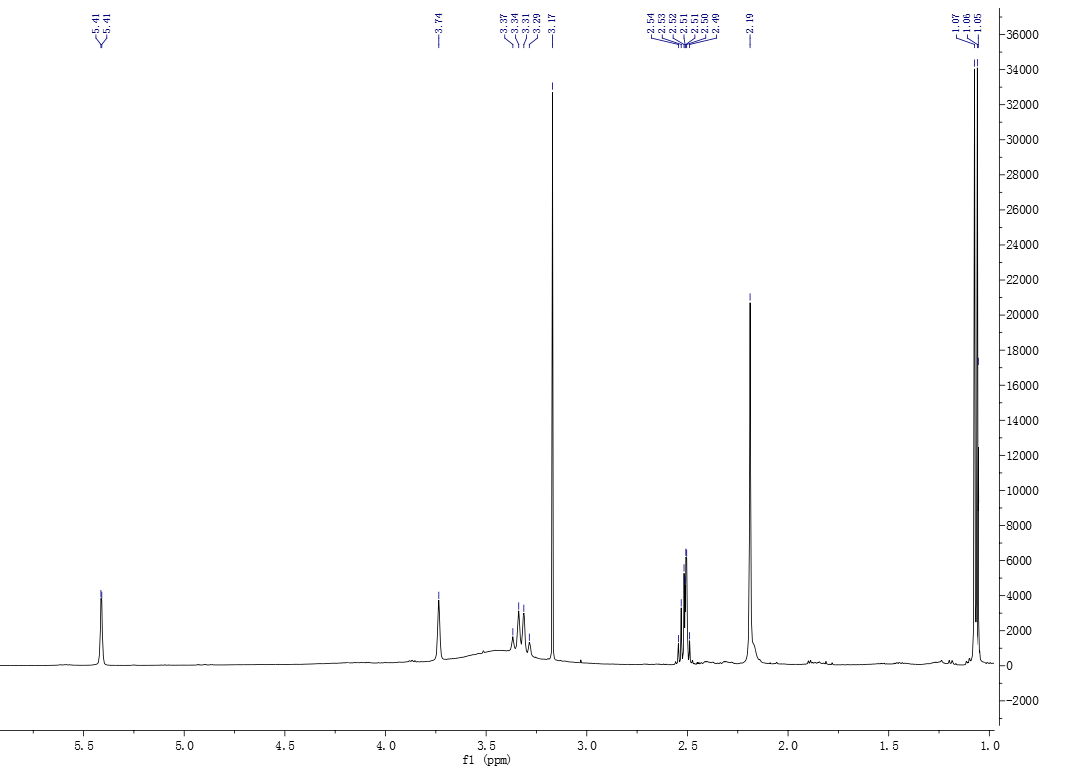
**

Figure S_50_. ^13^C-NMR spectrum of compound **4**

**
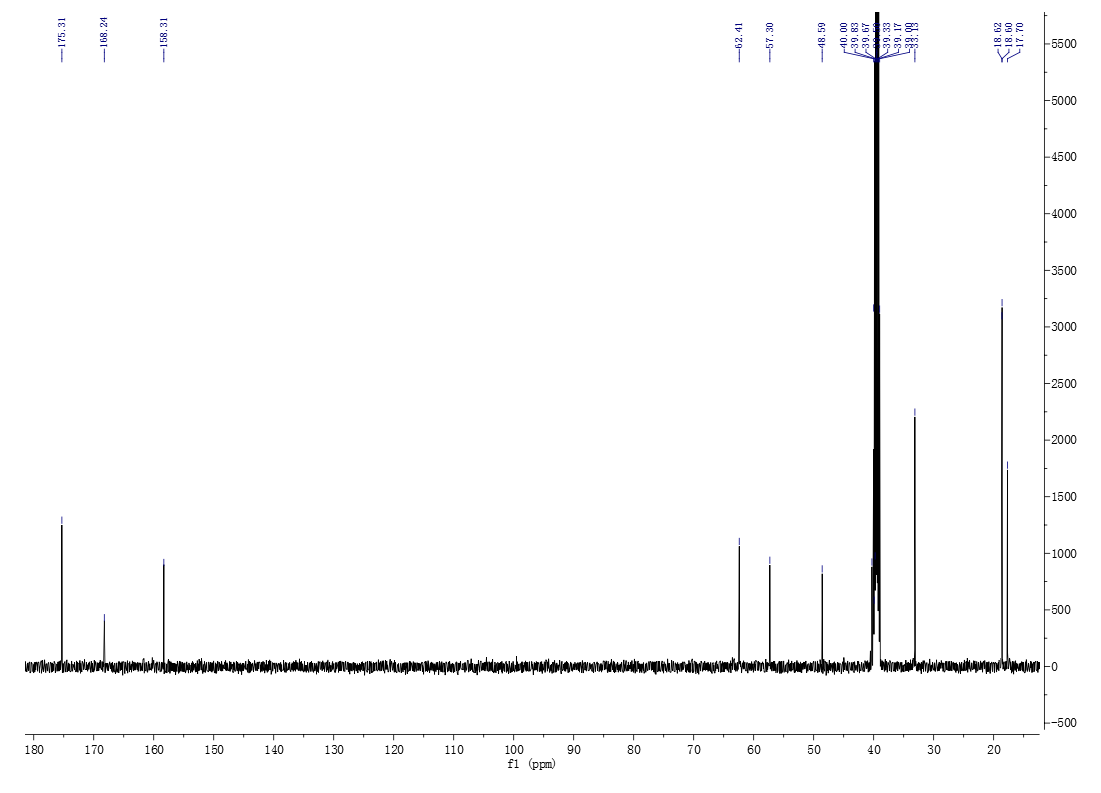
**

Figure S_51_. ^13^C-NMR spectrum of compound **4**

**
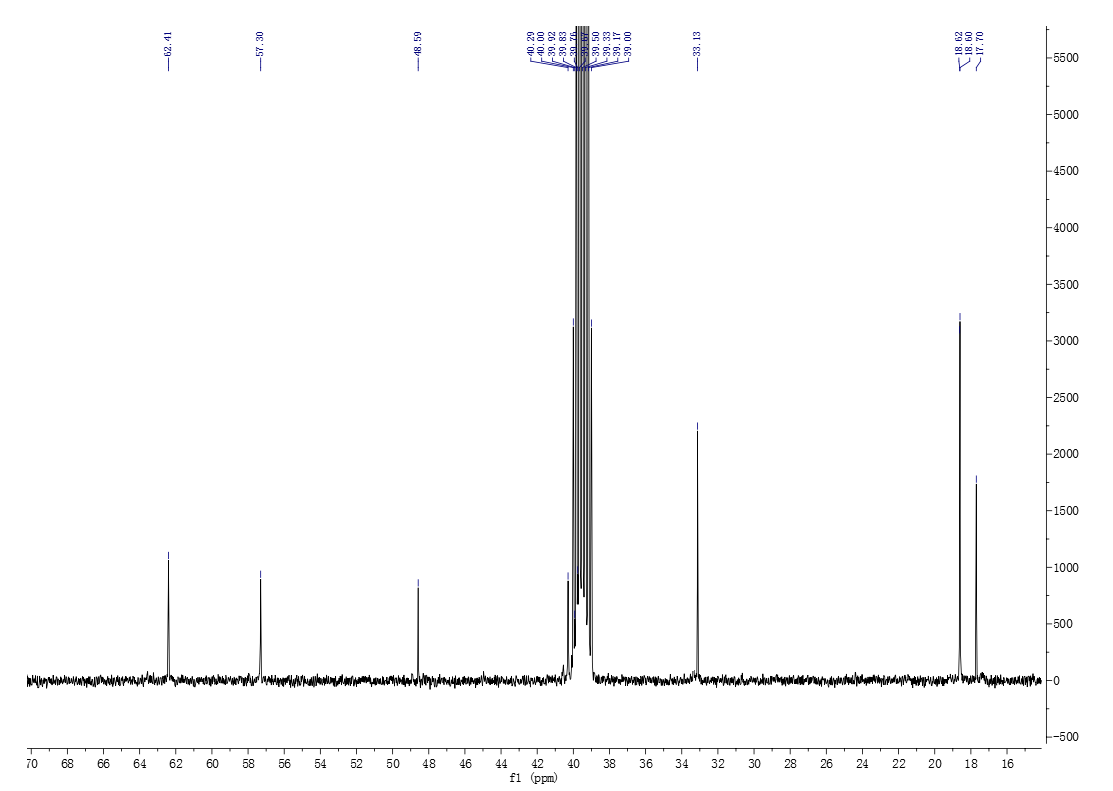
**

Figure S_52_. HSQC spectrum of compound **4**


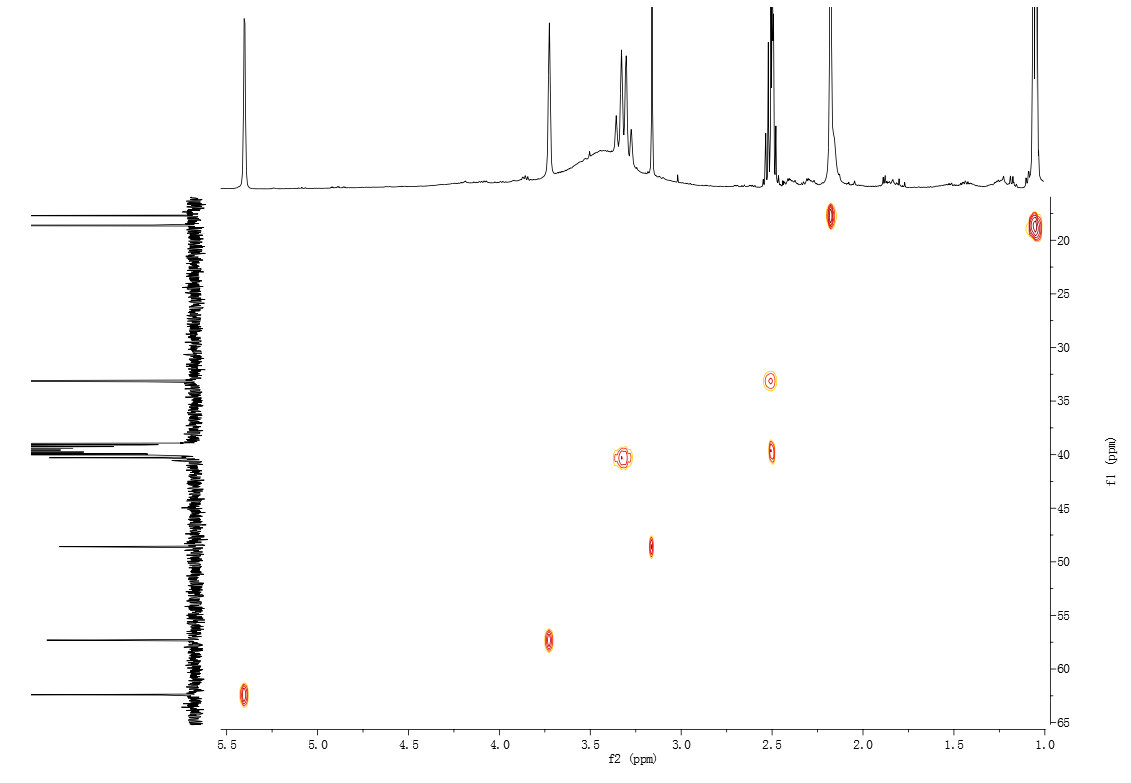


Figure S_53_. ^1^H-^1^H COSY spectrum of compound **4**

**
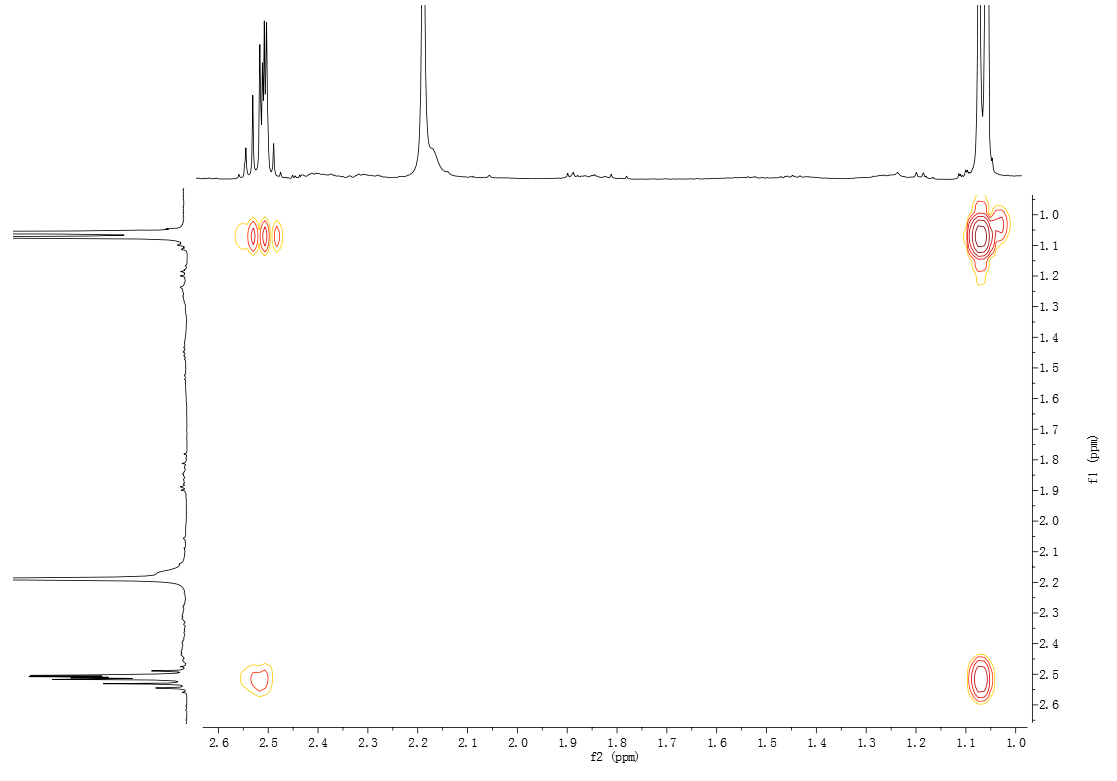
**

Figure S_54_. ^1^H-^1^H COSY spectrum of compound **4**

**
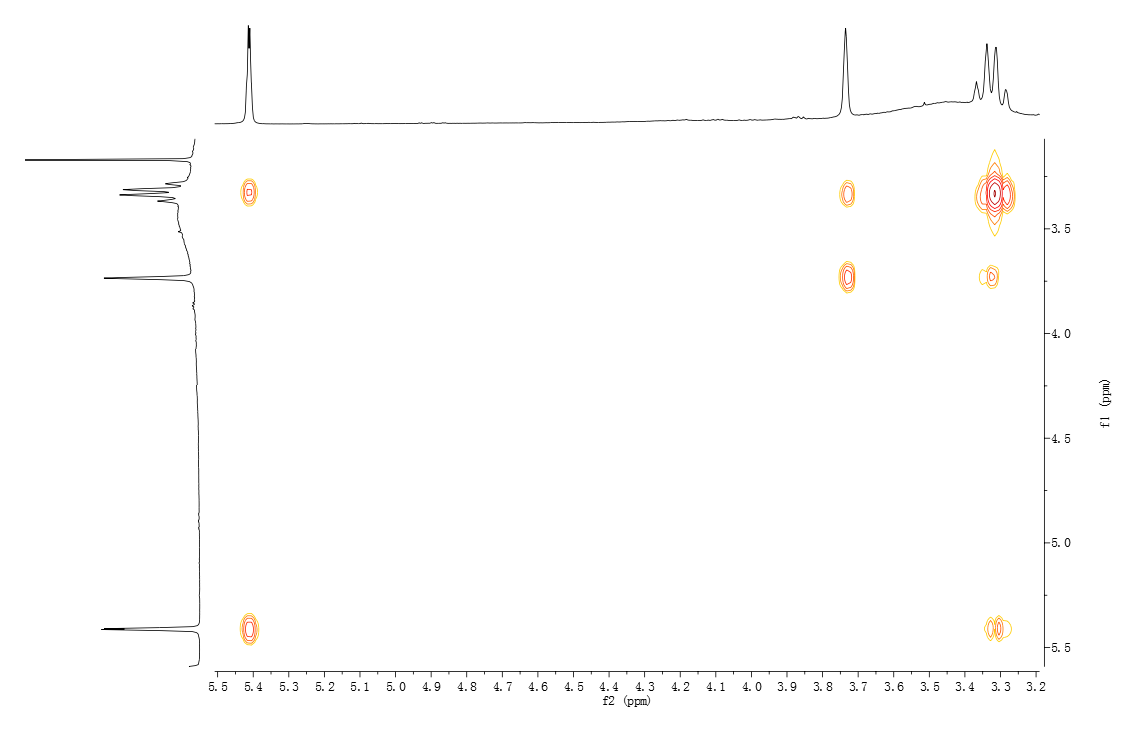
**

Figure S_55_. HMBC spectrum of compound **4**

**
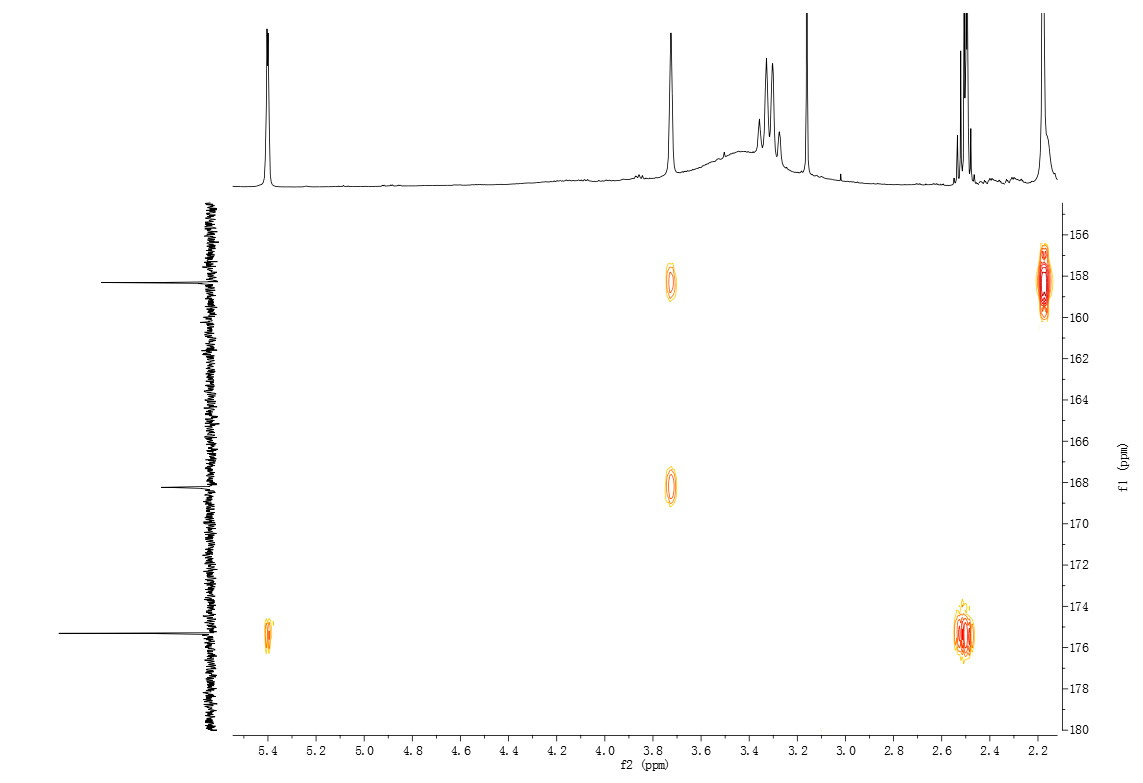
**

Figure S_56_. HMBC spectrum of compound **4**

**
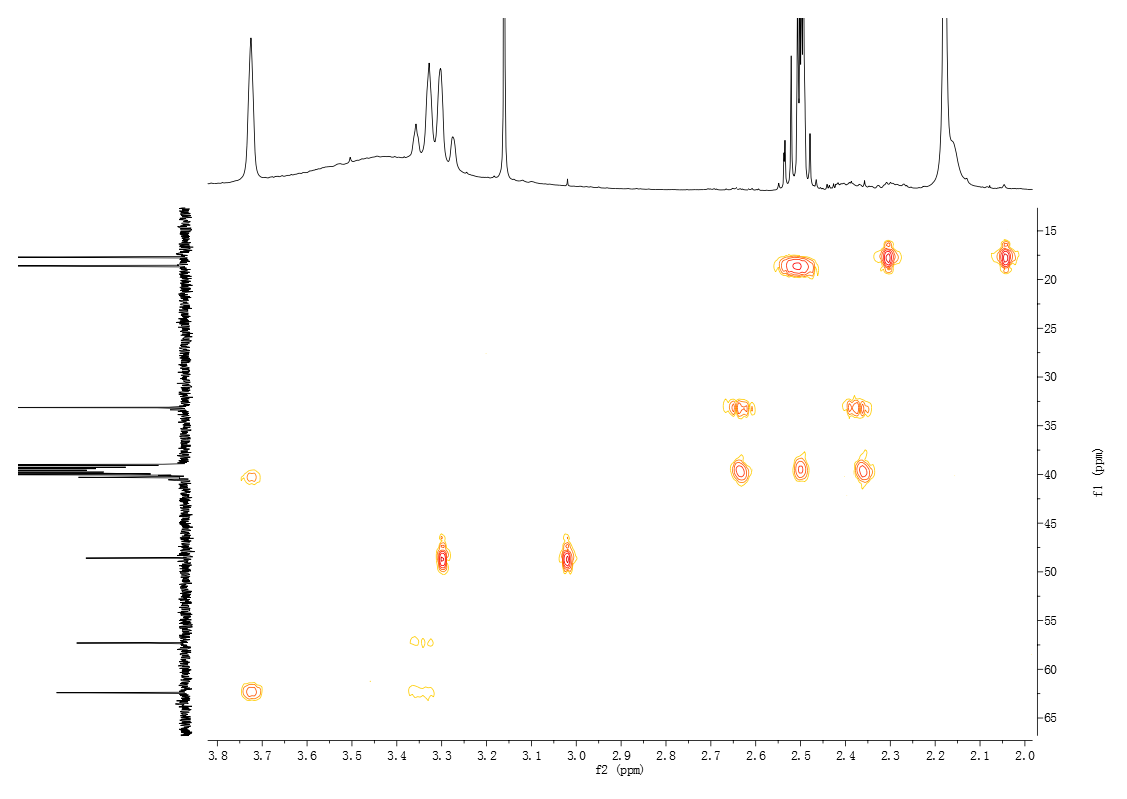
**

Figure S_57_. HMBC spectrum of compound **4**

**
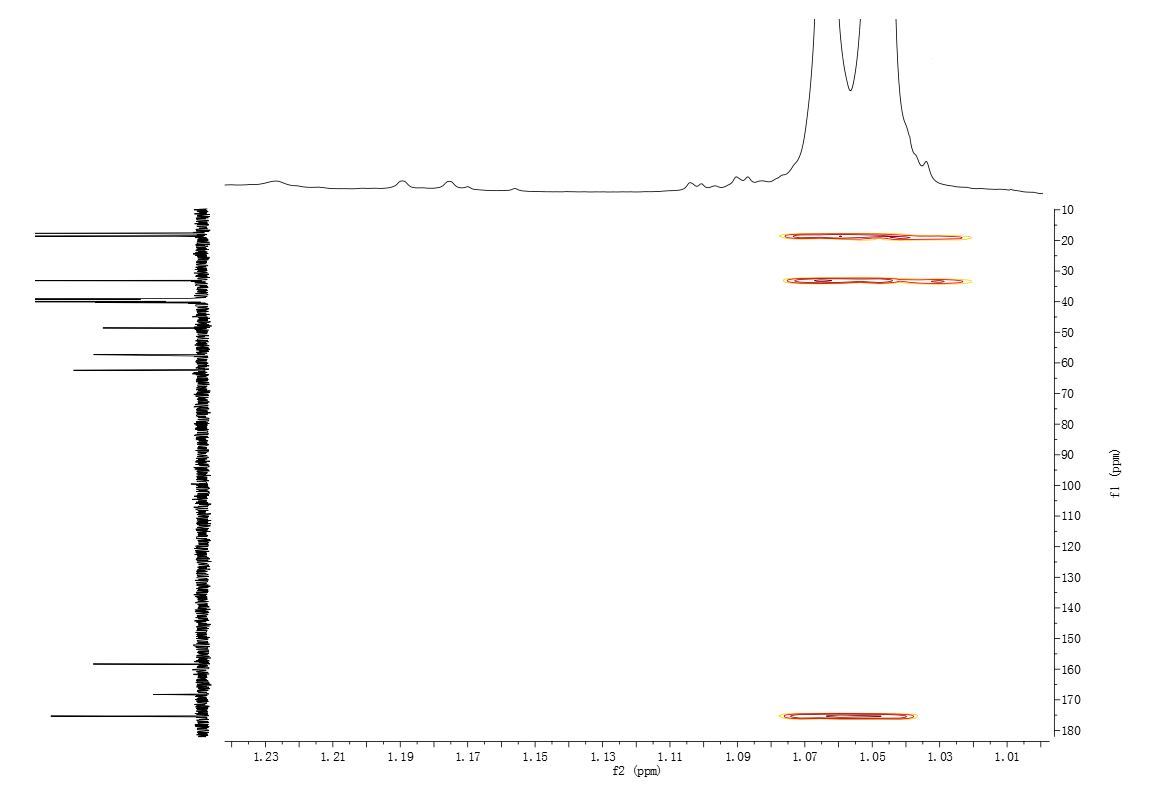
**

Figure S_58_. HRESIMS of compound **4**


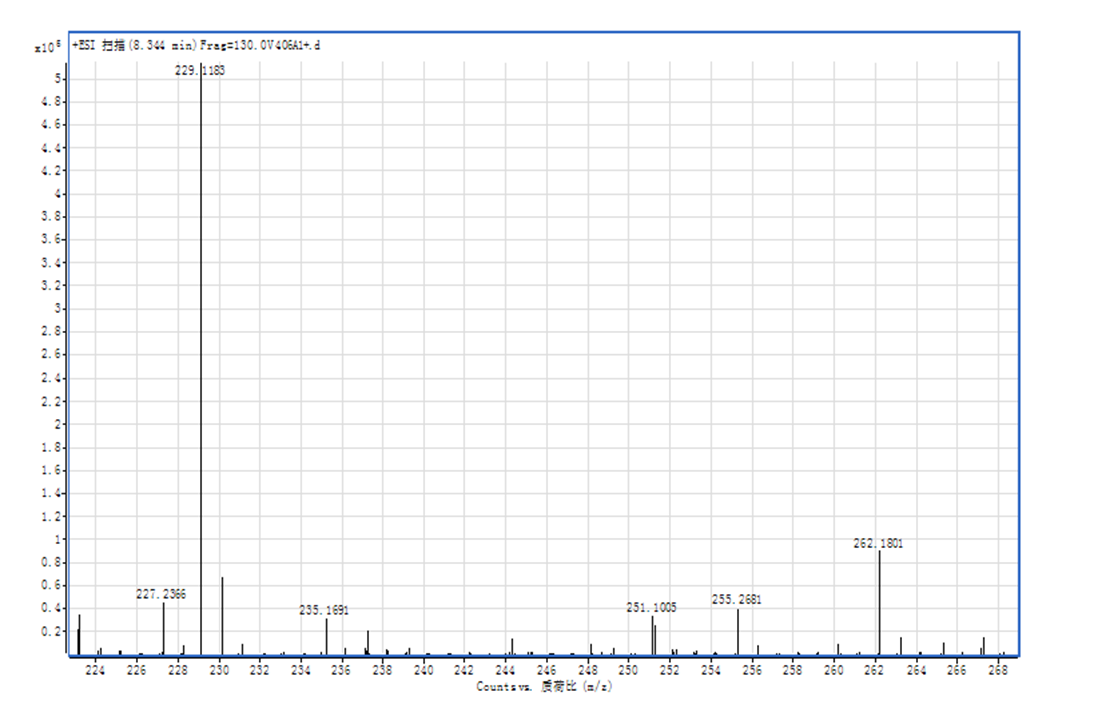


Figure S_59_. MS-MS spectrum of compound **4**

**Figure S_60_. The optimized conformers and equilibrium populations of compound 2**

| **2**-1C 64.73% | **2**-2C 11.13% | **2**-3C 7.13% |
| --- | --- | --- |
| 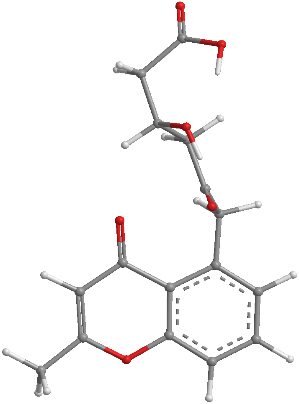 | 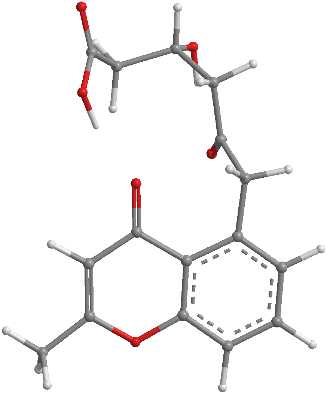 | 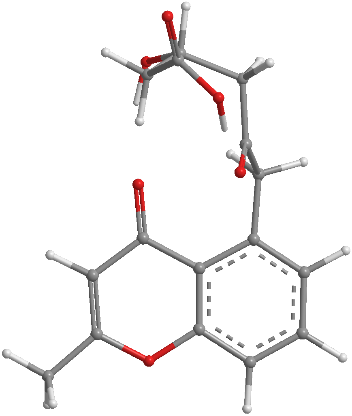 |
| **2**-4C 6.78% | **2**-5C 5.98% | **2**-6C 4.25% |
| 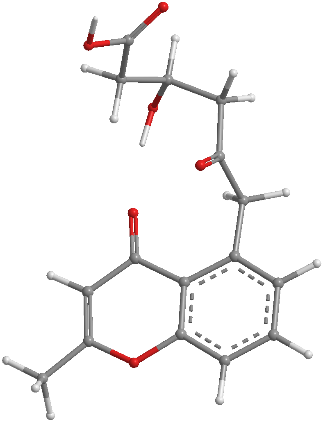 | 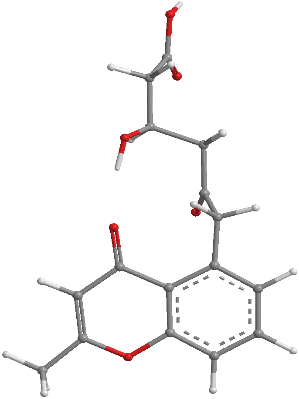 | 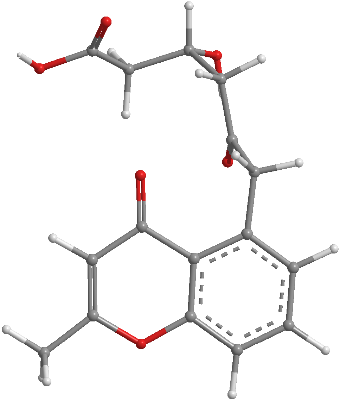 |

Table S_6_. Important thermodynamic parameters (a.u.) of the optimized **2** at the B3LYP/6- 31G(d) level in the gas phase.

| Conformers | *E* | *E′=E+ZPE* | *H* | *G* |
| --- | --- | --- | --- | --- |
| **2**-1C | -1070.428295 | -1070.448085 | -1070.427351 | -1070.498454 |
| **2**-2C | -1070.426107 | -1070.445834 | -1070.425163 | -1070.494974 |
| **2**-3C | -1070.425606 | -1070.445211 | -1070.424662 | -1070.494077 |
| **2**-4C | -1070.425653 | -1070.445794 | -1070.424709 | -1070.496561 |
| **2**-5C | -1070.446311 | -1070.425964 | -1070.425019 | -1070.498206 |
| **2**-6C | -1070.425392 | -1070.445604 | -1070.424448 | -1070.496476 |

*E*, *E*′, *H*, *G*: Total energy, total energy with zero point energy (ZPE), enthalpy, and Gibbs free.

Table S_7_. Excitation energies, oscillator strengths and rotatory strengths in the ECD spectra of **2**-1C

| Excited  State | Excitation  energies(eV) | λ ( nm) | Oscillator  strengths | Rotatory  strength* | Excited  State | Excitation  energies(eV) | λ ( nm) | Oscillator  strengths | Rotatory  strength* |
| --- | --- | --- | --- | --- | --- | --- | --- | --- | --- |
| **1** | 3.8162 | 324.89 | 0.0008 | -13.4176 | **31** | 6.3599 | 194.95 | 0.0034 | 6.6926 |
| **2** | 4.2321 | 292.96 | 0.0799 | 2.4547 | **32** | 6.3886 | 194.07 | 0.0083 | 8.8115 |
| **3** | 4.4476 | 278.76 | 0.0355 | 14.5348 | **33** | 6.4390 | 192.55 | 0.0039 | -2.6214 |
| **4** | 4.5101 | 274.90 | 0.0011 | 1.2467 | **34** | 6.4746 | 191.49 | 0.0045 | -0.4974 |
| **5** | 4.7059 | 263.47 | 0.0038 | -9.8688 | **35** | 6.5333 | 189.77 | 0.0001 | -0.9194 |
| **6** | 4.8412 | 256.10 | 0.0253 | -0.2375 | **36** | 6.5936 | 188.04 | 0.0002 | 0.2114 |
| **7** | 5.1375 | 241.33 | 0.0471 | -14.0616 | **37** | 6.6052 | 187.71 | 0.0117 | -16.4365 |
| **8** | 5.1849 | 239.12 | 0.0123 | -19.0869 | **38** | 6.6106 | 187.55 | 0.0159 | 20.9694 |
| **9** | 5.2172 | 237.65 | 0.0161 | -15.0171 | **39** | 6.6369 | 186.81 | 0.0807 | 20.6377 |
| **10** | 5.2765 | 234.98 | 0.1303 | -3.4183 | **40** | 6.6570 | 186.25 | 0.1317 | -9.3300 |
| **11** | 5.3484 | 231.81 | 0.0489 | 3.8760 | **41** | 6.6753 | 185.74 | 0.0062 | -3.4329 |
| **12** | 5.3676 | 230.99 | 0.0035 | 5.3673 | **42** | 6.6782 | 185.65 | 0.0337 | -13.3918 |
| **13** | 5.5267 | 224.33 | 0.0211 | -1.8809 | **43** | 6.6899 | 185.33 | 0.0141 | -9.9357 |
| **14** | 5.5433 | 223.66 | 0.0304 | 16.1325 | **44** | 6.6964 | 185.15 | 0.0389 | 1.0054 |
| **15** | 5.5589 | 223.04 | 0.0632 | 6.1223 | **45** | 6.7407 | 183.93 | 0.0061 | 6.4977 |
| **16** | 5.6303 | 220.21 | 0.0082 | 16.7060 | **46** | 6.7659 | 183.25 | 0.0007 | 1.6925 |
| **17** | 5.6743 | 218.50 | 0.0036 | 9.8622 | **47** | 6.7921 | 182.54 | 0.0017 | -7.5287 |
| **18** | 5.6840 | 218.13 | 0.0010 | 2.8064 | **48** | 6.8156 | 181.91 | 0.0036 | -4.8784 |
| **19** | 5.7040 | 217.36 | 0.0098 | 16.5545 | **49** | 6.8371 | 181.34 | 0.0029 | 5.9611 |
| **20** | 5.8767 | 210.98 | 0.0110 | -2.2935 | **50** | 6.8485 | 181.04 | 0.0093 | -3.0211 |
| **21** | 5.9028 | 210.04 | 0.0766 | -22.4047 | **51** | 6.8539 | 180.89 | 0.0305 | -10.4703 |
| **22** | 5.9339 | 208.94 | 0.0214 | -3.4010 | **52** | 6.8984 | 179.73 | 0.0282 | 28.0944 |
| **23** | 5.9715 | 207.63 | 0.0061 | 1.5311 | **53** | 6.9078 | 179.48 | 0.0097 | 2.3896 |
| **24** | 5.9962 | 206.77 | 0.0013 | 3.1681 | **54** | 6.9499 | 178.40 | 0.6415 | -103.5308 |
| **25** | 6.0684 | 204.31 | 0.1259 | 21.9668 | **55** | 6.9921 | 177.32 | 0.0219 | -9.8553 |
| **26** | 6.1537 | 201.48 | 0.0086 | 4.5963 | **56** | 7.0014 | 177.08 | 0.0110 | 9.2521 |
| **27** | 6.1851 | 200.46 | 0.0118 | 4.0598 | **57** | 7.0260 | 176.46 | 0.0052 | 10.7296 |
| **28** | 6.2306 | 198.99 | 0.0117 | 1.4256 | **58** | 7.0419 | 176.07 | 0.0012 | 2.7687 |
| **29** | 6.2828 | 197.34 | 0.0009 | 0.6443 | **59** | 7.0529 | 175.79 | 0.0875 | 49.6808 |
| **30** | 6.2983 | 196.85 | 0.0011 | 0.1297 | **60** | 7.0652 | 175.49 | 0.0078 | -3.9358 |

* R(velocity) 10**-40 erg-esu-cm.

Table S_8_. Excitation energies, oscillator strengths and rotatory strengths in the ECD spectra of **2**-2C

| Excited  State | Excitation  energies(eV) | λ  (nm) | Oscillator  strengths | Rotatory  strength* | Excited  State | Excitation  energies(eV) | λ  (nm) | Oscillator  strengths | Rotatory  strength* |
| --- | --- | --- | --- | --- | --- | --- | --- | --- | --- |
| **1** | 3.8725 | 320.17 | 0.0083 | 23.3115 | **31** | 6.1889 | 200.33 | 0.0045 | -0.6866 |
| **2** | 3.9163 | 316.58 | 0.0108 | 5.5293 | **32** | 6.1970 | 200.07 | 0.0027 | -0.5493 |
| **3** | 4.0834 | 303.63 | 0.0143 | -44.5133 | **33** | 6.2144 | 199.51 | 0.0061 | -10.3527 |
| **4** | 4.3181 | 287.13 | 0.0709 | 5.2180 | **34** | 6.2664 | 197.86 | 0.0013 | 4.0598 |
| **5** | 4.4458 | 278.88 | 0.0003 | -0.1979 | **35** | 6.3745 | 194.50 | 0.0065 | -3.2658 |
| **6** | 4.5017 | 275.41 | 0.0014 | 13.0493 | **36** | 6.4049 | 193.58 | 0.0042 | -4.1588 |
| **7** | 4.7704 | 259.91 | 0.0479 | -2.6770 | **37** | 6.4327 | 192.74 | 0.0014 | -1.0661 |
| **8** | 5.0147 | 247.24 | 0.0006 | -0.5102 | **38** | 6.4639 | 191.81 | 0.0025 | 3.1169 |
| **9** | 5.0432 | 245.85 | 0.0050 | 2.2115 | **39** | 6.4688 | 191.66 | 0.0006 | -0.8288 |
| **10** | 5.1296 | 241.70 | 0.0514 | 1.2550 | **40** | 6.5216 | 190.11 | 0.0165 | -4.2855 |
| **11** | 5.1833 | 239.20 | 0.0039 | 3.1910 | **41** | 6.5412 | 189.54 | 0.0012 | -1.5302 |
| **12** | 5.2042 | 238.24 | 0.0784 | -2.5704 | **42** | 6.6422 | 186.66 | 0.1840 | -5.5029 |
| **13** | 5.3434 | 232.03 | 0.1879 | 5.1700 | **43** | 6.6697 | 185.89 | 0.0067 | -6.5399 |
| **14** | 5.4611 | 227.03 | 0.0004 | -1.2909 | **44** | 6.6795 | 185.62 | 0.0082 | -8.0065 |
| **15** | 5.4691 | 226.70 | 0.0072 | 14.4038 | **45** | 6.6824 | 185.54 | 0.0133 | 11.6283 |
| **16** | 5.5064 | 225.17 | 0.0290 | 27.4779 | **46** | 6.6929 | 185.25 | 0.0248 | 37.4429 |
| **17** | 5.5336 | 224.06 | 0.0011 | 4.9147 | **47** | 6.6968 | 185.14 | 0.0108 | -3.1125 |
| **18** | 5.5518 | 223.32 | 0.0076 | -3.0950 | **48** | 6.7063 | 184.88 | 0.0125 | 7.5236 |
| **19** | 5.5721 | 222.51 | 0.0374 | -14.6885 | **49** | 6.7121 | 184.72 | 0.0005 | 2.6838 |
| **20** | 5.6457 | 219.61 | 0.0032 | -3.7814 | **50** | 6.7191 | 184.52 | 0.0064 | -0.7519 |
| **21** | 5.7088 | 217.18 | 0.0259 | -15.4799 | **51** | 6.7723 | 183.08 | 0.0026 | -0.5646 |
| **22** | 5.8421 | 212.23 | 0.0002 | 0.6503 | **52** | 6.7887 | 182.63 | 0.0068 | -11.8153 |
| **23** | 5.9383 | 208.79 | 0.0016 | 1.4962 | **53** | 6.8165 | 181.89 | 0.0115 | -1.0213 |
| **24** | 5.9611 | 207.99 | 0.0112 | 15.4280 | **54** | 6.8364 | 181.36 | 0.0213 | 8.5903 |
| **25** | 5.9706 | 207.66 | 0.0035 | -8.6012 | **55** | 6.8530 | 180.92 | 0.0020 | -15.7487 |
| **26** | 6.0026 | 206.55 | 0.0286 | -0.2394 | **56** | 6.8869 | 180.03 | 0.0293 | -3.1501 |
| **27** | 6.0487 | 204.98 | 0.1062 | 3.0341 | **57** | 6.8943 | 179.84 | 0.0097 | -4.9445 |
| **28** | 6.0779 | 203.99 | 0.0413 | 2.1110 | **58** | 6.9265 | 179.00 | 0.1170 | -45.3188 |
| **29** | 6.1344 | 202.11 | 0.0245 | -14.8399 | **59** | 6.9406 | 178.64 | 0.4004 | 86.6791 |
| **30** | 6.1868 | 200.40 | 0.0032 | 3.2186 | **60** | 6.9532 | 178.31 | 0.1251 | 25.2578 |

* R(velocity) 10**-40 erg-esu-cm

Table S_9_. Excitation energies, oscillator strengths, and rotatory strengths in the ECD spectra of **2**-3C

| Excited  State | Excitation  energies(eV) | λ  (nm) | Oscillator  strengths | Rotatory  strength* | Excited  State | Excitation  energies(eV) | λ  (nm) | Oscillator  strengths | Rotatory  strength* |
| --- | --- | --- | --- | --- | --- | --- | --- | --- | --- |
| **1** | 3.8746 | 320.00 | 0.0001 | -0.1351 | **31** | 6.2425 | 198.61 | 0.0093 | 2.1319 |
| **2** | 4.1045 | 302.07 | 0.0087 | 8.0195 | **32** | 6.2618 | 198.00 | 0.0002 | 18.7237 |
| **3** | 4.2164 | 294.05 | 0.0805 | -44.7040 | **33** | 6.3644 | 194.81 | 0.0389 | -0.8292 |
| **4** | 4.4156 | 280.79 | 0.0218 | 38.4770 | **34** | 6.4377 | 192.59 | 0.0071 | -12.3505 |
| **5** | 4.5827 | 270.55 | 0.0012 | -6.3881 | **35** | 6.4393 | 192.54 | 0.0003 | 4.0963 |
| **6** | 4.7654 | 260.18 | 0.0201 | 6.1293 | **36** | 6.4487 | 192.26 | 0.0035 | -0.5750 |
| **7** | 4.8286 | 256.77 | 0.0222 | -9.8016 | **37** | 6.4826 | 191.26 | 0.0070 | -11.9241 |
| **8** | 4.8667 | 254.76 | 0.0071 | -10.9844 | **38** | 6.5334 | 189.77 | 0.0027 | 10.4357 |
| **9** | 5.1370 | 241.36 | 0.0365 | -46.4912 | **39** | 6.5413 | 189.54 | 0.0210 | 8.8683 |
| **10** | 5.1661 | 240.00 | 0.0299 | 15.5745 | **40** | 6.6297 | 187.01 | 0.0554 | 20.0873 |
| **11** | 5.2190 | 237.56 | 0.0108 | 7.3098 | **41** | 6.6531 | 186.36 | 0.1718 | 1.8212 |
| **12** | 5.2753 | 235.03 | 0.0228 | -2.7415 | **42** | 6.6988 | 185.09 | 0.0099 | 2.6559 |
| **13** | 5.3263 | 232.78 | 0.1124 | -19.5297 | **43** | 6.7105 | 184.76 | 0.0111 | -1.9005 |
| **14** | 5.3317 | 232.54 | 0.0009 | -1.2490 | **44** | 6.7228 | 184.42 | 0.0307 | -20.1777 |
| **15** | 5.4272 | 228.45 | 0.0953 | 37.6228 | **45** | 6.7768 | 182.95 | 0.0007 | -15.2731 |
| **16** | 5.4639 | 226.92 | 0.0022 | 23.6235 | **46** | 6.7841 | 182.76 | 0.0084 | 0.9101 |
| **17** | 5.5066 | 225.15 | 0.0420 | -6.5785 | **47** | 6.7859 | 182.71 | 0.0047 | 23.6313 |
| **18** | 5.5790 | 222.23 | 0.0148 | 9.3187 | **48** | 6.8250 | 181.66 | 0.0021 | -8.1543 |
| **19** | 5.6106 | 220.98 | 0.0007 | 2.4650 | **49** | 6.8406 | 181.25 | 0.0087 | 3.0207 |
| **20** | 5.6514 | 219.39 | 0.0047 | -0.0872 | **50** | 6.8415 | 181.22 | 0.0083 | 2.6007 |
| **21** | 5.7397 | 216.01 | 0.0625 | -15.5914 | **51** | 6.8590 | 180.76 | 0.0082 | 5.7929 |
| **22** | 5.7633 | 215.13 | 0.0007 | 0.0072 | **52** | 6.8823 | 180.15 | 0.0032 | -19.1202 |
| **23** | 5.8496 | 211.95 | 0.0009 | 0.7899 | **53** | 6.9011 | 179.66 | 0.0898 | -7.2892 |
| **24** | 5.9015 | 210.09 | 0.0404 | -19.0850 | **54** | 6.9398 | 178.66 | 0.1151 | 31.6268 |
| **25** | 5.9588 | 208.07 | 0.0029 | 2.9528 | **55** | 6.9442 | 178.54 | 0.2834 | -53.3393 |
| **26** | 5.9856 | 207.14 | 0.0072 | 0.1623 | **56** | 6.9647 | 178.02 | 0.0372 | 22.4141 |
| **27** | 6.0276 | 205.69 | 0.0018 | -0.6191 | **57** | 6.9701 | 177.88 | 0.0292 | 16.1012 |
| **28** | 6.0530 | 204.83 | 0.0066 | 7.0356 | **58** | 6.9869 | 177.45 | 0.2013 | -1.8603 |
| **29** | 6.0561 | 204.73 | 0.0075 | -2.7307 | **59** | 7.0053 | 176.99 | 0.0005 | -43.7987 |
| **30** | 6.0949 | 203.42 | 0.1024 | 16.2811 | **60** | 6.2425 | 198.61 | 0.0093 | 1.0292 |

* R(velocity) 10**-40 erg-esu-cm.

Table S_10_. Excitation energies, oscillator strengths and rotatory strengths in the ECD spectra of **2**-4C

| Excited  State | Excitation  energies(eV) | λ  (nm) | Oscillator  strengths | Rotatory  strength* | Excited  State | Excitation  energies(eV) | λ  (nm) | Oscillator  strengths | Rotatory  strength* |
| --- | --- | --- | --- | --- | --- | --- | --- | --- | --- |
| **1** | 3.8433 | 322.60 | 0.0054 | -34.6006 | **31** | 6.2674 | 197.83 | 0.0003 | 0.1567 |
| **2** | 3.9018 | 317.76 | 0.0279 | 28.7806 | **32** | 6.2914 | 197.07 | 0.0007 | -2.8071 |
| **3** | 4.3208 | 286.95 | 0.0371 | 57.5748 | **33** | 6.2944 | 196.97 | 0.0036 | 2.7895 |
| **4** | 4.3838 | 282.83 | 0.0348 | -52.7935 | **34** | 6.3068 | 196.59 | 0.0107 | 0.9657 |
| **5** | 4.5842 | 270.46 | 0.0052 | -4.4000 | **35** | 6.3854 | 194.17 | 0.0009 | -3.5172 |
| **6** | 4.7982 | 258.40 | 0.0244 | 4.0210 | **36** | 6.4304 | 192.81 | 0.0011 | 0.2509 |
| **7** | 5.0567 | 245.19 | 0.0431 | -14.4075 | **37** | 6.4862 | 191.15 | 0.0024 | -1.5290 |
| **8** | 5.0966 | 243.27 | 0.0060 | 0.3352 | **38** | 6.5303 | 189.86 | 0.0532 | 5.4919 |
| **9** | 5.2161 | 237.70 | 0.0572 | -6.0128 | **39** | 6.5401 | 189.57 | 0.0107 | 3.0183 |
| **10** | 5.2597 | 235.73 | 0.0316 | -13.8913 | **40** | 6.5512 | 189.25 | 0.0032 | 0.0405 |
| **11** | 5.3281 | 232.70 | 0.1150 | 60.2941 | **41** | 6.5693 | 188.73 | 0.0061 | 7.9727 |
| **12** | 5.4292 | 228.37 | 0.1128 | -61.5527 | **42** | 6.6058 | 187.69 | 0.0092 | 0.5605 |
| **13** | 5.5748 | 222.40 | 0.0036 | -4.5490 | **43** | 6.6128 | 187.49 | 0.0021 | -3.1406 |
| **14** | 5.6218 | 220.54 | 0.0159 | 24.9094 | **44** | 6.6418 | 186.67 | 0.0303 | 3.2283 |
| **15** | 5.6813 | 218.23 | 0.0245 | 7.1808 | **45** | 6.6583 | 186.21 | 0.0470 | 38.3501 |
| **16** | 5.7372 | 216.11 | 0.0372 | -6.4205 | **46** | 6.6709 | 185.86 | 0.0603 | 16.6237 |
| **17** | 5.7669 | 214.99 | 0.0040 | 6.6581 | **47** | 6.6966 | 185.15 | 0.0568 | -63.9923 |
| **18** | 5.7774 | 214.60 | 0.0019 | 8.4648 | **48** | 6.7024 | 184.99 | 0.0110 | -22.9800 |
| **19** | 5.7858 | 214.29 | 0.0012 | -12.0486 | **49** | 6.7298 | 184.23 | 0.0106 | -27.1058 |
| **20** | 5.8732 | 211.10 | 0.0077 | -5.1002 | **50** | 6.7549 | 183.55 | 0.0125 | -5.9246 |
| **21** | 5.9287 | 209.12 | 0.0006 | 0.3740 | **51** | 6.7750 | 183.00 | 0.0195 | -10.1554 |
| **22** | 5.9716 | 207.62 | 0.0010 | -2.3507 | **52** | 6.7865 | 182.69 | 0.0290 | 18.2468 |
| **23** | 6.0120 | 206.23 | 0.0003 | 0.1578 | **53** | 6.7969 | 182.41 | 0.0128 | 9.4532 |
| **24** | 6.0361 | 205.40 | 0.1437 | 6.1007 | **54** | 6.8139 | 181.96 | 0.0047 | -2.6712 |
| **25** | 6.0568 | 204.70 | 0.0046 | 0.1977 | **55** | 6.8228 | 181.72 | 0.0099 | 12.7562 |
| **26** | 6.1125 | 202.84 | 0.0205 | -3.5397 | **56** | 6.8539 | 180.90 | 0.0037 | -0.0737 |
| **27** | 6.1220 | 202.52 | 0.0037 | 8.9294 | **57** | 6.8860 | 180.05 | 0.0757 | 12.0513 |
| **28** | 6.1510 | 201.57 | 0.0173 | 2.9467 | **58** | 6.8973 | 179.76 | 0.0132 | -5.4028 |
| **29** | 6.1883 | 200.35 | 0.0062 | 1.8737 | **59** | 6.9273 | 178.98 | 0.0636 | -1.0638 |
| **30** | 6.2237 | 199.21 | 0.0164 | 12.6093 | **60** | 6.9346 | 178.79 | 0.0011 | -7.4208 |

* R(velocity) 10**-40 erg-esu-cm.

Table S_11_. Excitation energies, oscillator strengths and rotatory strengths in the ECD spectra of **2**-5C

| Excited  State | Excitation  Energies (eV) | λ  (nm) | Oscillator  strengths | Rotatory  strength* | Excited  State | Excitation  Energies (eV) | λ  (nm) | Oscillator  strengths | Rotatory  strength* |
| --- | --- | --- | --- | --- | --- | --- | --- | --- | --- |
| **1** | 3.8436 | 322.57 | 0.0029 | 26.1896 | **31** | 6.2737 | 197.62 | 0.0043 | 1.1670 |
| **2** | 3.9192 | 316.35 | 0.0383 | -16.1918 | **32** | 6.2849 | 197.27 | 0.0037 | 1.0359 |
| **3** | 4.3406 | 285.64 | 0.0411 | -64.1082 | **33** | 6.3409 | 195.53 | 0.0019 | 5.6482 |
| **4** | 4.4032 | 281.58 | 0.0289 | 56.4461 | **34** | 6.3467 | 195.35 | 0.0016 | 0.2975 |
| **5** | 4.6181 | 268.47 | 0.0049 | 4.6187 | **35** | 6.4129 | 193.34 | 0.0003 | -0.0645 |
| **6** | 4.8056 | 258.00 | 0.0228 | -3.8760 | **36** | 6.4749 | 191.48 | 0.0045 | 13.6675 |
| **7** | 5.0731 | 244.40 | 0.0510 | 16.1594 | **37** | 6.4975 | 190.82 | 0.0073 | -4.6358 |
| **8** | 5.2130 | 237.84 | 0.0542 | 2.8750 | **38** | 6.5244 | 190.03 | 0.0176 | -8.6288 |
| **9** | 5.2491 | 236.20 | 0.0320 | 9.3684 | **39** | 6.5306 | 189.85 | 0.0018 | -0.2888 |
| **10** | 5.3317 | 232.54 | 0.1164 | -53.5320 | **40** | 6.5495 | 189.30 | 0.0472 | -18.2301 |
| **11** | 5.3447 | 231.98 | 0.0003 | -1.0440 | **41** | 6.5621 | 188.94 | 0.0116 | 8.1498 |
| **12** | 5.4366 | 228.05 | 0.1184 | 55.5305 | **42** | 6.5763 | 188.53 | 0.0044 | -4.6334 |
| **13** | 5.6020 | 221.32 | 0.0022 | 5.2603 | **43** | 6.5917 | 188.09 | 0.0133 | 9.0411 |
| **14** | 5.6411 | 219.79 | 0.0129 | -22.2473 | **44** | 6.5991 | 187.88 | 0.0014 | -0.9782 |
| **15** | 5.6925 | 217.80 | 0.0245 | -3.3345 | **45** | 6.6323 | 186.94 | 0.0185 | -5.3638 |
| **16** | 5.7374 | 216.10 | 0.0003 | 5.4371 | **46** | 6.6608 | 186.14 | 0.0056 | -0.8530 |
| **17** | 5.7419 | 215.93 | 0.0377 | 10.9207 | **47** | 6.6654 | 186.01 | 0.0765 | -7.9394 |
| **18** | 5.7492 | 215.66 | 0.0078 | -7.9566 | **48** | 6.6837 | 185.50 | 0.0886 | 12.0617 |
| **19** | 5.7844 | 214.34 | 0.0059 | -5.6129 | **49** | 6.7117 | 184.73 | 0.0014 | 5.8780 |
| **20** | 5.8997 | 210.15 | 0.0108 | 5.2003 | **50** | 6.7259 | 184.34 | 0.0158 | 15.1908 |
| **21** | 5.9079 | 209.86 | 0.0017 | 0.9213 | **51** | 6.7651 | 183.27 | 0.0088 | -15.9608 |
| **22** | 5.9540 | 208.24 | 0.0031 | 1.9480 | **52** | 6.7751 | 183.00 | 0.0323 | 0.4831 |
| **23** | 6.0392 | 205.30 | 0.1564 | 0.1187 | **53** | 6.7879 | 182.65 | 0.0045 | -9.2928 |
| **24** | 6.0555 | 204.75 | 0.0084 | 1.7439 | **54** | 6.8198 | 181.80 | 0.0249 | -6.9314 |
| **25** | 6.0687 | 204.30 | 0.0049 | -5.1114 | **55** | 6.8261 | 181.63 | 0.0205 | 6.8616 |
| **26** | 6.1263 | 202.38 | 0.0130 | -6.0923 | **56** | 6.8392 | 181.28 | 0.0049 | 9.4984 |
| **27** | 6.1390 | 201.96 | 0.0031 | -8.5934 | **57** | 6.8439 | 181.16 | 0.0126 | -12.9889 |
| **28** | 6.1752 | 200.78 | 0.0089 | -2.9453 | **58** | 6.8480 | 181.05 | 0.0034 | -3.1049 |
| **29** | 6.2301 | 199.01 | 0.0174 | -7.5969 | **59** | 6.8817 | 180.17 | 0.0096 | 0.5423 |
| **30** | 6.2423 | 198.62 | 0.0021 | 0.4701 | **60** | 6.8911 | 179.92 | 0.0046 | 9.1331 |

* R(velocity) 10**-40 erg-esu-cm.

Table S_12_. Excitation energies, oscillator strengths and rotatory strengths in the ECD spectra of **2**-6C

| Excited  State | Excitation  Energies (eV) | λ  (nm) | Oscillator  strengths | Rotatory  strength* | Excited  State | Excitation  Energies (eV) | λ  (nm) | Oscillator  strengths | Rotatory  strength* |
| --- | --- | --- | --- | --- | --- | --- | --- | --- | --- |
| **1** | 3.7732 | 328.59 | 0.0008 | 12.9831 | **31** | 6.2552 | 198.21 | 0.0070 | 0.6191 |
| **2** | 4.1098 | 301.68 | 0.0349 | -10.4789 | **32** | 6.2756 | 197.57 | 0.0013 | -1.5390 |
| **3** | 4.3962 | 282.03 | 0.0725 | -14.3588 | **33** | 6.2855 | 197.25 | 0.0016 | -3.2636 |
| **4** | 4.5622 | 271.76 | 0.0032 | 16.0486 | **34** | 6.3313 | 195.83 | 0.0007 | 3.5215 |
| **5** | 4.7107 | 263.20 | 0.0003 | 0.0462 | **35** | 6.4209 | 193.09 | 0.0108 | 6.1275 |
| **6** | 4.8448 | 255.91 | 0.0231 | -1.4085 | **36** | 6.4251 | 192.97 | 0.0022 | 1.9663 |
| **7** | 5.0941 | 243.39 | 0.0067 | -5.8317 | **37** | 6.4534 | 192.12 | 0.0099 | -4.9796 |
| **8** | 5.1029 | 242.97 | 0.0007 | -0.9254 | **38** | 6.4717 | 191.58 | 0.0032 | -2.9353 |
| **9** | 5.1905 | 238.87 | 0.0658 | 36.8105 | **39** | 6.4830 | 191.24 | 0.0023 | -1.8126 |
| **10** | 5.2875 | 234.49 | 0.1584 | 10.6182 | **40** | 6.5056 | 190.58 | 0.0090 | 13.3198 |
| **11** | 5.3435 | 232.03 | 0.0409 | -4.0726 | **41** | 6.5127 | 190.37 | 0.0114 | -10.6157 |
| **12** | 5.4529 | 227.37 | 0.0010 | -10.1346 | **42** | 6.5387 | 189.62 | 0.0009 | -1.2363 |
| **13** | 5.5199 | 224.61 | 0.0254 | 16.6772 | **43** | 6.5666 | 188.81 | 0.0004 | -1.5424 |
| **14** | 5.5636 | 222.85 | 0.0037 | -5.6389 | **44** | 6.5824 | 188.36 | 0.0018 | -1.6510 |
| **15** | 5.5745 | 222.41 | 0.0410 | -23.7999 | **45** | 6.6095 | 187.58 | 0.0021 | -11.6081 |
| **16** | 5.6841 | 218.12 | 0.0327 | -34.6696 | **46** | 6.6427 | 186.65 | 0.0557 | 37.2379 |
| **17** | 5.7755 | 214.67 | 0.0332 | 17.4044 | **47** | 6.6585 | 186.20 | 0.1757 | -19.5865 |
| **18** | 5.7841 | 214.35 | 0.0049 | -9.4838 | **48** | 6.6660 | 186.00 | 0.0244 | 24.4719 |
| **19** | 5.7873 | 214.24 | 0.0011 | 0.4894 | **49** | 6.6718 | 185.83 | 0.0055 | 9.1454 |
| **20** | 5.7901 | 214.13 | 0.0024 | 6.0959 | **50** | 6.6939 | 185.22 | 0.0537 | -44.0645 |
| **21** | 5.9907 | 206.96 | 0.1152 | 18.8950 | **51** | 6.7428 | 183.88 | 0.0050 | -7.7197 |
| **22** | 6.0181 | 206.02 | 0.0239 | -12.5926 | **52** | 6.7631 | 183.32 | 0.0032 | 5.5146 |
| **23** | 6.0743 | 204.11 | 0.0019 | 0.7323 | **53** | 6.7743 | 183.02 | 0.0024 | 10.1961 |
| **24** | 6.0856 | 203.73 | 0.0680 | -17.8403 | **54** | 6.7782 | 182.92 | 0.0060 | 5.3339 |
| **25** | 6.1108 | 202.89 | 0.0026 | -2.5432 | **55** | 6.8226 | 181.73 | 0.0056 | 12.2620 |
| **26** | 6.1665 | 201.06 | 0.0008 | -0.7818 | **56** | 6.8546 | 180.88 | 0.0013 | 3.8261 |
| **27** | 6.1899 | 200.30 | 0.0101 | 7.0489 | **57** | 6.8744 | 180.36 | 0.0075 | -2.2978 |
| **28** | 6.1920 | 200.23 | 0.0026 | -16.0037 | **58** | 6.8854 | 180.07 | 0.0653 | 6.1164 |
| **29** | 6.2157 | 199.47 | 0.0004 | -0.1764 | **59** | 6.8995 | 179.70 | 0.0031 | 0.8885 |
| **30** | 6.2334 | 198.90 | 0.0012 | 2.3291 | **60** | 6.9291 | 178.93 | 0.3988 | -3.7357 |

* R(velocity) 10**-40 erg-esu-cm.

Figure S_61_. Full-length blots of Figure 5


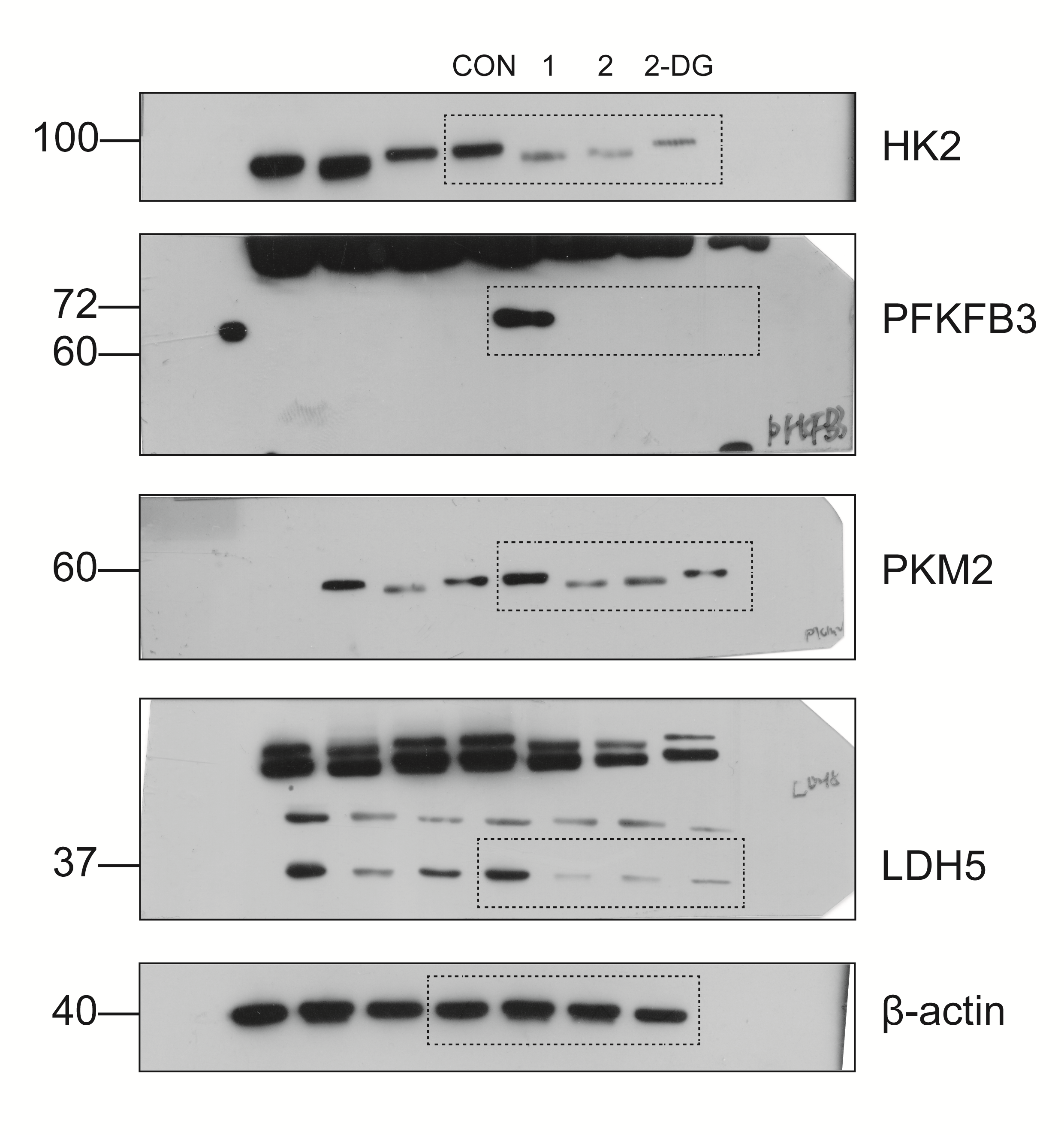


Figure S_62_. Colonies of marine actinomycete *Streptomyces* sp. ZZ406


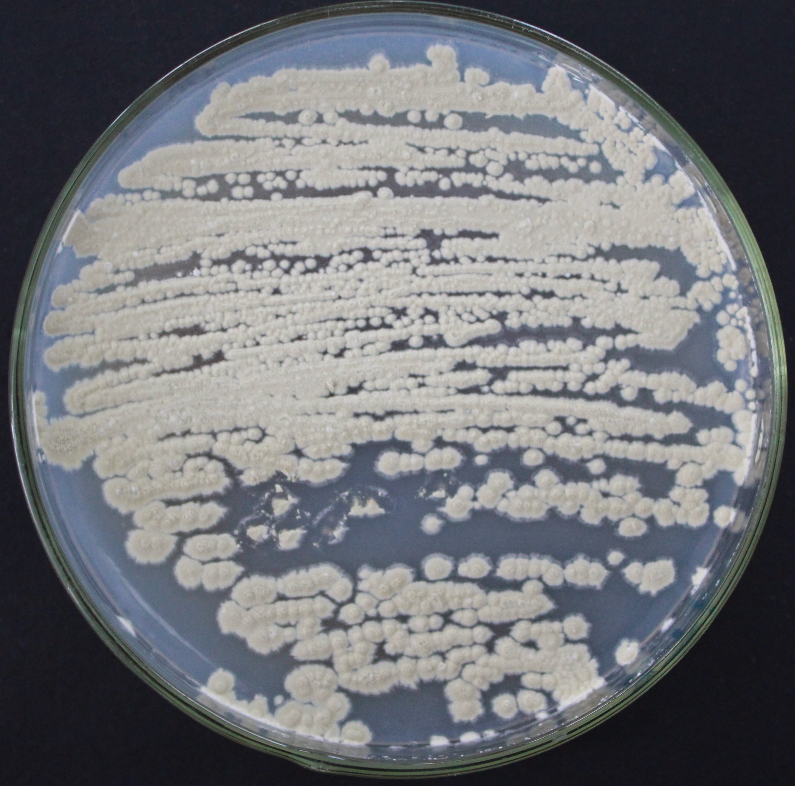

Supplement: Supplementary file 1 — Supplementary Information [file 41598_2017_18484_MOESM1_ESM.doc]
